# Spectral Selective and Low‐Noise Thin‐Film Organic Photodetectors Enabled by Electric‐Field Engineering

**DOI:** 10.1002/adma.75009

**Published:** 2026-09-15

**Authors:** Jingwei Yi, Yi Yang, Vahid Mahdikhah, Yibo Shi, Jordan Shanahan, Apurva Gadikar, Yusen Pei, Xinyun Dong, Jacob P Mauthe, Reece Henry, Kenan Gundogdu, Brendan T. O'Connor, Aram Amassian, Harald Ade, Wei You, Franky So

**Affiliations:** ^1^ Department of Materials Science and Engineering North Carolina State University Raleigh North Carolina USA; ^2^ Organic and Carbon Electronics Laboratories (ORaCEL) North Carolina State University Raleigh North Carolina USA; ^3^ Department of Electrical and Computer Engineering North Carolina State University Raleigh North Carolina USA; ^4^ Department of Chemistry University of North Carolina at Chapel Hill Chapel Hill North Carolina USA; ^5^ Department of Physics North Carolina State University Raleigh North Carolina USA; ^6^ Department of Mechanical and Aerospace Engineering North Carolina State University Raleigh North Carolina USA

**Keywords:** chemical dopants, dark current, electric field, narrowband organic photodetectors, SWIR organic photodetectors

## Abstract

Spectral selective response is a key advantage of organic photodetectors for next‐generation imaging and sensing systems. However, under some practical operating conditions, organic photodetectors are typically subjected to high dark current that limits the device detectivity. Here, we report an electric‐field‐engineering strategy that enables tunable spatial carrier extraction in donor‐acceptor bulk heterojunctions through doping‐induced space‐charge redistribution. By reshaping the internal electric‐field profile, the carrier‐extraction region can be selectively tuned across the active layer. Therefore, the engineered field profile suppresses extraction of injected carriers, contributing to low dark current and high detectivities under reverse bias. Simultaneously, spatial selective extraction of photocarriers enables tuning of the photoresponse bandwidth of thin‐film organic photodetectors without external optical filters. The spectral response can be systematically tuned across multiple material systems, dopants, and device architectures. These results establish electric‐field engineering as a general framework for spectrally selective, high‐detectivity organic photodetection under practically relevant operating conditions.

## Graphical abstract

Molecular doping redistributes the internal electric field in organic photodetectors, providing a new degree of freedom to spatially control carrier transport and collection. Simulations and experiments reveal distinct transport pathways for injected and photogenerated carriers, enabling low dark current under reverse bias and wavelength‐selective charge collection for filter‐free narrowband photodetection across the near‐infrared region.

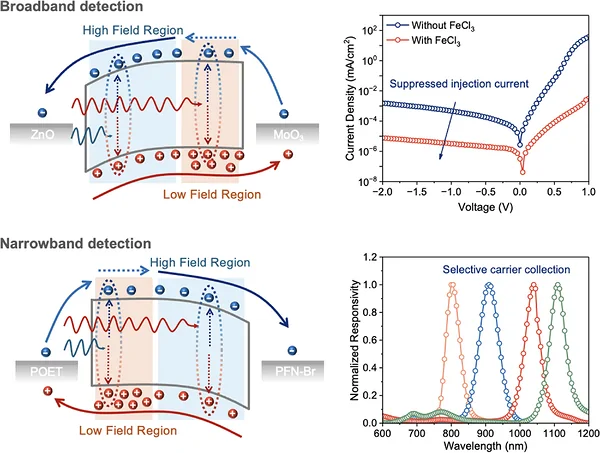

## 1 Introduction

Organic photodetectors (OPDs) have emerged as promising candidates for next‐generation imaging technologies owing to their solution processability, broadband spectral tunability, and compatibility with mechanically flexible platforms [1, 2, 3]. Particular attractive is the prospect of monolithically integrating OPDs with complementary metal‐oxide‐semiconductor (CMOS) readout integrated circuits, which offers a scalable pathway toward compact, low‐cost, and imaging systems that are difficult to realize using conventional inorganic photodetector technologies [4]. Unlike conventional hybrid image sensors that typically require flip‐chip bonding between separately fabricated detector arrays and CMOS backplanes, solution‐processed OPD sensor arrays can be directly fabricated onto prefabricated CMOS substrates without the expensive and complicated flip chip bonding process [5, 6, 7]. Such monolithic integration decouples the trade‐off in optical absorption between active layer and in‐pixel electronics, thereby maximizing optical utilization efficiency and potentially enabling near‐unity effective fill factors [8, 9].

Spectral selectivity is a critical functionality for CMOS image sensors, underpinning color discrimination and wavelength‐resolved detection. Conventional imaging systems typically achieve spectral discrimination by combining broadband photodetectors with external color‐filter arrays, whereas inevitably sacrifices the device efficiency and increases fabrication complexity [10, 11]. Accordingly, extensive efforts have been devoted to realizing intrinsic narrowband OPDs, including wavelength‐selective absorbers such as squaraine [12] and *J*‐aggregated dyes [13], optical microcavity structures [14, 15], multilayer architectures incorporating internal optical filters [16, 17], and more commonly used, charge‐collection narrowing (CCN) [18]. Conventional CCN strategy relies primarily on sufficiently thick active layers together with transport‐limited spatial collection, such that carriers generated outside the effective collection region recombine before extraction. Although effective, this thickness‐dependent approach can impose constraints on film uniformity and carrier transport. More recently, interface doping has also been explored to regulate interfacial band bending for narrowband photoresponse, indicating that selective carrier extraction is governed by the internal electric‐field distribution as it intrinsically defines the effective charge‐collection region inside the device [19, 20]. Therefore, engineering the internal electric‐field profile provides a general framework for tunable spectral response in OPDs. However, a controllable and broadly applicable strategy for modulating the electric‐field distribution in bulk heterojunction systems has not been systematically demonstrated.

Beyond spectral selectivity, a more critical yet often overlooked challenge for OPD‐on‐CMOS integration lies in device operation under practically relevant electric‐field. In practical CMOS image‐sensor architectures, the photodetector frequently experiences non‐zero electric‐field conditions during pixel reset, charge integration, and readout processes. During the reset stage, precharging of the pixel sense node can establish a substantial electric field across the vertically integrated OPD so that the photodiode is constantly under finite bias [21, 22, 23, 24]. However, the vast majority of reported OPDs exhibit optimal performance only near zero bias or under relatively small‐applied voltages [25, 26, 27]. Under reverse‐bias conditions, device performance usually deteriorates because of enhanced carrier injection at electrode/organic interfaces, leading to substantially increased dark current and severe degradation of detectivity [28, 29, 30, 31]. This field‐dependent degradation not only limits the device sensitivity, but also fundamentally constrains the implementation of OPDs in high‐performance CMOS imaging architectures where low‐noise operation under electric fields is indispensable. Consequently, many OPDs that demonstrate excellent figures of merit under laboratory low‐bias conditions fail to maintain comparable detectivity under realistic operating conditions in CMOS imaging systems.

Here, we introduce an electric‐field‐engineering device principle that directly modulates spatial carrier extraction within donor‐acceptor bulk heterojunctions through dopant‐induced redistribution of the space charge under reverse bias. This engineered field profile governs where photogenerated carriers are efficiently extracted, thereby linking spectral selectivity directly to field‐controlled charge‐collection dynamics. When the high‐field region is positioned near the illuminated interface, photocarriers generated throughout the active layer are efficiently extracted, resulting in broadband photodetection. In contrast, shifting the high‐field region toward the back electrode spatially confines collection to carriers generated only deeper within the film, enabling intrinsically narrowband and filter‐free spectral response. Importantly, the same field‐engineering strategy simultaneously suppresses reverse bias‐induced carrier injection, enabling detectivities exceeding 10^12^ Jones under −2 V reverse bias. Furthermore, the spectral response can be systematically tuned from approximately 800 to 1100 nm across multiple material systems, chemical dopants, and device architectures with a full‐width‐at‐half‐maximum (FWHM) of ∼50 nm, demonstrating the generality and versatility of this electric‐field redistribution framework. Together with the demonstrated operational stability under reverse bias, these results establish the device‐level feasibility of bulk electric‐field engineering for spectrally selective, low‐noise organic photodetection under practically relevant bias conditions.

## 2 Results and Discussion

### 2.1 Origin of Electric‐Field Redistribution in Doped Bulk Heterojunctions

To elucidate the origin of electric‐field redistribution in doped bulk heterojunctions, we selected FeCl_3_ as a *p*‐dopant additive and PM6:Y6 as bulk heterojunctions in this work. The molecular structures of PM6 and Y6 (Figure 1a) contain extended π‐conjugated backbones and electron‐rich heteroatoms that can facilitate charge‐transfer interactions with strong *p*‐type dopants such as FeCl_3_ [32, 33]. Therefore, FeCl_3_ incorporation induces measurable changes in both the electronic and structural properties of the blend. UV‐vis absorption spectra reveal bleaching of the characteristic absorption features upon doping (Figure S1), and Fourier‐transform infrared (FTIR) spectroscopy shows the emergence of polaron‐associated absorption bands (Figure S2), indicating the generation of charged species within the active layer. X‐ray photoelectron spectroscopy (XPS) further confirms electronic perturbation of the donor‐acceptor matrix after FeCl_3_ incorporation (Figure S3). In addition, Grazing‐incidence wide‐angle x‐ray scattering (GIWAXS) measurement suggests the bulk heterojunction morphology is preserved upon doping (Figures S4, S5, Note S1, and Table S1).

**FIGURE 1:**
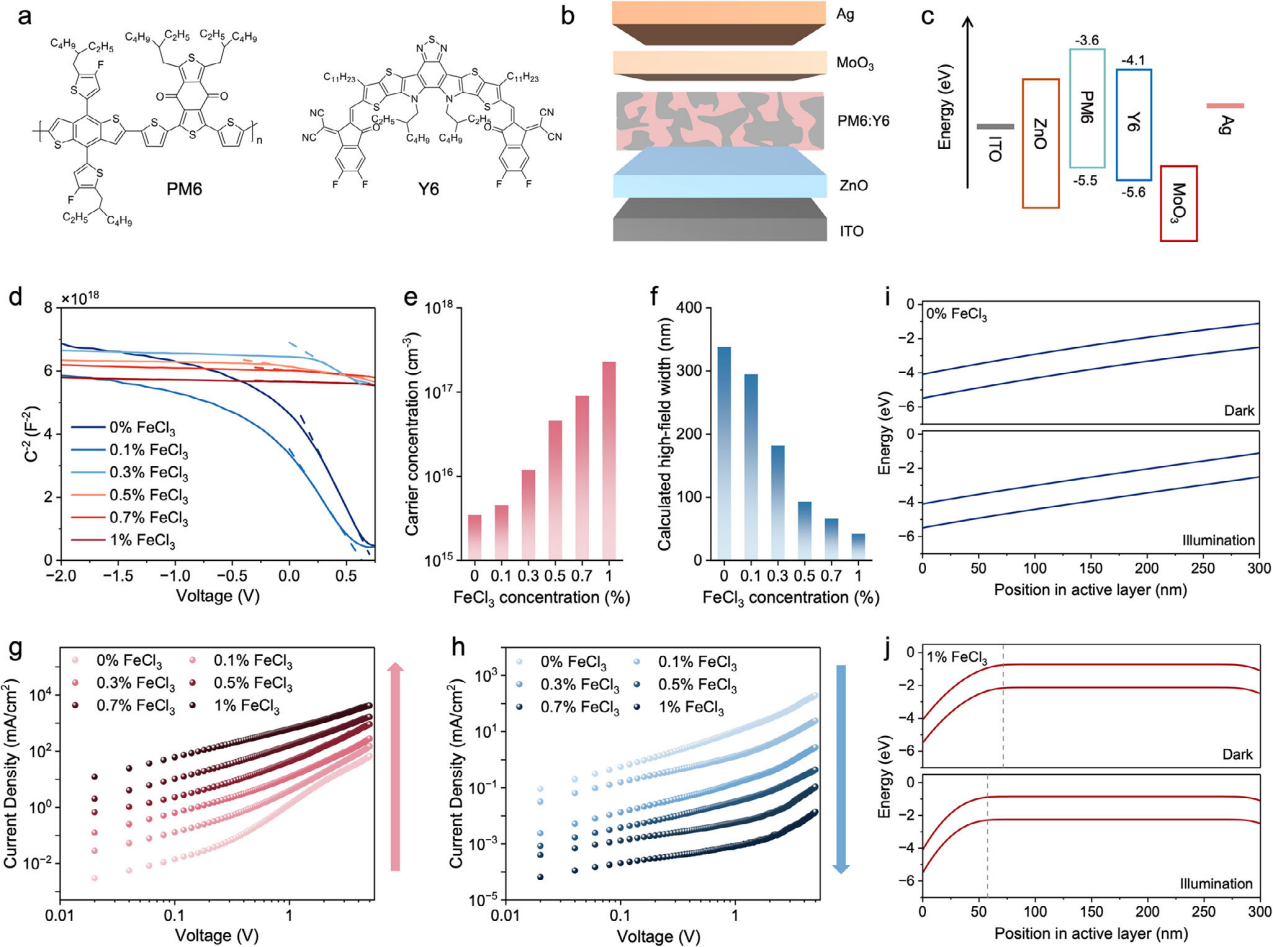
(a) Molecular structure of PM6 and Y6. (b) Schematic illustration of the inverted OPD device structure consisting of ITO/ZnO/PM6:Y6/MoO_3_/Ag. (c) Energy diagram of each material. (d) Mott‐Schottky plots of devices with varying FeCl_3_ concentrations. The solid lines represent measured data points and dashed lines represent linear fitting. Calculated (e) carrier concentration and (f) effective high‐field region width as a function of FeCl_3_ concentration. (g) Hole‐only and (h) electron‐only space‐charge‐limited‐current (SCLC) characteristics. Simulated band diagrams and electric‐field distributions of (i) undoped and (j) 1 wt% FeCl_3_‐doped devices under dark and illumination at −2 V. The solid lines represent the simulated electrostatic potential, and dashed lines mark the extent of the high‐field region.

The internal electric field in an organic semiconductor is governed by the distribution of spatial charges according to Poisson's equation [34],

(1) $$\;\frac{\mathrm{dE}(x)}{\mathrm{dx}}=\frac{\rho (x)}{\varepsilon_{r}}\;$$

where *E*(*x*) is the electric field, *ρ*(x) is the local charge density, and *ε_r_* is the dielectric constant. Understanding the origin of electric‐field redistribution therefore requires identifying how FeCl_3_ modifies both the carrier concentration and carrier transport within the active layer.

To quantify these parameters, inverted photodetectors with the structure ITO/ZnO/PM6:Y6+FeCl_3_ (300 nm)/MoO_3_/Ag were fabricated (Figure 1b), and the corresponding energy‐level diagram is shown in Figure 1c. Capacitance‐voltage measurements were performed in the dark condition to evaluate the carrier concentration through Mott‐Schottky analysis. Because organic bulk heterojunctions can exhibit energetic disorder, ambipolar transport, trap‐mediated capacitance, and spatially nonuniform doping, the extracted values are treated as equivalent carrier concentrations and effective high‐field‐region widths rather than parameters of an ideal inorganic depletion layer (Note S2). To minimize slow trap‐related contributions, capacitance‐frequency and phase‐angle measurements were first used to identify a stable, predominantly capacitive window, which is from approximately 1 to 10 kHz (Figure S6). As a result, Mott‐Schottky measurements at 1, 5, and 10 kHz yield closely comparable linear slopes, differing by less than 5%, and 1 kHz was then selected as the representative frequency (Figure S7). Device‐to‐device statistics from independently fabricated devices were further used to quantify the error in the extracted parameters (Table S2). As shown in Figure 1d, the incorporation of FeCl_3_ results in a pronounced change in the Mott‐Schottky response. The extracted carrier concentration increases from 3.45 × 10^15^ cm^−3^ in the undoped device to 2.29 × 10^17^ cm^−3^ at 1 wt% FeCl_3_ doping (Figure 1e), accompanied by narrowing of the high‐field region. In the undoped device, the high‐field region extends across ∼338 nm at 0 V, which is comparable to the full active‐layer thickness, suggesting a nearly uniform internal electric field profile. In contrast, the high‐field region is substantially reduced to ∼42 nm at 1 wt% FeCl_3_ concentration (Figure 1f), directly confirming strong internal field redistribution in dark condition.

Hole‐only (ITO/PEDOT:PSS/PM6:Y6/MoO_3_/Ag) and electron‐only (ITO/ZnO/PM6:Y6/Al) devices were fabricated to evaluate the transport properties using the space‐charge‐limited‐current (SCLC) model. As shown in Figure 1g,h, FeCl_3_ incorporation produces markedly different effects on hole and electron transport. The hole mobility (*µ*_h_) increases by more than two orders of magnitude, while the electron mobility (*µ*_e_) decreases substantially with increasing dopant concentration (Figure S8). The extracted mobility results indicate that FeCl_3_ modifies the carrier transport characteristics of the bulk heterojunction and may influence charge accumulation and carrier extraction under device operating conditions.

To establish how the experimental carrier concentrations and transport properties influence the internal electric field, drift‐diffusion simulations were performed using the carrier densities extracted from Mott‐Schottky analysis together with the carrier mobilities obtained from SCLC measurements (see Note S3 for detailed discussion). We first simulated the band diagrams under dark conditions to evaluate the effect of doping‐induced carrier accumulation in the absence of photogenerated carriers. As shown in Figure 1i,j, the undoped device exhibits a nearly uniform electric‐field distribution, with the electrostatic potential dropping gradually across the entire active layer. In contrast, the FeCl_3_‐doped device displays a strongly redistributed electric‐field profile, where most of the potential drop is confined to a narrow region adjacent to the cathode. This redistribution in the dark originates from the substantial increase in carrier concentration by doping rather than the mobility imbalance, as shown in Figure S9. Upon FeCl_3_ incorporation, the carrier concentration increases by nearly two orders of magnitude, introducing a large population of mobile holes within the bulk heterojunction. Under the built‐in electric field, holes redistribute and accumulate near the anode, generating space charge that partially screens the local electric field. As a result, the electrostatic potential becomes nearly flat over a large portion of the active layer, while the remaining potential drop is concentrated near the cathode, giving rise to the observed field redistribution.

We next simulated the band diagrams under illumination for the undoped and FeCl_3_‐doped devices. Notably, the electric‐field profiles obtained under illumination are highly similar to those calculated under dark conditions for both devices. For the FeCl_3_‐doped device, only a slight additional narrowing of the high‐field region is observed under illumination. We attribute this difference to the increased mobility asymmetry introduced by FeCl_3_ doping, which can promote unbalanced extraction of photogenerated carriers and thereby enhance space‐charge accumulation during device operation. However, the overall similarity between the dark and illuminated field profiles indicates that the redistributed electric field is still predominantly established by doping‐induced mobile carrier accumulation, whereas illumination‐induced transport effects provide only a minor enhancement of the space‐charge effect.

### 2.2 Consequences of Electric‐Field Redistribution on Charge Extraction

Having established the physical origin of the redistributed electric field, we next investigate its impact on charge extraction. The light current density–voltage (*J–V*) characteristics of PM6:Y6 photodetectors with different FeCl_3_ concentrations were measured. Compared with the nearly ideal photocurrent response of the undoped device, the light *J–V* curves evolve toward a pronounced *S*‐shaped characteristic upon doping (Figure S10). Such *S*‐shaped behavior is commonly associated with space‐charge‐limited carrier extraction, where accumulated charges distort the internal electric‐field profile and impede charge transport through the active layer [35]. The emergence of this feature therefore provides additional experimental evidence for substantial space‐charge accumulation in the doped devices, consistent with the drift‐diffusion simulations discussed above.

The carrier‐extraction process is schematically illustrated in Figure 2a. In the undoped device, as we mentioned, the electric field extends across nearly the entire active layer, allowing photogenerated carriers throughout the film to be efficiently extracted. In contrast, FeCl_3_ incorporation compresses the electric field into a much narrower region adjacent to the cathode due to space‐charge‐induced screening. Under these conditions, only carriers generated within or near the high‐field region can be efficiently collected, whereas carriers generated outside this region are more susceptible to recombination before extraction. To evaluate the spatial distribution of photocarrier generation profile and correlate with the field distribution, transfer‐matrix method (TMM) simulations were performed. The in‐plane refractive index *n*(λ) and the extinction coefficient *k*(λ) used in the simulation were experimentally measured by spectroscopic ellipsometry (Figure S11). As shown in Figure S12, most photocarriers are generated within approximately the first 100 nm from the illuminated side of the device. Therefore, an efficient carrier extraction critically depends on the spatial overlap between the photogeneration profile and the high‐field region.

**FIGURE 2:**
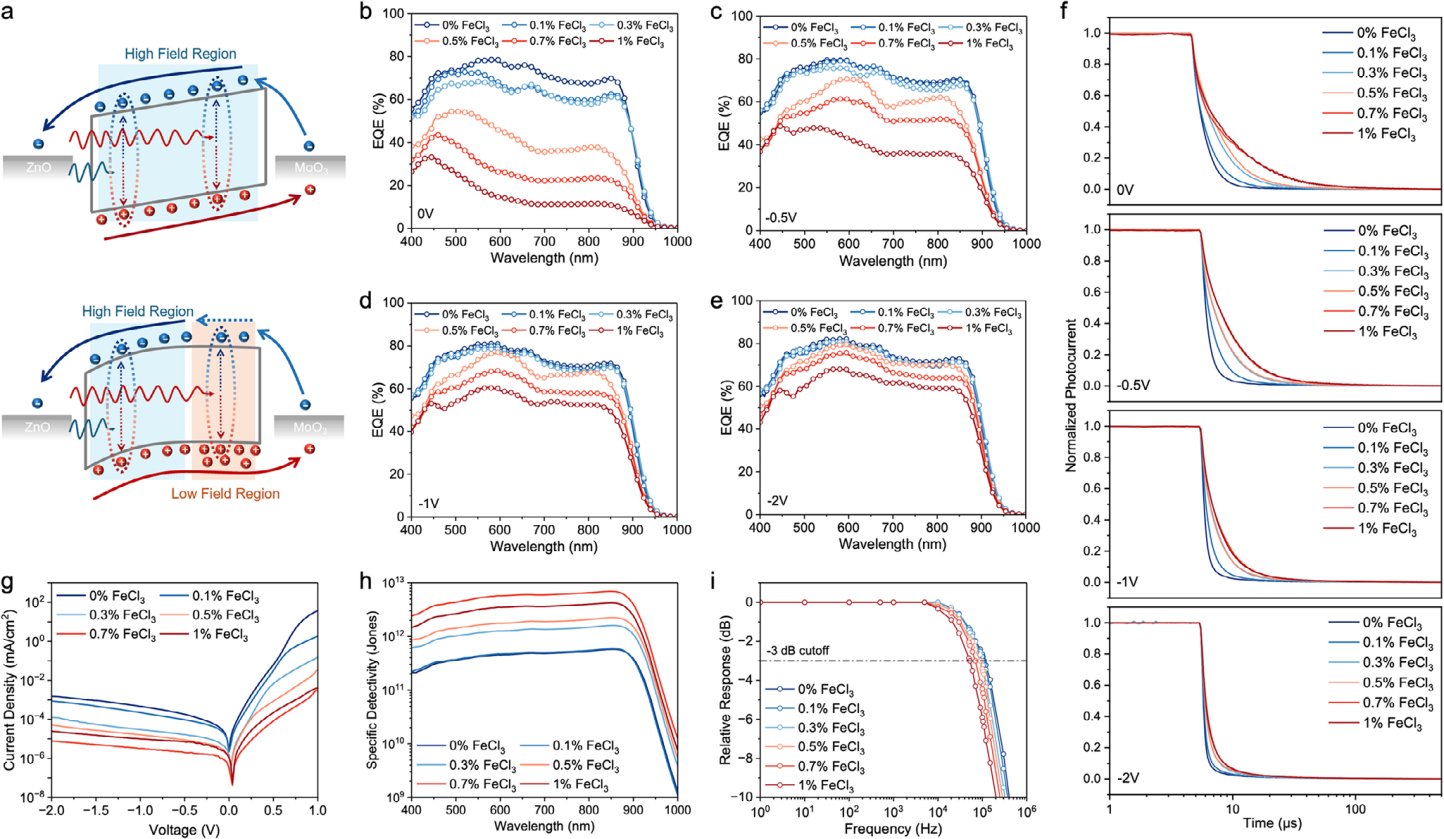
(a) Schematic illustration of the internal electric‐field distribution before (top) and after (bottom) FeCl_3_ incorporation in inverted architecture. The blue and red arrow lines indicate electron and hole flow path, respectively. External quantum efficiency (*EQE*) spectra of PM6:Y6 OPDs with varying FeCl_3_ concentrations measured at reverse biases of (b) 0 V, (c) −0.5 V, (d) −1 V, and (e) −2 V. (f) Normalized transient photocurrent (TPC) responses measured at different reverse biases. (g) Dark current density–voltage (*J–V*) characteristics of devices with different FeCl_3_ concentrations. (h) Specific detectivity (*D* ^*^) spectra calculated from measured noise current (see Methods for detailed equation). (i) Normalized photoresponse as a function of input signal frequency. Horizontal dashed lines mark the cut‐off frequencies obtained at −3 dB under −2 V reverse bias. The light source used here is a 910 nm LED with the intensity of 36 mW/cm^2^.

This carrier‐extraction model is directly reflected in the external quantum efficiency (*EQE*) characteristics. As shown in Figure 2b, devices containing more than 0.5 wt% FeCl_3_ exhibit a pronounced reduction in *EQE* under short‐circuit conditions. Upon applying reverse bias, however, the *EQE* progressively recovers for all doped devices (Figures 2c–e). At −2 V, devices with 0–0.5 wt% FeCl_3_ exhibit nearly identical *EQE* spectra, while 0.7 wt% FeCl_3_ device exhibits only a small reduction in *EQE* relative to the undoped device. The recovery of *EQE* under reverse bias can be understood from the evolution of the electric‐field profile. Drift‐diffusion simulations and the calculated high‐field region widths reveal that the internal electric field gradually expands with increasing reverse bias (Figures S13, S14, and Table S2), thereby increasing its overlap with the photogeneration profile. This improved alignment between the optical‐generation profile and the high‐field region enables efficient field‐assisted carrier extractions and restores the *EQE* under reverse bias.

In addition to the spatial mismatch between the photogeneration profile and the high‐field region, changes in carrier transport due to doping also contribute to the reduced *EQE* at low bias. Electron‐only SCLC measurements reveal a substantial decrease in electron mobility upon FeCl_3_ doping, indicating enhanced mobility asymmetry in the blend. Consequently, under short‐circuit conditions, charge extraction becomes transport‐limited, resulting in reduced carrier collection efficiency and *EQE*. In contrast, applying a reverse bias increases the electric field and enhances carrier drift, thereby improving charge collection. To further study this effect, transient photocurrent (TPC) measurements were performed (Figure 2f). At 0 V, FeCl_3_‐doped devices exhibit substantially slower photocurrent decay than the undoped device, indicating slower carrier extraction kinetics. However, at −2 V, the decay dynamics become significantly faster and nearly identical across all doping concentrations, suggesting that the extraction limitation introduced by FeCl_3_ doping is largely mitigated under strong bias conditions.

### 2.3 Dark Current Suppression in Inverted Devices

As discussed above, Mott‐Schottky analysis and drift‐diffusion simulations reveal that FeCl_3_ incorporation leads to substantial accumulation of mobile holes near the anode even in the dark condition, producing a low‐field region over a large portion of the active layer. This field profile is expected to influence not only the collection of photogenerated carriers but also the transport of injected carriers under reverse bias. In the PM6:Y6 devices, electron injection from the MoO_3_/Ag contact was identified as the dominant dark‐current contribution under reverse bias (Figure S15 and Note S4). Upon FeCl_3_ doping, the emergence of the low‐field region close to the anode can substantially limit long‐range electron transport across a large portion of the active layer, thereby suppressing injection‐driven dark current. Importantly, we note that injected carriers and photogenerated carriers experience fundamentally different transport pathways within the redistributed electric‐field profile. Injected electrons must diffuse across the extended low‐field region before reaching the high‐field region, where they are susceptible to recombination with the accumulated holes associated with the space‐charge distribution. In contrast, photogenerated carriers created within or near the high‐field region are efficiently extracted by drift under the strong local electric field, resulting in a much lower probability of recombination. Consequently, the redistributed field can selectively suppress injection‐driven dark current while maintaining efficient photocarrier extraction.

The evolution of reverse‐bias dark current with varying FeCl_3_ concentrations is shown in Figure 2g. At moderate doping concentrations (0–0.7 wt%), *J*_d_ decreases substantially under reverse bias, reaching a minimum value of 9 × 10^−6^ mA/cm^2^ at −2 V for the 0.7 wt% FeCl_3_ device, representing more than two orders of magnitude reduction relative to the undoped device. The reproducibility and operational stability of the field‐engineered devices were also evaluated (Figures S16–S18). Statistical analysis across multiple independently fabricated devices shows narrow distributions in *J*_d_ and *EQE*, demonstrating good device‐to‐device reproducibility. Moreover, the FeCl_3_‐doped devices exhibit storage stability comparable to that of the undoped devices after 2 weeks under storage, thermal aging at 60°C, and continuous −2 V reverse‐bias operation. At higher doping concentrations (1–3 wt%), *J*_d_ starts to increase, suggesting enhanced carrier injection at excessive doping levels (Figure S19). The reduced forward‐bias current is consistent with the accumulation of space charge under dark conditions, which suppresses carrier injection and promotes space‐charge‐limited transport. To further evaluate injection suppression, electrochemical impedance spectroscopy (EIS) was performed under dark conditions at −2 V bias over a frequency range of 1 Hz–1 MHz. As shown in Figure S20, the extracted shunt resistance (*R*_sh_), which is closely associated with leakage current, increases with FeCl_3_ concentration up to 0.7 wt% (11.8 MΩ) before decreasing at higher doping concentrations, consistent with the observed dark‐current evolution. Temperature‐dependent current density–voltage (*J–V*) measurements further reveal that the activation energy (*E*_a_) increases from 0.16 eV in the undoped device to 0.30 eV at 0.7 wt% FeCl_3_ doping before decreasing at higher concentrations (Figure S21). The increased activation barrier at moderate doping concentrations further supports suppression of injection‐driven transport induced by field redistribution.

To distinguish field redistribution from other doping‐induced changes, we additionally examined morphology and sub‐gap states across the same doping series. GIWAXS and atomic force microscope (AFM) measurements show no substantial change in molecular packing, surface morphology, or roughness, while photothermal deflection spectroscopy (PDS) yields nearly unchanged Urbach energies (*E*_u_) and comparable integrated sub‐gap absorption (Figures S22 and S23). These results indicate that the large reduction in dark current is unlikely to originate primarily from changes in morphology or trap density. More directly, extended dark *J–V* measurements at higher reverse bias reveal a characteristic transition in the doped devices. The initially suppressed dark current rises rapidly above a characteristic reverse bias and progressively approaches that of the undoped device (Figure S24). This transition tracks the bias‐dependent expansion of the calculated high‐field region. At lower reverse bias, an extended low‐field region remains and blocks long‐range transport of injected electrons. As reverse bias increases, the high‐field region expands until the low‐field blocking region is largely removed, at which point the dark current of doped and undoped devices converge. Therefore, this bias‐dependent correspondence provides direct experimental support linking the low‐field region to dark‐current suppression.

Detectivity is a key figure of merit for OPDs. Based on the measured dark current, the shot‐noise‐limited detectivity of the 0.7 wt% FeCl_3_ device reaches 4.4 × 10^13^ Jones at 850 nm under −2 V reverse bias (Figure S25). To more accurately evaluate the device performance, noise‐current measurements were performed by considering contributions from 1/*f* noise, thermal noise, and shot noise. The calculated shot‐noise contribution decreases with doping because of the reduced dark current, and the thermal‐noise contribution decreases because the shunt resistance extracted from impedance measurements increases. In addition, fitting of the low‐frequency spectra shows that the 1/*f*‐noise contribution is also suppressed after FeCl_3_ incorporation (Figure S26). Based on the measured noise spectrum, the specific detectivity (*D* ^*^) was calculated to be 6.9 × 10^12^ Jones at 850 nm under −2 V for the 0.7 wt% FeCl_3_ device (Figure 3h). A comparison with previously reported broadband OPDs operated under reverse bias is summarized in Table S3. The linear dynamic range exceeds six orders of magnitude (Figure S27), while the −3 dB cutoff frequency reaches approximately 100 kHz (Figure 2i). In addition, rise and fall times below 10 µs are achieved (Figure S28), demonstrating that fast response can be maintained together with low‐noise operation under reverse bias.

**FIGURE 3:**
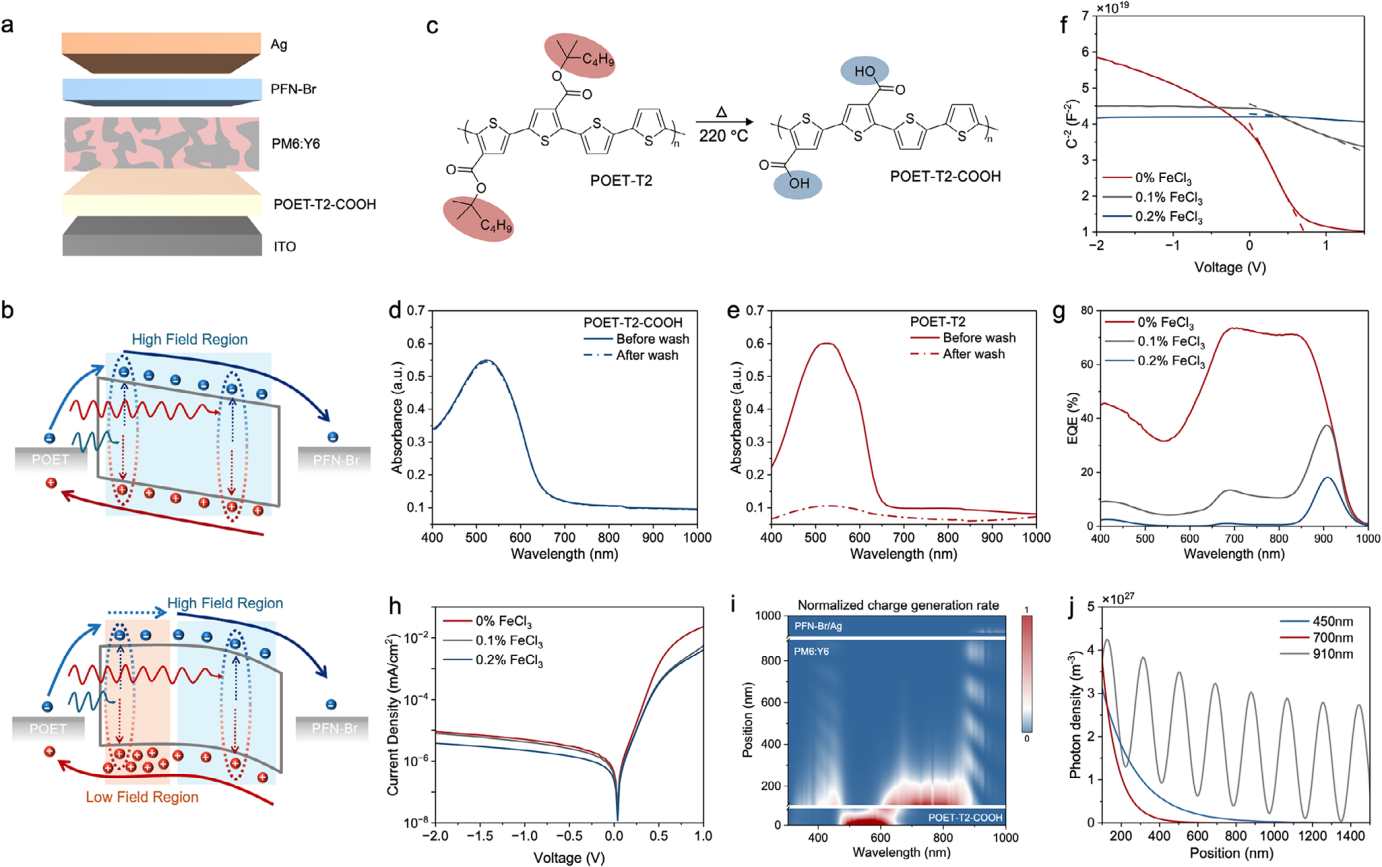
(a) Schematic illustration of the conventional OPD device structure consisting of ITO/POET‐T2‐COOH/PM6:Y6/PFN‐Br/Ag. (b) Schematic illustration of the electric‐field distribution in the conventional architecture before and after FeCl_3_ incorporation. Upon doping, the effective high‐field region shifts toward the back side of the active layer, while a low‐field region forms near the illuminated interface, suppressing the collection of carriers generated by strongly absorbed short‐wavelength light. (c) Chemical structure transformation from POET‐T2 to POET‐T2‐COOH induced by thermal cleavage of the side chains during annealing. UV‐vis absorption spectra of pristine (d) POET‐T2‐COOH and (e) POET‐T2 films before and after chloroform washing. (f) Dark current density–voltage (*J–V*) characteristics of conventional PM6:Y6 devices with varying FeCl_3_ concentrations. (g) *EQE* spectra measured at −2 V. (h) Mott‐Schottky plots of conventional devices with different FeCl_3_ concentrations. (i) Simulated spatial photogeneration profile within the active layer as a function of wavelength. Strongly absorbed short‐wavelength photons are confined near the illuminated interface, whereas long‐wavelength photons penetrate deeper into the active layer. (j) Simulated photogeneration density profiles for 450, 700, and 910 nm illumination.

### 2.4 Electric‐Field Redistribution for Narrowband Photodetectors

We demonstrated that electric‐field redistribution not only suppresses injection‐driven dark current, but also governs which spatial regions of the active layer contribute effectively to photocurrent generation. Because photogeneration in organic semiconductors is spatially nonuniform, modifying the position of the high‐field region provides a direct route to engineer spectral selectivity through field‐controlled carrier extraction.

In the inverted architecture discussed above, *p*‐type doping generates an extended low‐field region near the MoO_3_/Ag contact while maintaining the high‐field region near the illuminated side of the device. Under these conditions, most photogenerated carriers remain spatially overlapped with the high‐field region, preserving broadband photoresponse. In contrast, reversing the device polarity in the conventional architecture (Figure 3a) correspondingly reverses the electric‐field profile. As illustrated schematically in Figure 3b, *p*‐type doping spatially confines the high‐field region near the back side of the active layer, away from the illuminated electrode. Consequently, carriers generated near the front surface by strongly absorbed short‐wavelength photons are inefficiently extracted, while long‐wavelength photons penetrate deeper into the active layer and generate carriers closer to the back‐side high‐field region, enabling preferential carrier extraction at longer wavelengths.

To test the universality of this device architecture, conventional OPDs with the structure of ITO/POET‐T2‐COOH/PM6:Y6+FeCl_3_ (800 nm)/PFN‐Br/Ag were fabricated. The POET‐T2‐COOH interlayer was employed in place of PEDOT:PSS because of its favorable energy‐level alignment and stronger electron‐blocking capability (Figure S29) [36]. The interlayer also exhibits excellent solvent resistance after thermal conversion from POET‐T2 to POET‐T2‐COOH through side‐chain cleavage (Figures 3c–e), enabling robust sequential solution processing of the active layer. AFM and contact‐angle measurements further confirm the uniform morphology and solvent robustness of the interlayer (Figures S30 and S31).

The *J–V* characteristics of the conventional devices are shown in Figure 3f. Similar to the inverted architecture, FeCl_3_ incorporation suppresses reverse‐bias dark current, consistent with the injection‐suppression mechanism established above. At an optimal FeCl_3_ concentration of 0.2 wt%, the devices maintain low dark current densities on the order of nA/cm^2^ at −2 V. Notably, devices employing the POET‐T2‐COOH interlayer exhibit more than one order of magnitude lower dark current compared with PEDOT:PSS‐based devices (Figure S32), reflecting the benefit of the stronger electron‐blocking capability. Importantly, the *EQE* spectra evolve from broadband to narrowband response upon FeCl_3_ incorporation (Figure 3g). The voltage‐dependent *EQE* evolution is shown in Figure S33. At −2 V, the 0.2 wt% FeCl_3_ device exhibits a pronounced narrowband response centered at 910 nm with a FWHM of 65 nm. The device‐to‐device reproducibility of *J*_d_​, *EQE*, and the peak position, together with the stability data, is summarized in Figures S34–S38. The corresponding shot‐noise‐limited detectivity exceeds 10^13^ Jones over 0 to −2 V (Figure S39), demonstrating that field‐controlled spectral selectivity can be achieved while maintaining high detection sensitivity.

To further elucidate the narrowband mechanism, Mott‐Schottky analysis was again performed on the conventional devices (Figures 3h, S40, and S41). The extracted high‐field region widths at −2 V decrease progressively from approximately 1547 to 304 nm with increasing FeCl_3_ concentration, indicating strong confinement of the effective carrier‐extraction region. The computed band diagrams are shown in Figure S42. Comparison between the extracted high‐field‐region widths and the simulated optical‐generation profiles (Figures 3i and S43) reveals that short‐wavelength photons (450 and 700 nm) are absorbed predominantly near the illuminated surface, whereas long‐wavelength photons (910 nm) penetrate throughout the active layer (Figure 3j). Consequently, in the 0.2 wt% FeCl_3_ device, only carriers generated by deeply penetrating long‐wavelength photons stay spatially overlapped with the confined high‐field region and can therefore be efficiently extracted, giving rise to the observed intrinsically narrowband photoresponse. Independent evidence for this spatially asymmetric collection profile is provided by illumination‐direction‐dependent *EQE* measurements using a semitransparent device with the structure ITO/POET‐T2‐COOH/PM6:Y6+0.2 wt% FeCl_3_ (800 nm)/PFN‐Br/Ag (15 nm). Under bottom illumination through ITO, the device exhibits the narrowband response described above, whereas illumination from the semitransparent Ag side recovers a broadband photoresponse (Figure S44). Because the active‐layer thickness, doping concentration, electrodes, and applied bias are unchanged, the strong dependence on illumination direction cannot be explained by intrinsic absorption or bulk composition. Instead, it provides independent experimental evidence for spatially nonuniform carrier‐collection efficiency across the active layer, consistent with a high‐field collection region localized toward the back side of the conventional device.

### 2.5 Generality of the Electric‐Field‐Engineering Framework

To demonstrate the generality of the electric‐field‐engineering strategy, we extended this approach to multiple non‐fullerene acceptor systems spanning different spectral regions. Three additional donor‐acceptor systems, PM6:ITIC‐4F, PTB7‐Th:SiOTIC‐4Cl, and PTB7‐Th:COTIC‐4Cl, were selected to target photoresponse windows centered near ∼800, ∼1000, and ∼1100 nm, respectively (Figures 4a and S45). Upon FeCl_3_ incorporation, all investigated systems exhibit a clear transition from broadband to narrowband photoresponse (Figure S46), while simultaneously maintaining low *J_d_* under reverse bias (Figure S47). Frequency‐dependent noise measurements further reveal low noise current across all devices (Figure S48), contributing to their high detectivity. As a result, by selecting different donor‐acceptor combinations, high detectivity narrowband OPDs can be realized across a broad spectral range from ∼800 to ∼1100 nm (Figures 4b–e). Notably, whereas most previously reported narrowband OPDs achieve high detectivity only at zero or low bias, the devices developed here maintain detectivities exceeding 10^12^ Jones even under −2 V reverse bias (Table S4).

**FIGURE 4:**
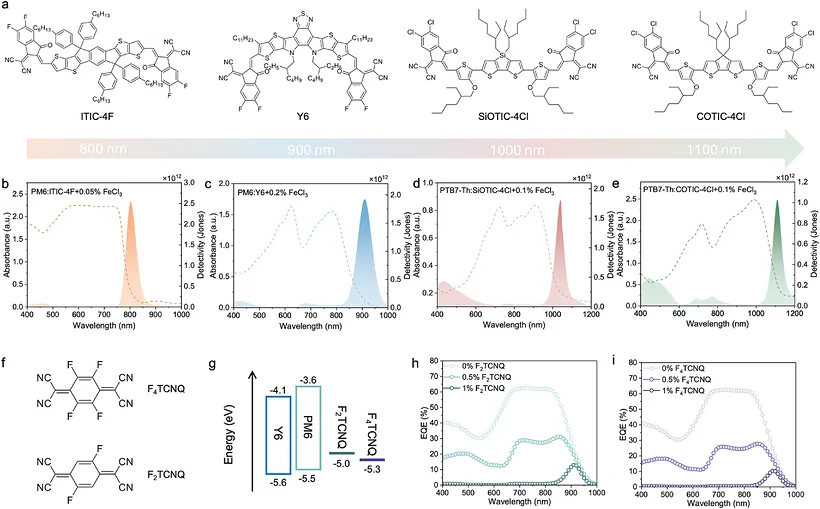
(a) Molecular structures of the non‐fullerene acceptors ITIC‐4F, Y6, SiOTIC‐4Cl, and COTIC‐4Cl used to achieve narrowband photodetection across different wavelength regions. UV‐vis absorption spectra (dashed curves) and corresponding specific detectivity spectra (filled curves) of FeCl_3_‐doped narrowband OPDs based on (b) PM6:ITIC‐4F+0.05 wt% FeCl_3_, (c) PM6:Y6+0.2 wt% FeCl_3_, (d) PTB7‐Th:SiOTIC‐4Cl+0.1 wt% FeCl_3_, and (e) PTB7‐Th:COTIC‐4Cl+0.1 wt% FeCl_3_. (f) Molecular structures of the molecular *p*‐type dopants F_2_TCNQ and F_4_TCNQ. (g) Energy level diagram of PM6, Y6, F_2_TCNQ, and F_4_TCNQ. The deeper lowest unoccupied molecular orbital (LUMO) level of F_4_TCNQ indicates stronger electron‐accepting capability and enhanced *p*‐doping strength. *EQE* spectra of PM6:Y6 devices doped with varying concentrations of (h) F_2_TCNQ and (i) F_4_TCNQ.

To further evaluate the universality of the field‐engineering framework with respect to dopant selection, two additional *p*‐type dopants, F_2_TCNQ and F_4_TCNQ, were investigated in PM6:Y6 devices (Figure 4f). Density functional theory (DFT) calculations of electrostatic surface potentials and energy levels are shown in Figure S49. As illustrated in the energy level diagram (Figure 4g), F_4_TCNQ exhibits a deeper LUMO level than F_2_TCNQ, consistent with its stronger electron‐accepting capability and higher doping strength. Both dopants induce a clear transition from broadband to narrowband photoresponse with increasing doping concentration. Moreover, devices incorporating F_4_TCNQ exhibit lower *EQE* at comparable doping concentrations relative to F_2_TCNQ‐based devices (Figure 4h,i), consistent with stronger electric‐field redistribution induced by the stronger dopant. These results indicate that the narrowband‐response mechanism is not unique to FeCl_3_ but can be generalized across chemically distinct *p*‐type dopants with different doping strengths.

Finally, the electric‐field‐engineering strategy was further extended to different device architectures through *n*‐type doping in inverted OPDs. To demonstrate this concept, P(Cy)_3_ was employed as an *n*‐type dopant in PM6:Y6 inverted devices. DFT calculations reveal a shallow LUMO level and low electrostatic potential localized on the phosphorus atom, consistent with its electron‐donating character (Figure S50). Upon incorporation of 1 wt% P(Cy)_3_, the devices also exhibit a clear transition from broadband to narrowband photoresponse (Figure S51). Collectively, we demonstrated that doping‐induced electric field redistribution represents a general framework for intrinsically narrowband OPDs that is broadly applicable across different material systems, dopant species, and device architectures.

## 3 Conclusion

We report an electric‐field‐engineering strategy that enables high‐performance organic photodetection under reverse‐bias operation through controlled redistribution of the internal electric field in the donor‐acceptor bulk heterojunction layer. In this work, charge generation due to molecular doping leads to modification of the internal electric field. Our results demonstrate that substantial field redistribution can already be established under dark conditions through doping‐induced carrier accumulation. Mott‐Schottky analysis reveals a nearly two‐order‐of‐magnitude increase in carrier concentration upon FeCl_3_ incorporation, accompanied by a pronounced narrowing of the high‐field region. Combined with drift‐diffusion simulations, these observations indicate that the increased population of mobile carriers creates a redistributed space‐charge profile that reshapes the electrostatic potential leading to a spatially nonuniform electric‐field distribution consisting of an extended low‐field region and a spatially confined high‐field region. This field modification suppresses injection‐driven dark current through the low‐field region while preserving efficient photocarrier extraction within the confined high‐field region.

Consequently, *p*‐doped inverted‐architecture devices exhibit broadband photodetection with detectivities exceeding 10^12^ Jones at −2 V, together with fast response and wide linear dynamic range. More importantly, spectral selectivity can be directly achieved through the spatial positioning of the high‐field region relative to the optical generation profile, enabling a transition from broadband to intrinsically narrowband response in conventional architectures. By selecting different non‐fullerene acceptors, the spectral response can be systematically tuned across ∼800–1100 nm. Unlike conventional CCN that relies primarily on thick active layers, or interface modification for band bending, our approach uses bulk doping to directly engineer the electric‐field distribution across the photoactive layer. Importantly, the same field profile simultaneously suppresses dark‐carrier transport and controls photocarrier collection, establishing bulk electric‐field engineering as a general strategy for achieving low‐noise and spectrally selective organic photodetection.

## 4 Methods

### 4.1 Fabrication of Inverted Devices

The patterned ITO glass substrates (purchased from KINTEC) were cleaned with a surfactant solution and then soaked in deionized water, acetone and isopropanol in an ultrasonic bath for 15 min each. After UV‐ozone treatment for 15 min, the ZnO precursor solution was spin coated onto the ITO substrate and annealed at 150°C for 50 min in the air to form a ZnO film of around 20–30 nm. The PM6:Y6 (donor: acceptor  =  1:1.2) was dissolved in chloroform (25 mg/ml in total) and stirred at 50°C for over 2 h. An additive, 1‐chloronaphthalene (volume content, 1.5%) was added into the solution half an hour before spin coating. FeCl_3_ was dissolved in chloroform (1.5 mg/ml) and added into PM6:Y6 solution before spin coating. All FeCl_3_ doping concentrations reported in this work are defined relative to the donor mass. The blend solution was spin coated on the ZnO coated ITO substrate at 2,000 rpm for 30 s, followed by annealing at 100°C for 10 min to form a 300 nm film. The 10 nm MoO_3_ film and the 100 nm Ag layer were sequentially deposited by thermal evaporation under a vacuum of 9 × 10^−4^ torr.

### 4.2 Fabrication of Conventional Devices

The POET‐T2 solution was dissolved in chlorobenzene (10 mg/ml) and stirred at 120°C overnight. The cleaned ITO substrates were pre‐heated on a hotplate, and hot POET‐T2 solution was spin coated on the top at 2000 rpm for 30 s, followed by thermal annealing at 220°C for 30 min for side chain cleavage. PM6:Y6 (1:1.2, 50 mg/ml in total), PM6:ITIC‐4F (1:1.2, 50 mg/ml), PTB7‐Th:SiOTIC‐4Cl (1:1.5, 75 mg/ml), and PTB7‐Th:COTIC‐4Cl (1:1.5, 75 mg/ml) were dissolved in chloroform. These blend solutions were spin coated on the substrates at 1000 rpm for 30 s followed by annealing at 100°C for 10 min to form 800 nm films. PFN‐Br (0.5 mg/ml in methanol) was spin coated at 2000 rpm for 30 s, and the 100 nm Ag layer was sequentially deposited by thermal evaporation under a vacuum of 9 × 10^−4^ torr.

### 4.3 *J–V* Characteristics and *EQE* Spectra

Dark *J–V* characteristics were recorded using a Keithley 2400 source measure unit. Every measurement data point is acquired after the steady‐state condition is achieved. *EQE* was calculated as the ratio of charge carriers generated by the device to the number of incident photons. The device was illuminated by a 100 W quartz tungsten halogen lamp, chopped at 192 Hz and passed through a monochromator (Princeton Instruments). The resulting photocurrent was amplified by a low‐noise current preamplifier (Stanford Research Systems SR570) and measured with a lock‐in amplifier (Stanford Research Systems SR830). An external bias was applied through the preamplifier, and the system was calibrated using standard Si and Ge detectors (Thorlabs).

### 4.4 Frequency‐Dependent Noise Spectra

To isolate the devices from ambient noise and electrical artifacts, all OPD noise measurements were conducted inside an electrically shielded dark box. The setup was optimized using a low‐noise amplifier with an internal bias supply and by minimizing the length of all connecting cables. Noise spectra were acquired by a signal analyzer (Agilent 35670A), which performed a fast Fourier transformation on the real‐time dark current. This current was first converted to a voltage by a low‐noise transimpedance amplifier (FEMTO DLCPA‐200). The sampling rate and number of data points were chosen to ensure high resolution at low frequencies and a clear trend at higher frequencies. With the measured EQE and noise spectral density (*i*_n_), the specific detectivity (*D* ^*^) can be calculated based on the equation:

$$\;D^{*}=\frac{q\lambda \mathrm{EQE}\sqrt{A}}{\mathrm{hc}i_{n}}$$

where *q* is the elementary charge, *λ* the incident wavelength, *A* the device area, *h* the Planck constant and *c* the speed of light.

### 4.5 Linear Dynamic Range

The light of a 910 nm LED (Thorlabs) driven by a function generator (Agilent 33220A) and modulated at 192 Hz was used to illuminate the sample. A series of light intensities were utilized by adjusting the voltage output of the function generator. Different light intensities were calibrated by standard Si and Ge detectors (Thorlabs). The photocurrent was first pre‐amplified (Stanford Research Systems SR570) and then recorded by a lock‐in amplifier (Stanford Research Systems SR830).

### 4.6 Transient Photocurrent and −3 dB Cutoff Frequency

Transient photocurrents were measured under illumination with an LED at 910 nm with a light intensity of 36 mW/cm^2^, driven by the function generator operated at 1 kHz. The generated photocurrent was pre‐amplified (Stanford Research Systems SR570) and fed to an oscilloscope (Tektronix MDO3014). Rise and fall times were calculated at 10% to 90% and 90% to 10% of the maximum photocurrent. −3 dB cutoff frequency was measured with the same LED light source and light intensity, driven by the function generator with a series of frequencies. The generated photocurrent of the investigated OPD was pre‐amplified (Stanford Research Systems SR570) and recorded by a lock‐in amplifier (Stanford Research Systems SR830).

### 4.7 Capacitance Characterization

Impedance spectroscopy was performed in an integrated platform PAIOS. Capacitance‐frequency and phase‐angle measurements were first performed to identify a stable capacitive regime, and the DC bias was set at −2 V. Capacitance‐voltage spectra were tested within the voltage range from −2 V to 2 V under dark conditions and a frequency of 1, 5, 10 kHz. The devices were shielded by aluminum foils in the dark environment.

### 4.8 Optical Simulation

The distribution of optical photon density and absorption profile was calculated using an open‐source software (OghmaNano). The wavelength‐dependent refractive indexes, *n*(λ), and extinction coefficients, *k*(λ) of the active layer were obtained by ellipsometry measurements.

## Author Contributions

J.Y., Y.Y., and F.S. conceived the project and designed the experimental work. Y.Y. and F.S. supervised the project. J.Y. fabricated the OPD devices and performed the major characterizations. V.M. performed the GIWAXS measurements and analysis under the supervision of H.A. Y.S. conducted the DFT simulations, and J.S. synthesized the POET‐T2 interlayer material under the supervision of W.Y. A.G. carried out the AFM measurements. J.Y. and Y.P. performed the noise and transient photocurrent measurements. J.Y. and A.G. performed the temperature‐dependent measurements under the supervision of K.G. X.D. carried out the contact angle measurements. J.M. performed the FTIR measurements under the supervision of A.A. R.H. performed the ellipsometry measurements under the supervision of B.C. J.Y. and Y.Y. wrote the original manuscript, which was reviewed and revised by K.G., B.C., A.A., H.A., W.Y., and F.S.

## Conflicts of Interest

The authors declare no conflicts of interest.

## Supporting information




**Supporting File**: adma75009‐sup‐0001‐SuppMat.pdf.

## Data Availability

All data are available in the main text and the supporting information from the corresponding authors upon reasonable request.

## References

[1] J. Huang , J. Lee , J. Vollbrecht , et al., “A High‐Performance Solution‐Processed Organic Photodetector for Near‐Infrared Sensing,” Advanced Materials 32, no. 1 (2020): 1906027, 10.1002/adma.201906027. 31714629

[2] S. Tong , J. Yuan , C. Zhang , et al., “Large‐Scale Roll‐to‐roll Printed, Flexible and Stable Organic Bulk Heterojunction Photodetector,” npj Flexible Electronics 2, no. 1 (2018): 7, 10.1038/s41528-017-0020-y.

[3] J. Wang , S. Wei , N. Qin , and T. H. Tao , “An Insect‐Scale Artificial Visual‐Olfactory Bionic Compound Eye,” Nature Communications 17, no. 1 (2026): 2259, 10.1038/s41467-026-68940-0. PMC12966333 41629320

[4] Y. Chen , Y. Zheng , J. Wang , et al., “Ultranarrow‐Bandgap Small‐Molecule Acceptor Enables Sensitive SWIR Detection and Dynamic Upconversion Imaging,” Science Advances 10, no. 23 (2024): adm9631, https://www.science.org/doi/abs/10.1126/sciadv.adm9631. 10.1126/sciadv.adm9631 PMC11152131 38838154

[5] S. Talanti , K. Fu , X. Zheng , et al., “CMOS‐Integrated Organic Neuromorphic Imagers for High‐Resolution Dual‐Modal Imaging,” Nature Communications 16, no. 1 (2025): 4311, 10.1038/s41467-025-59446-2. PMC12062424 40340983

[6] D. Baierl , L. Pancheri , M. Schmidt , et al., “A Hybrid CMOS‐Imager With a Solution‐Processable Polymer as Photoactive Layer,” Nature Communications 3, no. 1 (2012): 1175, 10.1038/ncomms2180. 23132025

[7] S.‐J. Lim , D.‐S. Leem , K.‐B. Park , et al., “Organic‐on‐Silicon Complementary Metal–Oxide–Semiconductor Colour Image Sensors,” Scientific Reports 5, no. 1 (2015): 7708, 10.1038/srep07708. PMC4289892 25578322

[8] H. Shekhar , A. Fenigstein , T. Leitner , B. Lavi , D. Veinger , and N. Tessler , “Hybrid Image Sensor of Small Molecule Organic Photodiode on CMOS—Integration and Characterization,” Scientific Reports 10, no. 1 (2020): 7594, 10.1038/s41598-020-64565-5. PMC7200686 32372047

[9] A. J. P. Theuwissen , “CMOS Image Sensors: State‐of‐the‐Art,” Solid‐State Electronics 52, no. 9 (2008): 1401–1406, 10.1016/j.sse.2008.04.012.

[10] S. Yokogawa , S. P. Burgos , and H. A. Atwater , “Plasmonic Color Filters for CMOS Image Sensor Applications,” Nano Letters 12, no. 8 (2012): 4349–4354, 10.1021/nl302110z. 22799751

[11] M. Yako , Y. Yamaoka , T. Kiyohara , et al., “Video‐Rate Hyperspectral Camera Based on a CMOS‐Compatible Random Array of Fabry–Pérot Filters,” Nature Photonics 17, no. 3 (2023): 218–223, 10.1038/s41566-022-01141-5.

[12] J. H. Kim , A. Liess , M. Stolte , et al., “An Efficient Narrowband Near‐Infrared at 1040 Nm Organic Photodetector Realized by Intermolecular Charge Transfer Mediated Coupling Based on a Squaraine Dye,” Advanced Materials 33, no. 26 (2021): 2100582, 10.1002/adma.202100582. PMC11469076 34060157

[13] J. H. Kim , T. Schembri , D. Bialas , M. Stolte , and F. Würthner , “Slip‐Stacked J‐Aggregate Materials for Organic Solar Cells and Photodetectors,” Advanced Materials 34, no. 22 (2022): 2104678, 10.1002/adma.202104678. 34668248

[14] S. Xing , E. Bittrich , V. Prifti , et al., “Light‐Induced Fine‐Tuning of Optical Cavities for Organic Optoelectronic Devices,” Nature Communications 16, no. 1 (2025): 8426, 10.1038/s41467-025-64272-7. PMC12462449 40998815

[15] B. Siegmund , A. Mischok , J. Benduhn , et al., “Organic Narrowband Near‐Infrared Photodetectors Based on Intermolecular Charge‐Transfer Absorption,” Nature Communications 8, no. 1 (2017): 15421, 10.1038/ncomms15421. PMC5465315 28580934

[16] Y. Tang , Z. Wang , M. Gao , J. Han , L. Yuan , and F. Zhu , “Dual‐Mode Narrowband Organic Photodetectors for Self‐Aligned Imaging in NIR‐I and NIR‐II,” Nature Communications 16, no. 1 (2025): 7144, 10.1038/s41467-025-62394-6. PMC12322033 40759990

[17] B. Xie , R. Xie , K. Zhang , et al., “Self‐Filtering Narrowband High Performance Organic Photodetectors Enabled by Manipulating Localized Frenkel Exciton Dissociation,” Nature Communications 11, no. 1 (2020): 2871, 10.1038/s41467-020-16675-x. PMC7280211 32514001

[18] A. Armin , R. D. Jansen‐van Vuuren , N. Kopidakis , P. L. Burn , and P. Meredith , “Narrowband Light Detection via Internal Quantum Efficiency Manipulation of Organic Photodiodes,” Nature Communications 6, no. 1 (2015): 6343, 10.1038/ncomms7343. 25721323

[19] Q. Liu , S. Zeiske , X. Jiang , et al., “Electron‐Donating Amine‐Interlayer Induced n‐Type Doping of Polymer:Nonfullerene Blends for Efficient Narrowband Near‐infrared Photo‐Detection,” Nature Communications 13, no. 1 (2022): 5194, 10.1038/s41467-022-32845-5. PMC9440933 36057674

[20] T. H. Kim , S. Y. Jeong , S. Oh , et al., “Control of Chemical Doping‐Mediated Space Charge for Energetic Band‐Switching Modulation in Low‐Noise Shortwave‐Infrared Organic Photodetector,” Advanced Materials 37, no. 30 (2025): 2500126, 10.1002/adma.202500126. 40346779

[21] L. Chi Yat , P. K. T. Mok , and L. Ka Nang , “A 1‐V Integrated Current‐Mode Boost Converter in Standard 3.3/5‐V CMOS Technologies,” IEEE Journal of Solid‐State Circuits 40, no. 11 (2005): 2265, 10.1109/JSSC.2005.857374.

[22] V. Suntharalingam , R. Berger , J. A. Burns , et al., “Megapixel CMOS image sensor fabricated in three‐dimentional integrated circuit technology,” presented at *ISSCC. 2005 IEEE International Digest of Technical Papers. Solid‐State Circuits Conference* , vol. 1 (2005): 356.

[23] S. Hamami , L. Fleshel , and O. Yadid‐Pecht , “CMOS Image Sensor Employing 3.3 V 12bit 6.3MS/s Pipelined ADC,” Sensors and Actuators A: Physical 135, no. 1 (2007): 119–125, 10.1016/j.sna.2006.06.041.

[24] A. Theuwissen , “Chapter 7‐CMOS Image Sensors,” in Smart Sensor Systems, ed. G. Meijer , M. Pertijs , and K. Makinwa. , (John Wiley & Sons, Ltd., 2014), ISBN 978‐0‐470‐68600‐3.

[25] X. Yu , H. Lin , Z. He , et al., “High‐Performance Near‐Infrared Organic Photodetectors Enabled by Fabricating an Interdigital Heterojunction,” Advanced Optical Materials 10 (2024): 2302170, 10.1002/adom.202302170.

[26] H. T. Chandran , R. Ma , Z. Xu , et al., “High‐Detectivity All‐Polymer Photodiode Empowers Smart Vitality Surveillance and Computational Imaging Rivaling Silicon Diodes,” Advanced Materials 36, no. 38 (2024): 2407271, 10.1002/adma.202407271. 39081083

[27] Y. Song , Z. Zhong , P. He , et al., “Doping Compensation Enables High‐Detectivity Infrared Organic Photodiodes for Image Sensing,” Advanced Materials 34, no. 29 (2022): 2201827, 10.1002/adma.202201827. 35561337

[28] Y. Wei , H. Chen , T. Liu , et al., “Self‐Powered Organic Photodetectors With High Detectivity for Near Infrared Light Detection Enabled by Dark Current Reduction,” Advanced Functional Materials 31, no. 52 (2021): 2106326, 10.1002/adfm.202106326.

[29] J. Yi , Y. Yang , Y. Pei , et al., “Trap‐Modified Inverted Organic Photodetectors via Layer‐by‐Layer Processing With Poly(N‐vinylcarbazole) Additives,” Advanced Functional Materials 36, no. 28 (2025): 19588, 10.1002/adfm.202519588.

[30] G. Simone , M. J. Dyson , C. H. L. Weijtens , et al., “On the Origin of Dark Current in Organic Photodiodes,” Advanced Optical Materials 8, no. 1 (2020): 1901568, 10.1002/adom.201901568.

[31] G. Simone , M. J. Dyson , S. C. J. Meskers , R. A. J. Janssen , and G. H. Gelinck , “Organic Photodetectors and Their Application in Large Area and Flexible Image Sensors: The Role of Dark Current,” Advanced Functional Materials 30, no. 20 (2020): 1904205, 10.1002/adfm.201904205.

[32] Z. Liang , H. H. Choi , X. Luo , et al., “n‐Type Charge Transport in Heavily p‐Doped Polymers,” Nature Materials 20, no. 4 (2021): 518–524, 10.1038/s41563-020-00859-3. 33398117

[33] S. A. Gregory , R. Hanus , A. Atassi , et al., “Quantifying Charge Carrier Localization in Chemically Doped Semiconducting Polymers,” Nature Materials 20, no. 10 (2021): 1414–1421, 10.1038/s41563-021-01008-0. 34017120

[34] V. A. Trukhanov , V. V. Bruevich , and D. Y. Paraschuk , “Effect of Doping on Performance of Organic Solar Cells,” Physical Review B 84, no. 20 (2011): 205318, https://link.aps.org/doi/10.1103/PhysRevB.84.205318.

[35] A. Wagenpfahl , D. Rauh , M. Binder , C. Deibel , and V. Dyakonov , “S‐Shaped Current‐Voltage Characteristics of Organic Solar Devices,” Physical Review B 82, no. 11 (2010): 115306, https://link.aps.org/doi/10.1103/PhysRevB.82.115306.

[36] J. Shanahan , L. Yan , Y. Olanrewaju , et al., “Acid‐Triggered Side Chain Cleavage Leads to Doped Conjugated Polymers of High Conductivity,” Journal of the American Chemical Society 146, no. 47 (2024): 32243–32248, 10.1021/jacs.4c09843. 39466715

