# Supplementary material for: Spectral Selective and Low‐Noise Thin‐Film Organic Photodetectors Enabled by Electric‐Field Engineering
Source: Adv Mater. 2026 Sep 15;38(61):e75009. doi: 10.1002/adma.75009 (PMC13726700; doi:10.1002/adma.75009)
Supplement: Supplementary file 1 [file ADMA-38-e75009-s001.pdf]

Supporting Information

Spectral selective and low-noise thin-film organic photodetectors enabled by electric-field engineering

Jingwei Yi, Yi Yang, Vahid Mahdikhah, Yibo Shi, Jordan Shanahan, Apurva Gadikar, Yusen Pei, Xinyun Dong, Jacob P Mauthe, Reece Henry, Kenan Gundogdu, Brendan T O'Connor, Aram Amassian, Harald Ade, Wei You, Franky So**

Jingwei Yi, Yi Yang, Apurva Gadikar, Yusen Pei, Jacob P Mauthe, Aram Amassian, Franky So
Department of Materials Science and Engineering
North Carolina State University
Raleigh, NC 27695, USA
E-mail: yyang98@ncsu.edu; fso@ncsu.edu

Vahid Mahdikhah
Department of Electrical and Computer Engineering
North Carolina State University
Raleigh, NC 27695, USA

Yibo Shi, Jordan Shanahan, Wei You
Department of Chemistry
University of North Carolina at Chapel Hill
Chapel Hill, NC 27599, USA

Xinyun Dong, Kenan Gundogdu, Harald Ade
Department of Physics
North Carolina State University
Raleigh, NC 27695, USA

Reece Henry, Brendan T O'Connor
Department of Mechanical and Aerospace Engineering
North Carolina State University
Raleigh, NC 27695, USA

Jingwei Yi, Yi Yang, Vahid Mahdikhah, Apurva Gadikar, Yusen Pei, Xinyun Dong, Jacob P Mauthe, Reece Henry, Kenan Gundogdu, Brendan T O'Connor, Aram Amassian, Harald Ade, Wei You, Franky So
Organic and Carbon Electronics Laboratories (ORaCEL)
North Carolina State University
Raleigh, NC 27695, USA

Additional experimental section

Materials: poly[(2,6-(4,8-bis(5-(2-ethylhexyl)-4-fluoro)thiophen-2-yl)-benzo[1,2-b:4,5-b']dithiophene))-alt-(5,5-(1\',3\'-di-2-thienyl-5\',7\'-bis(2-ethylhexyl)benzo[1\',2\'-c:4\',5\'-c\']dithiophene-4,8-dione))] (PM6), 2,2'-((2Z,2'Z)-((12,13-bis(2-ethylhexyl)-3,9-diundecyl-12,13-dihydro-thiadiazolo[3,4-e]thieno[2\",3′:4',5']thieno[2',3':4,5]pyrrolo[3,2-g]thieno[2',3':4,5]thieno[3,2-b]indole-2,10-diyl)bis(methanylylidene))bis(5,6-difluoro-3-oxo-2,3-dihydro-1H-indene-2,1-diylidene))dimalononitrile (Y6), poly([2,6′-4,8-di(5-ethylhexylthienyl)benzo[1,2-b;3,3-b]dithiophene]{3-fluoro-2[(2-ethylhexyl)carbonyl]thieno[3,4-b]thiophenediyl}) (PTB7-Th), bis-2,2'-[[4,4-bis(2-ethylhexyl)-4H-silolo[3,2-b:4,5-b']dithiophene-2,6- diyl]bis[[4-[(2-ethylhexyl)oxy]-5,2-thiophenediyl]-(Z)-methylidyne(5,6-dichloro-3-oxo-1H-indene-2,1(3H)-diylidene)]]- Propanedinitrile (SiOTIC-4Cl), 2,2'-((2Z,2'Z)-(((4,4-bis(2-ethylhexyl)-4H-cyclopenta[2,1-b:3,4-b']dithiophene-2,6-diyl)bis(4-(2-ethylhexyloxy)thiophene-5,2-diyl))bis(methanylylidene))bis(5,6-dichloro-3-oxo-2,3-dihydro-1H-indene-2,1-diylidene))dimalononitrile (COTIC-4Cl), 3,9-bis(2-methylene-((3-(1,1-dicyanomethylene)-6,7-difluoro)-indanone))-5,5,11,11-tetrakis(4-hexylphenyl)-dithieno[2,3-d:2',3'-d']-s-indaceno[1,2-b:5,6-b']dithiophene (ITIC-4F) and poly(9,9-bis(3'-(*N*,*N*-dimethyl)-*N*-ethylammoinium-propyl-2,7-fluorene)-alt-2,7-(9,9-dioctylfluorene))dibromide (PFN-Br) were purchased from 1-Materials. POET-T2 was synthesized in-house. Iron chloride ($FeCl_3$, ≥99.8%) and tricyclohexylphosphine ($P(Cy)_3$, ≥99.8%) were purchased from Sigma Aldrich. 2,3,5,6-tetrafluoro-7,7,8,8-tetracyanoquinodimethane (F_4TCNQ, ≥99.8%) was purchased from e-Ray Optoelectronics Technology. 2,5-difluoro-7,7,8,8-tetracyanoquinodimethane (F_2TCNQ, ≥99%) was purchased from TCI Chemicals. Chloroform and other solvents used in this work were purchased from Sigma Aldrich and used as received. Poly(3,4-ethylenedioxythiophene) polystyrene sulfonate (PEDOT:PSS) (CLEVIOS PVP Al 4083) was purchased from Heraeus. For zinc oxide (ZnO) sol gel solution, zinc acetate ($Zn(CH_3COO)_2{\cdot}2H_2O$), 2-methoxyethanol and ethanolamine were purchased from Sigma-Aldrich. All air and moisture sensitive compounds were stored and handled under an inert atmosphere in a glovebox.

Additional device fabrication: For comparison with POET-T2-COOH interlayer, we fabricated PEDOT:PSS-based PM6:Y6 conventional devices with the structure of ITO/PEDOT:PSS/PM6:Y6+0.2% $FeCl_3$/PFN-Br/Ag. PEDOT:PSS was spin coated at 4000 rpm for 30 s outside the glovebox and annealed at 150°C for 15 min. For PM6:Y6 inverted narrowband

OPDs, $P(Cy)_3$ was dissolved in chloroform (15 mg/ml) and added into PM6:Y6 solution (1:1.2, 50 mg/ml in total) before spin coating. The sol-gel ZnO precursor was prepared by dissolving 0.4 g zinc acetate in 4 mL 2-methoxyethanol with 110 mL ethanolamine and then stirred at 75°C for 4 h.

UV-vis-NIR absorption spectra: UV-vis-NIR absorption spectra were recorded from 350-1200 nm on spectrophotometer (PerkinElmer Lambda 750). Active layer thin films with the same thickness as devices were prepared by spin coating onto glass substrates. Film thickness was recorded on a profilometer (Bruker Dektak XT).

Fourier transform infrared (FTIR) spectroscopy: Thin films were deposited on double-side polished (DSP) silicon substrates for absorption-mode FTIR measurements. Spectra were collected using a Bruker VERTEX 70 equipped with the standard MIR source, KBr beamsplitter, and room temperature DLaTGS detector at 4 cm-1 spectral resolution. Background correction data was taken using a bare DSP silicon substrate. Each film was then centered on the optical path for measurement. Data were acquired in the 600-4000 cm^{-1} range at 4 cm^{-1} resolution and averaged over 128 scans.

High-resolution XPS spectra: High-resolution XPS measurements were performed with 10-14 kV for Al/Mg and Al/Ag sources with a PHOIBOS 150 analyzer (<1 eV resolution). To overcome the influence in binding energy from sample charging during the measurements, the XPS spectra were calibrated to the C*1s* peak(C-C/C=C), assuming it at a constant position of 284.8 eV.

Grazing incidence wide-angle X-ray scattering (GIWAXS) measurements: GIWAXS measurements were performed at Beamline 7.3.3 of the Advanced Light Source at Berkeley National Laboratory using a Pilatus 2 M area detector. Data were collected at an incident angle of 0.13° and processed with Nika and WAXS tools in Igor Pro. 1D GIWAXS profiles were analyzed using the Multipeak Fitting package.

Atomic force microscopy (AFM) measurements: Tapping-mode AFM images of active layer films were obtained using the MFP-3D-BIO commercial instrument by Asylum Research.

Contact angle measurements: PEDOT:PSS and POET-T2-COOH films were prepared on glass substrates. A drop-shape analyzer (Ossila DSA-100) was used to drop different solvents and measure the sessile-drop contact angles at room temperature.

Ellipsometry measurement: VASE measurements were taken using a J.A. Woollam M-2000 ellipsometer using collection angles of 55°, 60°, 65°, 70° and 75° and in the spectral range of 192

nm to 1620 nm. The beam diameter was approximately 3mm. Modeling of the ellipsometric data was done in the software CompleteEASE, developed by the J.A. Woollam company. The model stack used consisted of a silicon substrate, a silicon oxide layer of thickness 1.5 nm and a biaxial general oscillator model utilizing Gaussian peak shapes. The general oscillator model was determined by first fitting the ellipsometric data to a B-spline fit in order to determine the general shape of the optical spectrum from the raw ellipsometric data. The B-spline model was then swapped to a general oscillator model with manually placed peaks and refined to minimize mean-squared-error.

Density functional theory (DFT) simulations: Molecular energy level and electrostatic potentials were determined by DFT simulations by the GAUSSAIN16 software. All simulations were performed on single molecules in the gas phase using B3LYP level of theory and basis set 6-31G(d.p).

Note S1. Definition for the analysis in GIWAXS

To quantify the lamellar (100) and π-π stacking (010) structures, peak positions and widths in the GIWAXS profiles were analyzed to extract the *d*-spacing, coherence length, and paracrystalline disorder.

1. *Coherence length (L_c)*

   The crystalline coherence length, representing the average domain size perpendicular to the (hkl) planes, was estimated using the Scherrer equation in q-space:

$$L_c = \frac{2\pi \mathrm{K}}{\Delta q_{\mathrm{hkl}}}$$

   where K=0.9 (Scherrer constant), λ = X-ray wavelength, Δq = full width at half maximum (FWHM) in q-space.

2. *d-spacing (d_{hkl})*

   The interplanar spacing was calculated from the peak position using:

$$d_{\mathrm{hkl}} = \frac{2\pi}{q_{\mathrm{hkl}}}$$

   which corresponds to the distance between stacked planes, such as lamellar or π-π stacking.

3. *Paracrystalline disorder (g)*

   The paracrystalline disorder parameter (g), describing lattice distortion and stacking disorder, was estimated as:

$$g = \frac{1}{2\pi}\sqrt{\Delta q_{\mathrm{hkl}} \times d_{\mathrm{hkl}}}$$

   where a larger (g) value indicates greater disorder in the stacking periodicity.

Note S2. Mott-Schottky analysis

The internal electric-field distribution in organic photodiodes was probed using capacitance-voltage (*C*-*V*) measurements based on Mott-Schottky analysis. Under reverse bias, the device can be approximated as a capacitor, where the measured capacitance is primarily determined by the thickness of the region in which the electric field is present. As the applied voltage changes, the extent of this region varies, resulting in a voltage-dependent capacitance.

For a uniformly doped semiconductor, the Mott-Schottky relation is given by:

$$\frac{1}{C^2}=\frac{2(V_{\mathrm{bi}}-V)}{q\varepsilon_{\mathrm{r}}\varepsilon_0 N}$$

where C is the capacitance, V is the applied voltage, V_{bi} is the built in potential, q is the elementary charge, ε_0 is the vacuum permittivity, ε_{r} is the device permittivity, and N is the carrier concentration. A linear dependence of $1/C^2$ versus V allows the extraction of the effective carrier concentration from the slope.

The capacitance is related to the width of the field-dominated region (W) through:

$$C=\frac{\varepsilon_{\mathrm{r}}\varepsilon_0 A}{W}$$

where A is the device area (0.04 cm^2). Therefore, the extracted capacitance provides a direct measure of the spatial extent of the region where the electric field is non-zero. In this work, W is interpreted as the effective high-field region within the active layer.

It should be noted that Mott-Schottky analysis is originally developed for inorganic semiconductors with well-defined depletion regions. In contrast, organic bulk heterojunction (BHJ) systems consist of interpenetrating donor-acceptor phases with energetic disorder and do not form an ideal *p*-*n* junction. Consequently, parameters such as carrier concentration and depletion width obtained from Mott-Schottky analysis should be regarded as effective quantities.

Despite these differences, under reverse bias, BHJ photodiodes with asymmetric contacts can still exhibit a depletion-like region governed by the internal electric-field distribution. The built-

in potential, together with the redistribution of mobile carriers, leads to a spatially non-uniform electric field. In this context, the measured capacitance predominantly reflects the thickness of the region where the electric field is present, analogous to the space-charge region in conventional semiconductors. Importantly, in this work, Mott-Schottky analysis is used to track the relative evolution of the internal electric-field distribution upon chemical doping. The approximately linear $1/C^2$ versus V behavior observed across different devices supports the validity of this effective description. Upon $FeCl_3$ incorporation, the extracted carrier concentration increases significantly, accompanied by a pronounced reduction in the effective high-field region width. This trend is consistent with the expected screening effect induced by dopant-generated carriers, which redistribute under the built-in field and partially neutralize the electric field. As a result, a field-free region forms near one contact, while the high-field region becomes confined.

Therefore, although Mott-Schottky analysis in BHJ systems should be interpreted with caution, the systematic variation observed here provides reliable evidence for doping-induced electric-field redistribution. The extracted high-field region should thus be understood as an effective parameter describing the spatial extent of field-assisted carrier collection, rather than a strict depletion width in the classical sense.

Note S3. Drift-diffusion simulation

The energy band diagram was simulated in drift-diffusion model. The distribution of free carriers and electrostatic potential was obtained by solving Poisson's equation:[1]

$$\frac{dE(x)}{dx}=\frac{\rho(x)}{\varepsilon_r}$$

where $E(x)$ is the electric field, ε_r is the dielectric constant, and $\rho(x)$ is the local charge density given by:

$$\rho(x)=q[p(x) - n(x) + N_D^+(x) - N_A^-(x)]$$

where q is the elementary charge, $n(x)$ and $p(x)$ are the electron and hole concentrations, respectively, and $N_D^+(x)$ and $N_A^-(x)$ represent ionized donor and acceptor charges. Carrier transport was described by the drift-diffusion equations:

$$J_n = qn\mu_n E + \mu_n kT\frac{dn}{dx}$$

$$J_p = qn\mu_p E - \mu_p kT\frac{dp}{dx}$$

where J_n and J_p are the electron and hole current densities, μ_n and μ_p are the carrier mobilities. The carrier concentration needs to satisfy the continuity equations:

$$\frac{1}{q}\frac{dJ_n}{dx}=G - R$$

$$-\frac{1}{q}\frac{dJ_p}{dx}=G - R$$

where G and R are the carrier generation and recombination rates, respectively. The active-layer thickness, energy levels, contact work functions, and dielectric constant were listed below. The carrier concentrations used in the simulations were obtained from Mott-Schottky analysis, while the electron and hole mobilities were extracted from electron-only and hole-only SCLC measurements. Simulations were performed for both dark and illuminated conditions. For

illuminated simulations, the optical generation profile was calculated from transfer-matrix-method (TMM) simulations using the experimentally measured refractive index and extinction index.

Parameter	Symbol	Numerical value
Active layer thickness	L	300/800 nm
Temperature	T	300 K
Anode work function	φ_a	5.3 eV
Cathode work function	φ_c	4.3 eV
Carrier concentration	$N_{h,p}$	Mott-Schottky
Carrier mobility	$\mu_{h,e}$	SCLC
Dielectric constant	ε_r	3.5
Effective density of states	$N_{c,v}$	10^{26} m^{-3}
Electron-hole pair distance	a_0	1.3 nm
Decay rate	k_f	10^4 s^{-1}

Note S4. Device optimization for dark current analysis

The origin of dark current density (J_d) under reverse bias is critical for understanding device performance. Multiple mechanisms can contribute to J_d, including shunt leakage, thermal generation, and carrier injection at the contacts. In this section, we systematically evaluate these possible contributions and identify the dominant origin of dark current in PM6:Y6 devices.

1. *Shunt leakage and thickness dependence*

Shunt leakage in organic photodiodes is typically associated with unintended ohmic conduction pathways directly connecting the two electrodes. It can also more generally refer to any alternative low-resistance transport channel that bypasses the rectifying junction.[2] In the present device architecture, where the several hundred nanometer of bulk heterojunction is sandwiched between well-defined electron- and hole-selective interlayers, the formation of such non-rectifying transport paths within the organic stack is expected to be strongly suppressed.[3]

To evaluate the contribution of shunt leakage, the thickness of the active layer and interlayers was systematically varied. As shown in Figure S15a-c, increasing the thickness of the active layer (100/300/500 nm), MoO_3 (0/5/10/30 nm) and ZnO (0/10/20/50) does not lead to a continuous reduction in J_d. Furthermore, no severe dependence of J_d on active layer thickness is observed. Since shunt-dominated devices typically exhibit reduced leakage current with increasing thickness, the absence of such behavior indicates that shunt leakage is not the dominant origin of dark current in these devices.

2. *Thermal generation*

Thermal generation arises from the intrinsic excitation of charge carriers across the effective bandgap of the semiconductor, leading to an almost bias-independent contribution to the dark current.[4] In organic bulk heterojunctions, this process is typically governed by the density of states and the energetic gap between the donor and acceptor materials. As a result, thermally generated current is expected to exhibit a weak dependence on the applied electric field and a strong temperature dependence following Arrhenius behavior $J_0 \propto \exp(-E_a/kT)$, with an activation energy (E_a) on the order of the effective bandgap or approximately half of it.[5] Therefore, analysis

of the temperature dependence of J_d provides a direct means to distinguish thermal generation from injection-driven processes.

For a relatively wide bandgap material system PM6:Y6 (E_g>1eV), the expected dark saturation current is in the order of ~10^{-7} mA/cm^{-2}, which is several orders of magnitude lower than the measured J_d under reverse bias.[6] This discrepancy excludes thermal generation as the dominant contribution. Temperature-dependent *J-V* measurements (Figure S15d-e) further show that the extracted activation energy decreases from 0.19 eV (-0.1 V) to 0.16 eV (-2 V), which is significantly smaller than the effective bandgap or half-bandgap. Such low E_a values are inconsistent with thermally activated generation but are characteristic of field-assisted injection processes.

3. Contact selectivity and interfacial injection

Injection-driven dark current originates from the carrier injection at the electrode-organic interfaces under an applied electric field.[7] In organic photodetectors, this process is typically governed by the energy level alignment between the electrodes and the molecular orbitals of the active layer, where electrons are injected into the tail states of the acceptor LUMO and holes into the donor HOMO. Once injected, carriers traverse the bulk heterojunction through field-assisted hopping transport, contributing to the measured dark current.

The device structure used in this work is ITO/ZnO/PM6:Y6/MoO_3/Ag. To evaluate the role of interfacial injection, the injection barriers were selectively tuned by modifying the transport layers. Specifically, MoO_3 was replaced with the higher work-function interlayer HAT-CN to suppress electron injection, while ZnO was replaced with the lower work-function interlayer PEIE to suppress hole injection. As shown in Figure S15f, a pronounced reduction in J_d is observed only when MoO_3 is replaced, whereas modifying the ZnO side has negligible effect. These results indicate that electron injection from the MoO_3/Ag interface is the dominant injection pathway contributing to dark current.

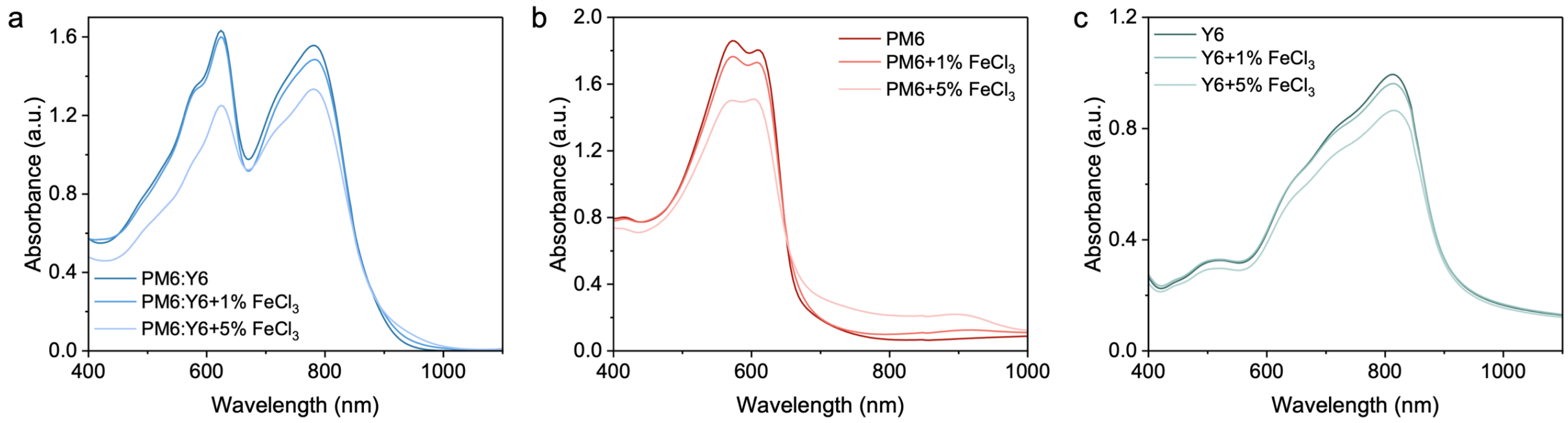

Fig. S1. Comparison of absorption spectra after doping. Measured absorption spectra of (**a**) PM6:Y6 blend, (**b**) neat PM6, and (**c**) neat Y6 on glass substrates at different $FeCl_3$ dopant concentrations. Upon incorporation of $FeCl_3$ (1 wt% and 5 wt%), a pronounced bleaching of the π-π* absorption peaks is observed in all systems, indicating effective charge-transfer interactions between the dopant and both donor and acceptor components. In the PM6:Y6 blend and neat PM6 films, an additional broad absorption band emerges in the near-infrared region upon doping, with its intensity increasing with $FeCl_3$ concentration. This feature is attributed to polaronic absorption associated with dopant-induced charge carriers in the conjugated polymer. In contrast, such a distinct NIR polaron band is not clearly resolved in the Y6 films under similar doping conditions. This difference may arise from variations in polaron absorption strength, spectral overlap, or the nature of charge localization in the acceptor, suggesting that the optical signature of doping is more pronounced in the PM6 component.

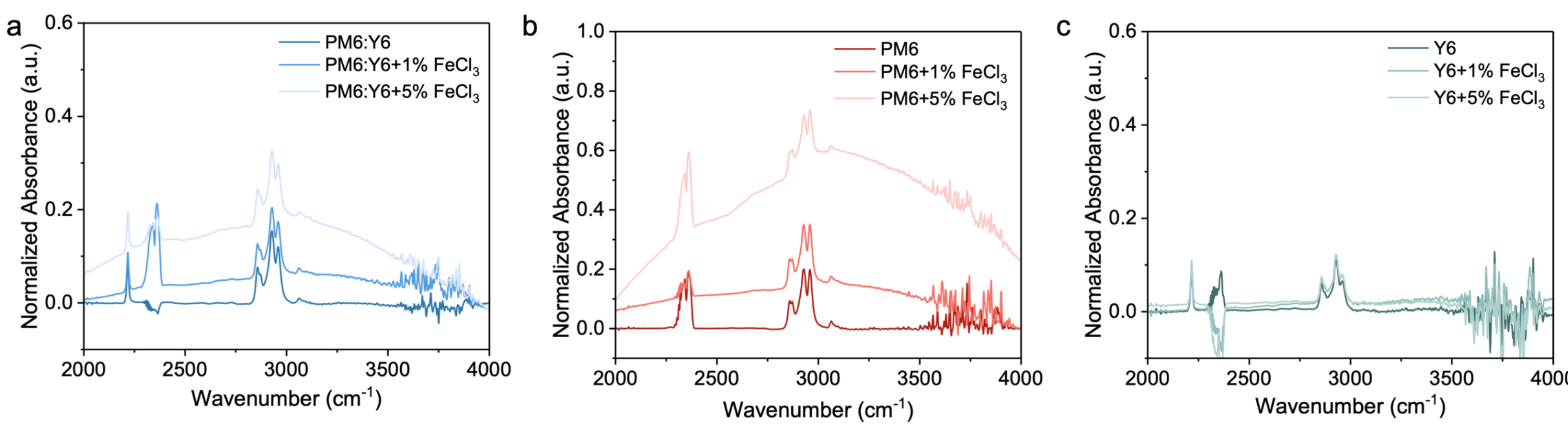

Fig. S2. Comparison of FTIR spectra after doping. Measured FTIR spectra of (**a**) PM6:Y6 blend, (**b**) neat PM6, and (**c**) neat Y6 on double-sliced silicon substrates after different $FeCl_3$ dopant concentrations. Spectra are normalized to the layer thickness. Upon $FeCl_3$ incorporation, a broad absorption feature emerges in the mid-infrared region (~2000-4000 cm^{-1}) for both the PM6:Y6 blend and neat PM6 films, with its intensity increasing with dopant concentration. This broad band is commonly associated with polaronic absorption arising from intragap states induced by charge-transfer doping in conjugated systems. We note that the higher $FeCl_3$ concentrations used for selected spectroscopic measurements were chosen to enhance the weak doping-induced signatures and are used to identify the electronic interactions associated with doping rather than to quantitatively reproduce the optimized device condition.

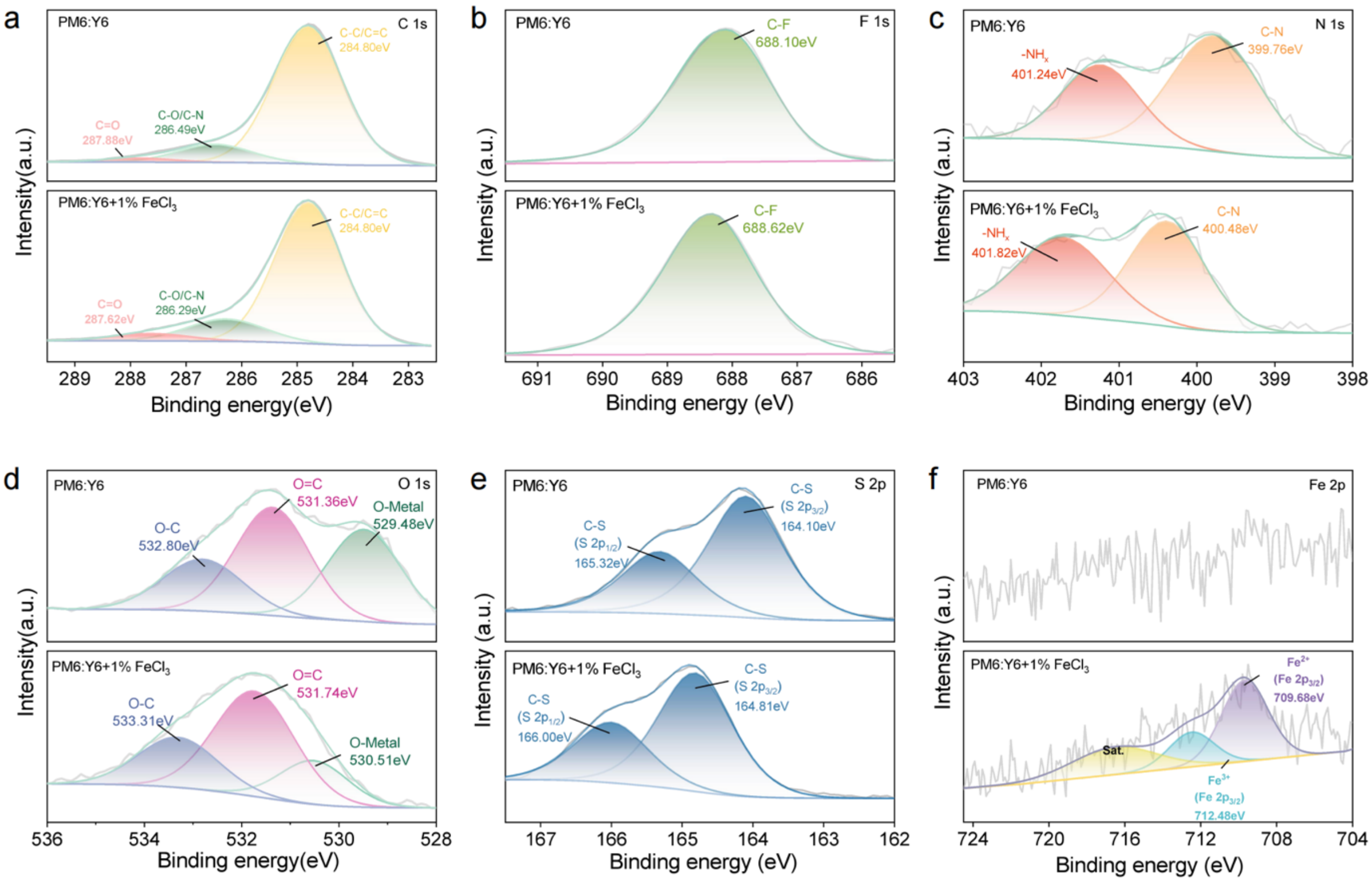

Fig. S3. Comparison of XPS spectra after doping. High-resolution X-ray photoelectron spectroscopy spectra of pristine and 1 wt% $FeCl_3$-doped PM6:Y6 films after Shirley background subtraction: (**a**) C*1s*, (**b**) F*1s*, (**c**) N*1s*, (**d**) O*1s*, (**e**) S*2p*, and (**f**) Fe*2p*. To accurately compare the binding energy shift after doping, the C-C/C=C peak was calibrated in 284.80 eV position. All core elements shift systematically toward higher binding energies after doping, suggesting that the electronic perturbation is not localized to a specific functional group but instead extends across the broader conjugated framework. In particular, the shift of the *N*1s signal, for which N is present in Y6 but not PM6, indicates that the electronic influence of $FeCl_3$ also involves the Y6 phase but not only limited in PM6 phase. For the undoped PM6:Y6 film, Fe*2p* signal is completely from background noise. After 1 wt% $FeCl_3$ incorporation, Fe*2p* signal can be identified. However, as the Fe atom concentration is extremely low, the signal intensity is not as strong as other atom elements presented in this work.

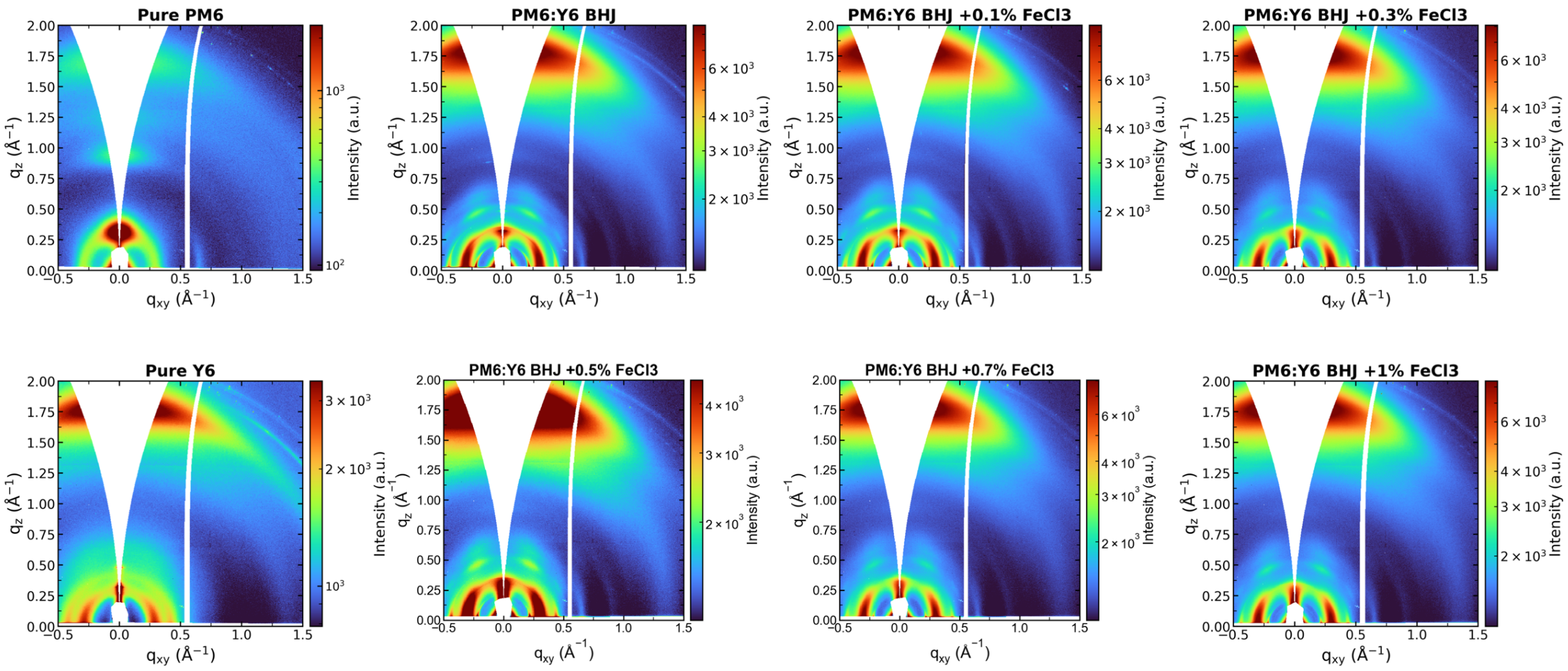

Fig. S4. Two-dimensional (2D) patterns of grazing incidence wide-angle X-ray scattering (GIWAXS).

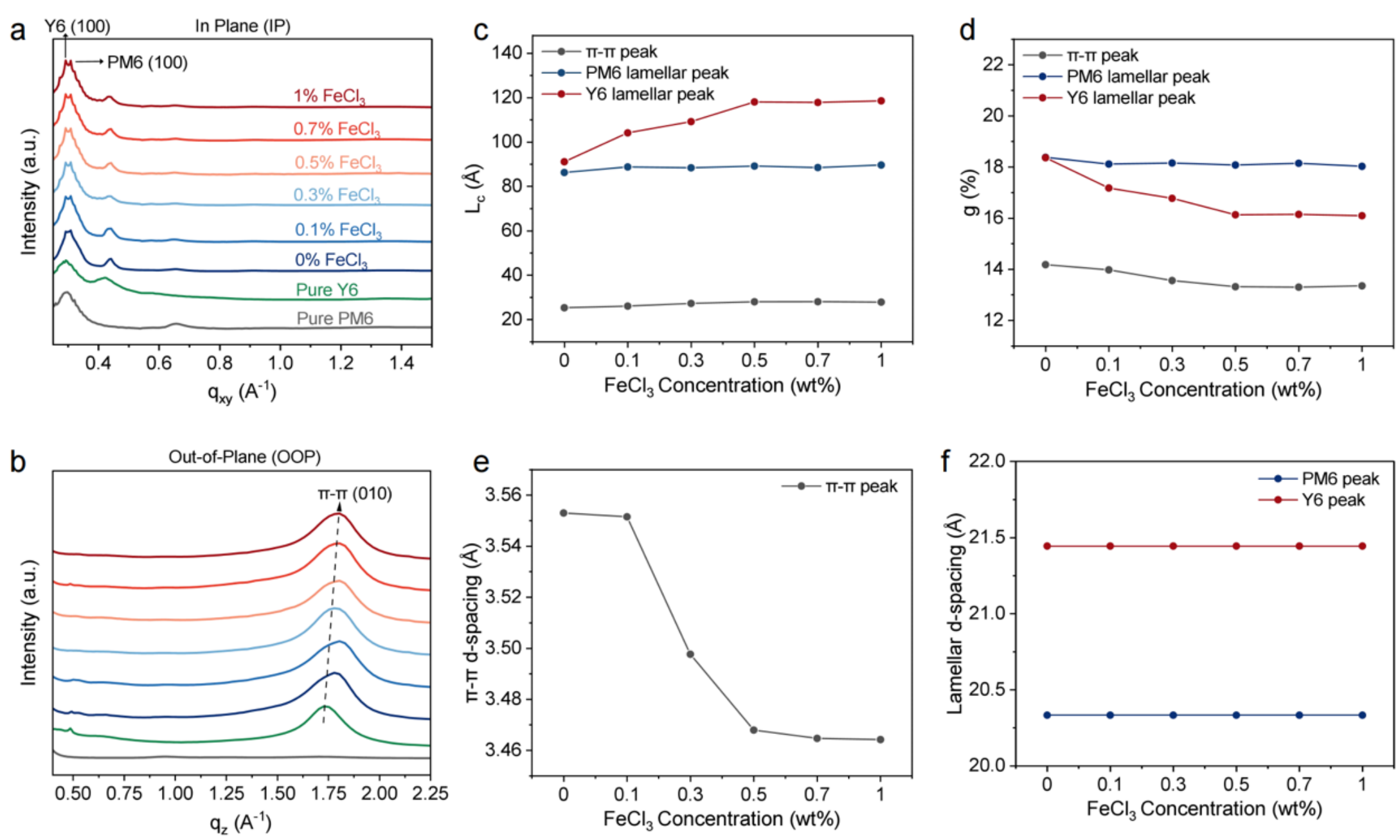

Fig. S5. Evolution of molecular packing. Grazing-incidence wide-angle X-ray scattering (GIWAXS) profiles of PM6:Y6 films with varying $FeCl_3$ concentrations in the (**a**) in-plane (IP) and (**b**) out-of-plane (OOP) directions. (**c**) Coherence length (L_c) and (**d**) paracrystalline disorder parameter (g) extracted from the GIWAXS peaks as a function of $FeCl_3$ concentration. Calculated *d*-spacing of (**e**) π-π peak at (010) out-of-plane direction, (**f**) PM6 and Y6 lamellar peak at (100) in-plane direction, after incorporating 0-1 wt% $FeCl_3$ in PM6:Y6 blend films. See Supplementary Note 2 for detailed equation and Supplementary Table 1 for fitting results.

Grazing-incidence wide-angle X-ray scattering (GIWAXS) was employed to investigate the structural evolution of PM6:Y6 blends upon $FeCl_3$ doping. Both pristine and doped films exhibit

similar overall scattering features with preferred face-on molecular orientation, indicating that the bulk heterojunction morphology is largely preserved after doping. Distinct lamellar stacking peaks associated with PM6-rich (higher *q*) and Y6-rich (lower *q*) domains remain clearly observable across the entire doping series, consistent with the retention of phase-separated donor-acceptor packing motifs. In contrast to the largely unchanged lamellar spacing, the π-π stacking peak in the out-of-plane direction shifts toward higher *q* values with increasing $FeCl_3$ concentration, corresponding to reduced intermolecular π-π distances from 3.55 Å to 3.46 Å. Quantitative analysis further reveals that the coherence length associated with Y6 lamellar packing increases substantially, accompanied by reduced paracrystalline disorder, indicating improved packing uniformity within the acceptor-rich domains. In contrast, the structural parameters associated with PM6 remain largely unchanged, suggesting that the structural reorganization induced by $FeCl_3$ is highly selective to the acceptor phase.

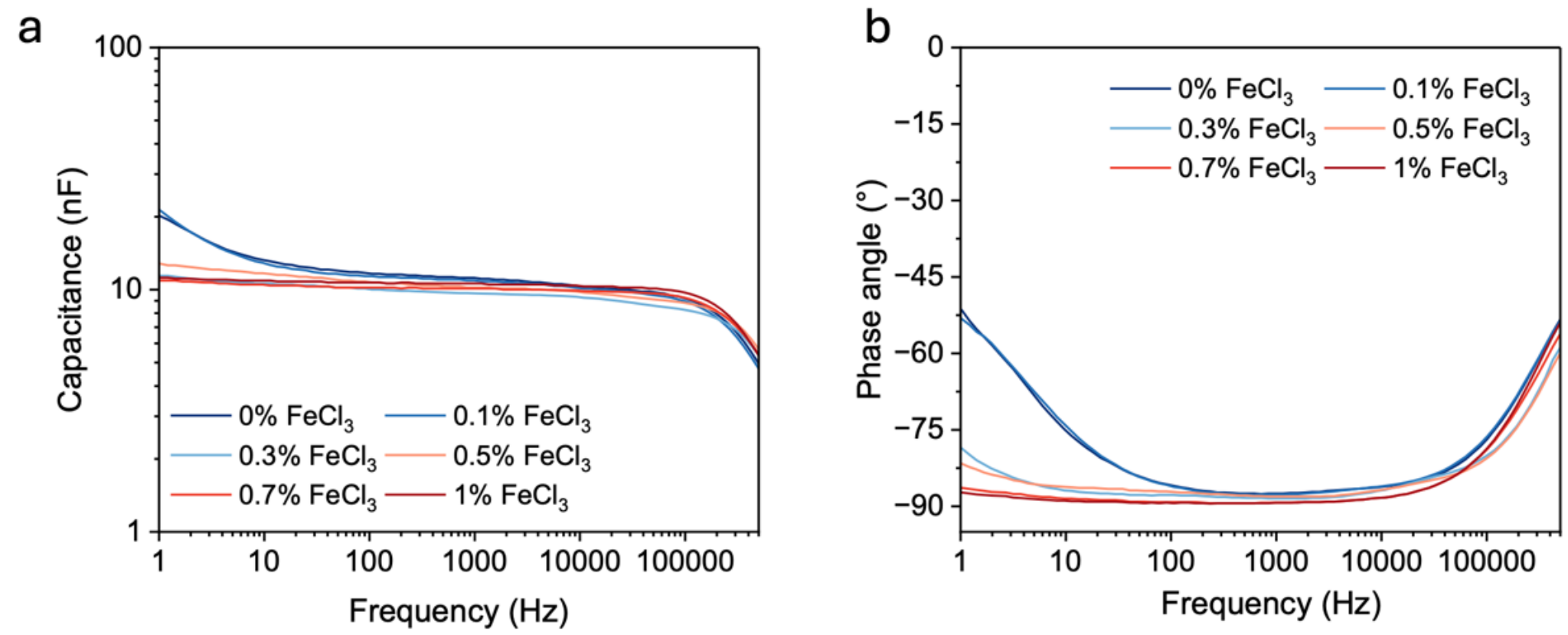

Fig. S6. Impedance analysis. (**a**) Capacitance-frequency and (**b**) phase angle-frequency spectra for inverted devices with different $FeCl_3$ concentrations.

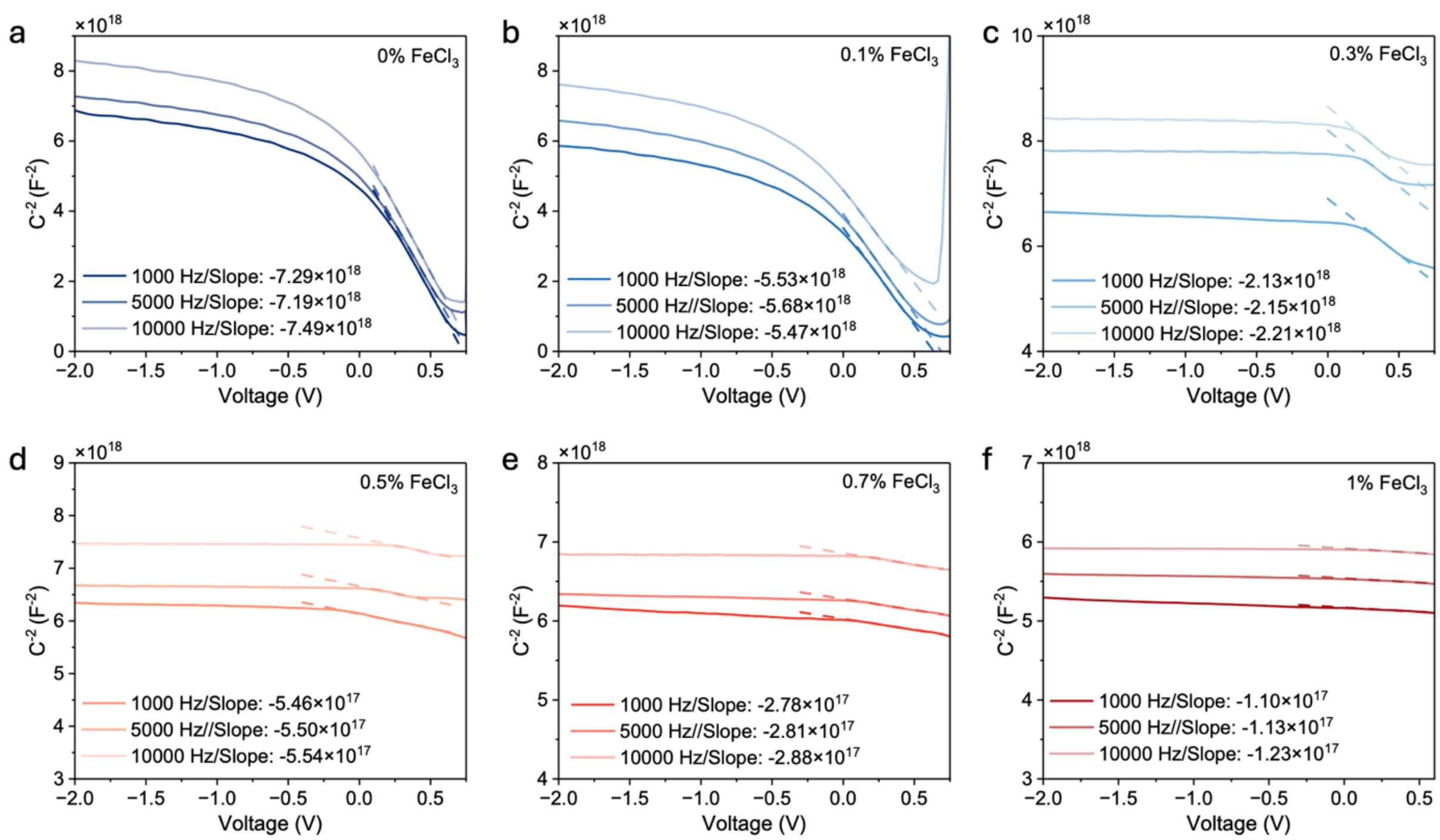

Fig. S7. Frequency-dependent Mott-Schottky analysis. Mott-Schottky plots of inverted devices with (**a-f**) 0-1 wt% $FeCl_3$ concentrations at 1, 5, 10 kHz. The solid lines represent measured data points and dashed lines represent linear fitting.

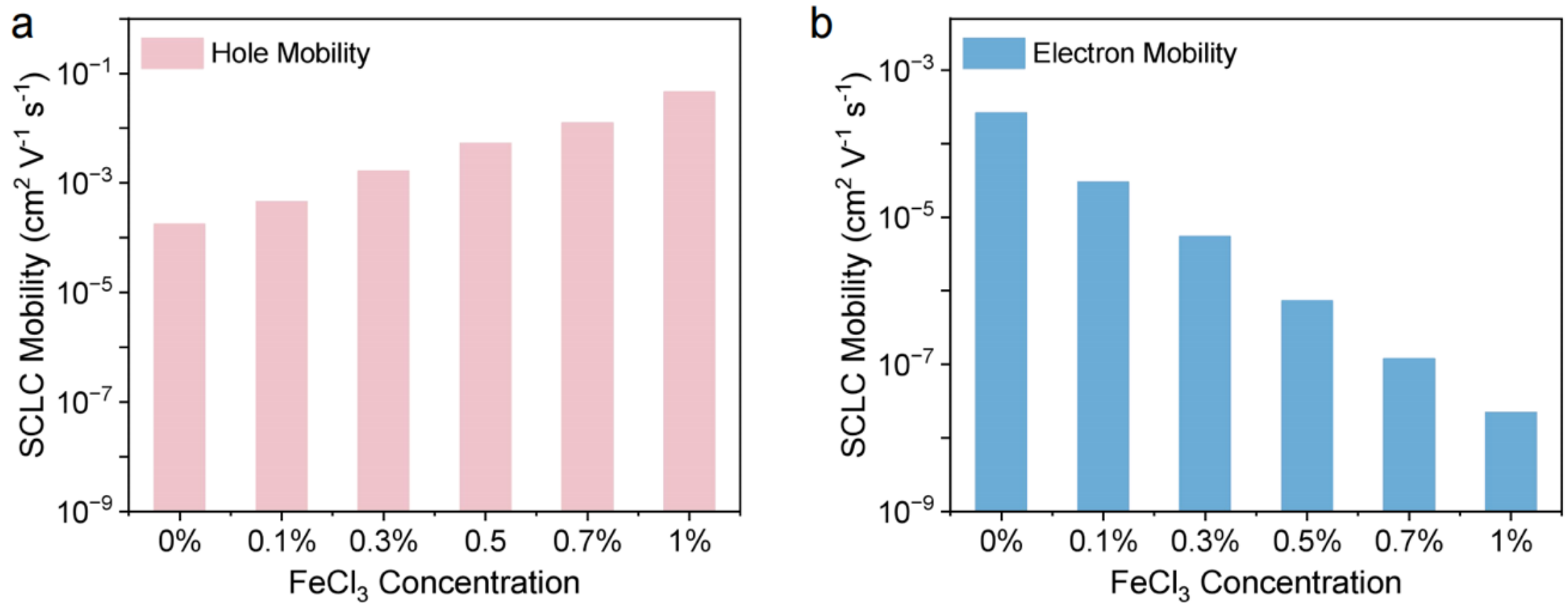

Fig. S8. Evolution of SCLC mobility. The calculated (**a**) hole mobility and (**b**) electron mobility by fitting the dark current to the model of single carrier space-charged limit current, which is described by: $J = \frac{9}{8}\varepsilon_0\varepsilon_r\mu\frac{V^2}{d^3}$, where J is the current density, μ is the carrier mobility, ε_0 is the permittivity of free space (8.85×10^{12} F/m), and ε_r is the relative permittivity of the material (assuming to be 3.5 for each active layer). V is the effective voltage, and d is the thickness of the active layer (300 nm), measured by the profilometer (Bruker Dektak XT).

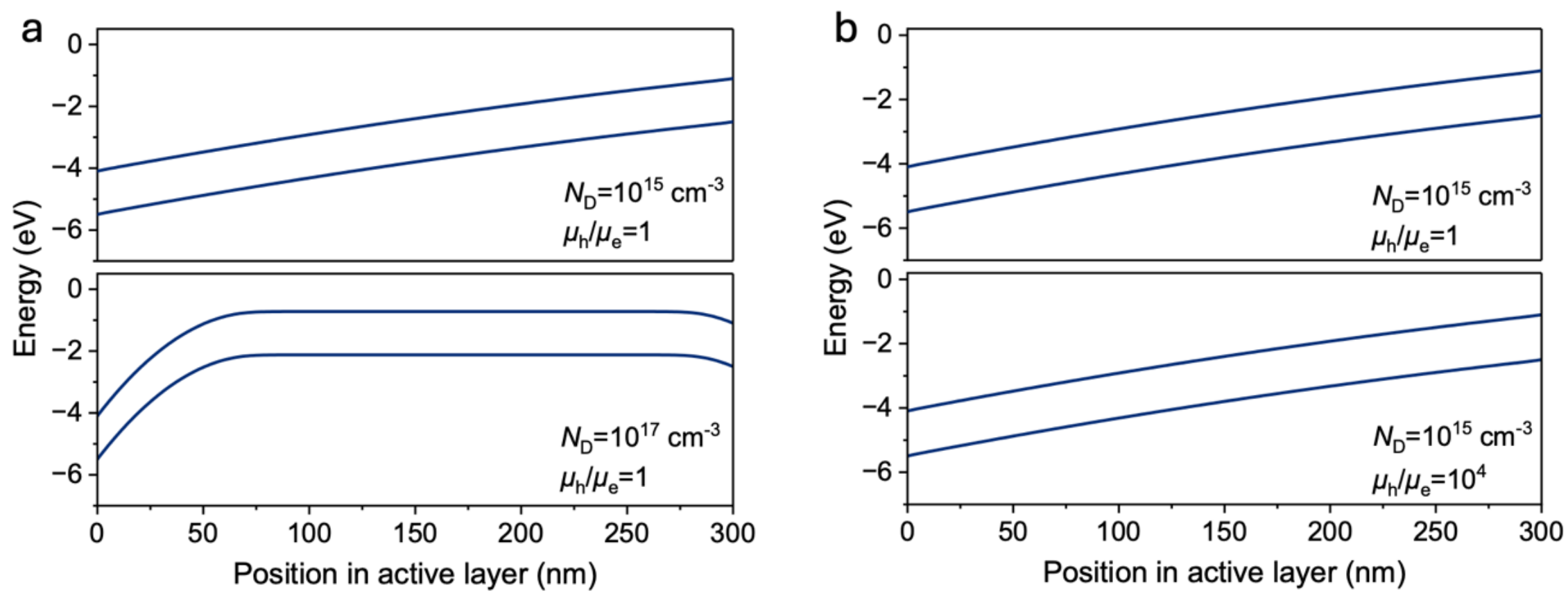

Fig. S9. Simulated band diagrams in the dark. Electric-field distributions of PM6:Y6 inverted devices with different (**a**) doping concentration and (**b**) mobility imbalance.

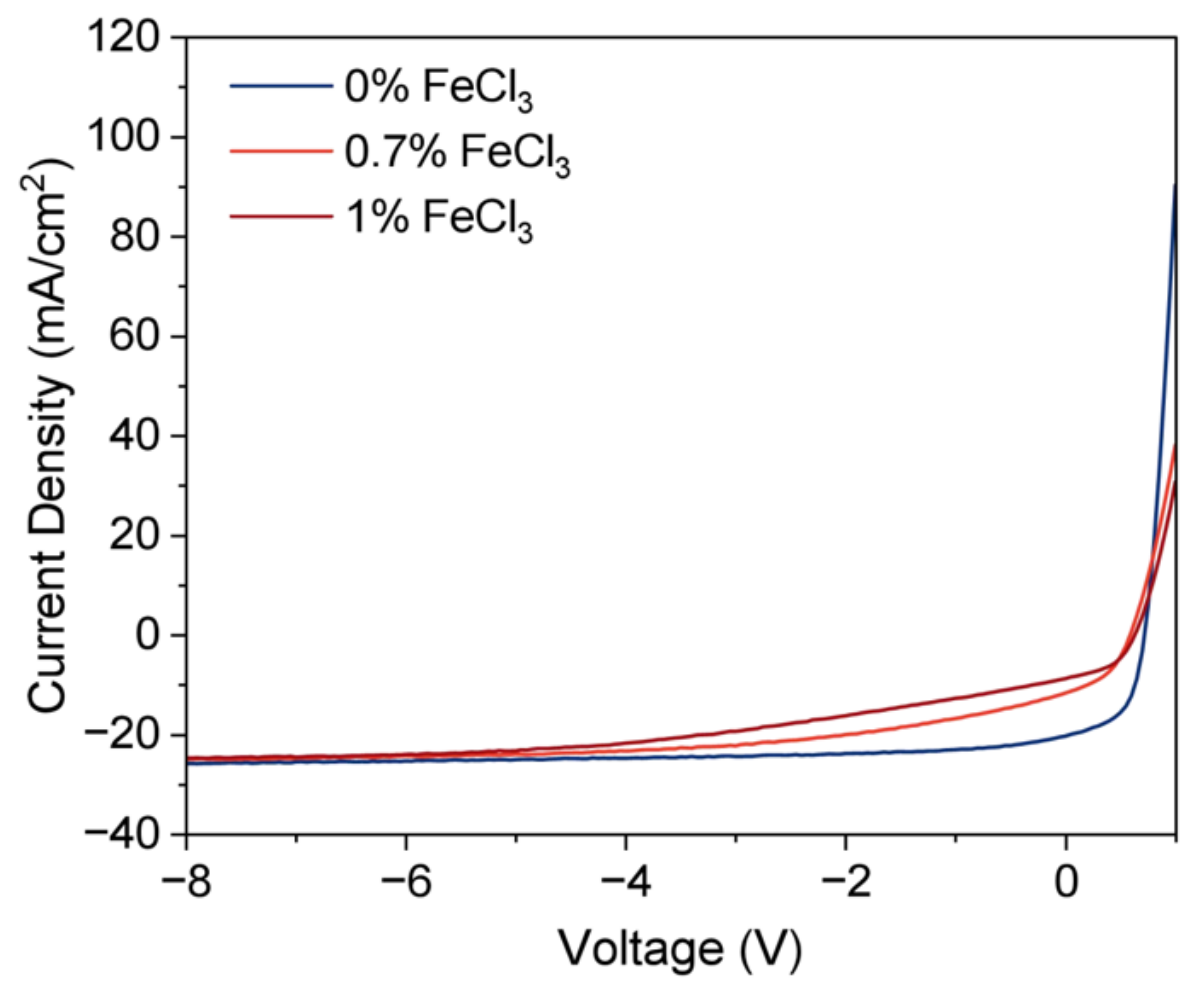

Fig. S10. Photocurrent characteristics. Light current density-voltage spectra of PM6:Y6 inverted OPD devices with different $FeCl_3$ concentrations.

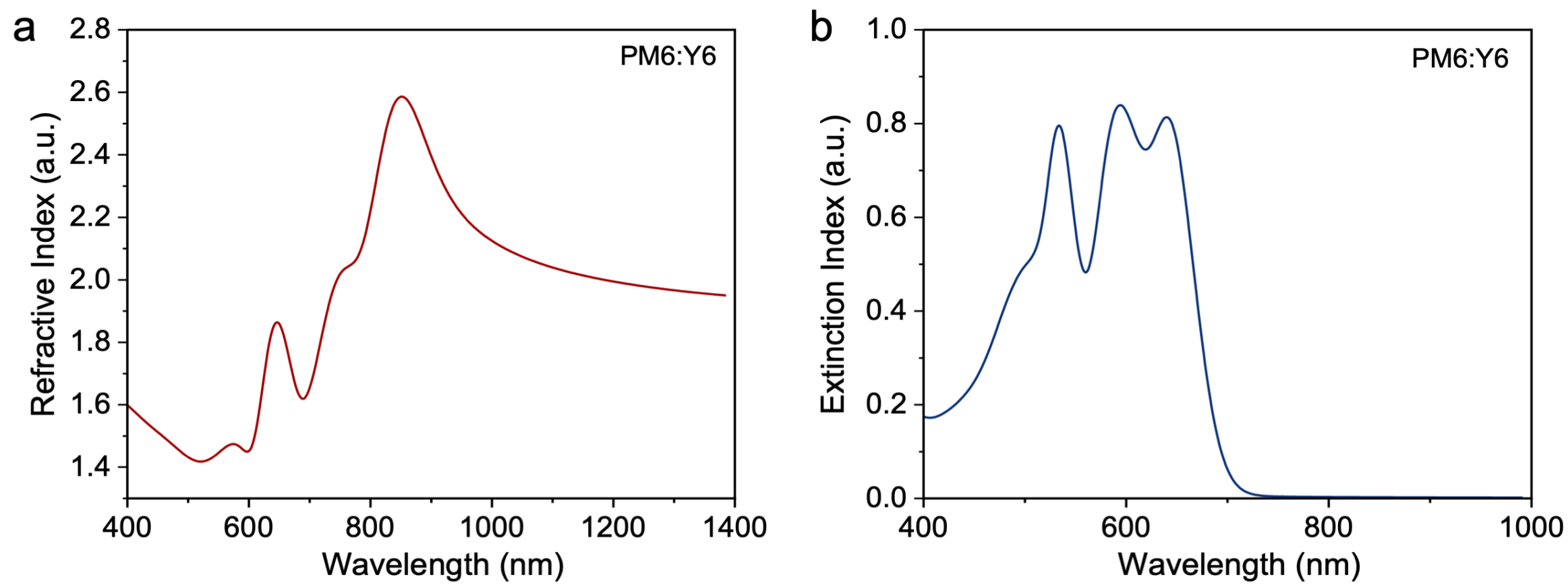

Fig. S11. Optical properties of PM6:Y6 film. (**a**) Refractive index and (**b**) extinction index profile of PM6:Y6 active layer.

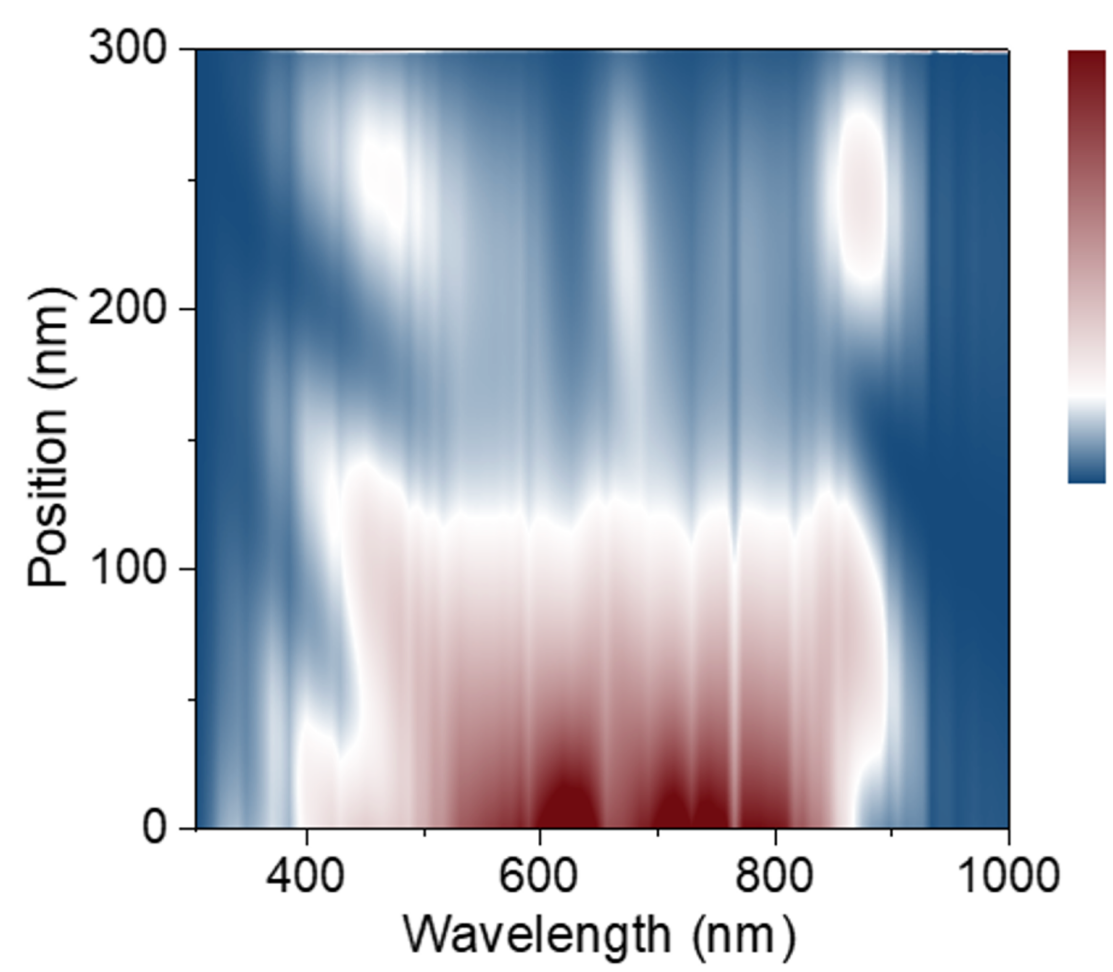

Fig. S12. Simulated spectrally and spatially resolved energy absorption profiles. The device architecture used here for simulations is : ITO(100 nm)/ZnO (20 nm)/PM6:Y6 blend (300 nm)/MoO_3 (10 nm)/Ag (100 nm).

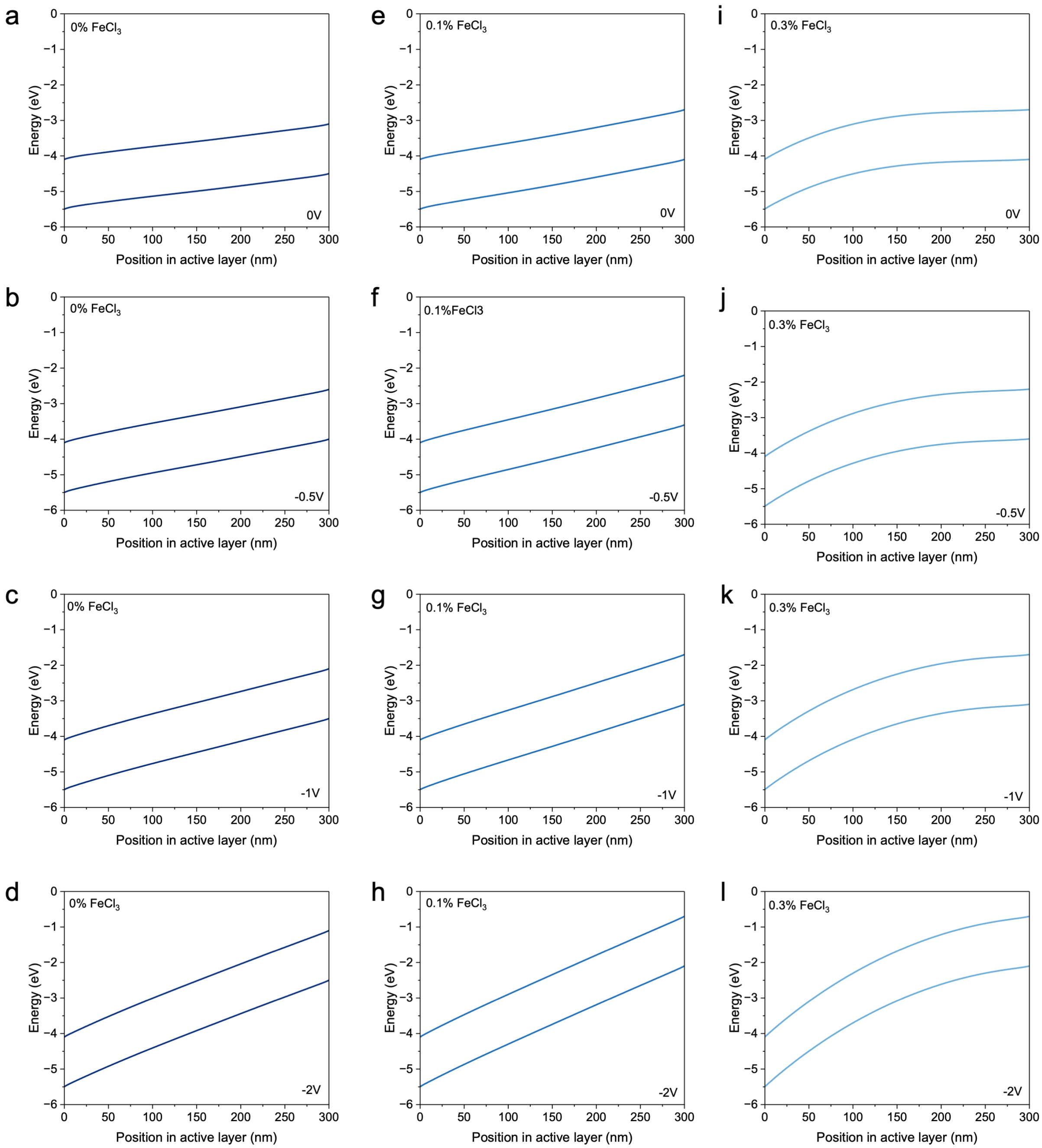

Fig. S13. Simulated band diagram under reverse bias. Computed energy band diagram from drift-diffusion simulation under illumination for (**a-d**) 0 wt% $FeCl_3$, (**e-h**) 0.1 wt% $FeCl_3$, (**i-l**) 0.3 wt% $FeCl_3$ at 0, -0.5, -1, -2V. The device structure is ITO (100 nm)/ZnO (20 nm)/PM6:Y6 (300 nm)/MoO_3 (10 nm)/Ag (100 nm).

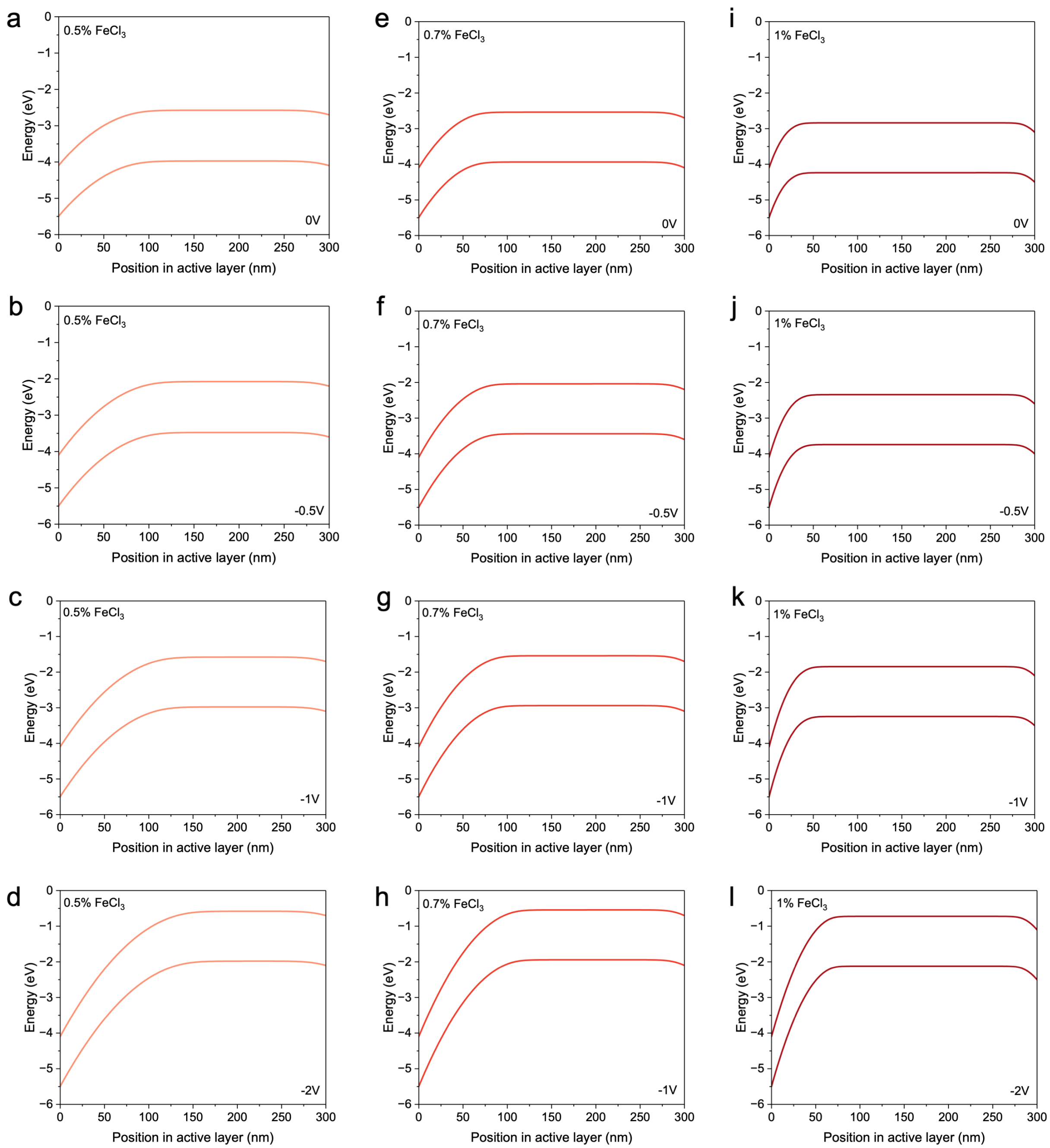

Fig. S14. Simulated band diagram under reverse bias. Computed energy band diagram from drift-diffusion simulation under illumination for (**a-d**) 0.5 wt% $FeCl_3$, (**e-h**) 0.7 wt% $FeCl_3$, (**i-l**) 1 wt% $FeCl_3$ at 0, -0.5, -1, -2V. The device structure is ITO (100 nm)/ZnO (20 nm)/PM6:Y6 (300 nm)/MoO_3 (10 nm)/Ag (100 nm).

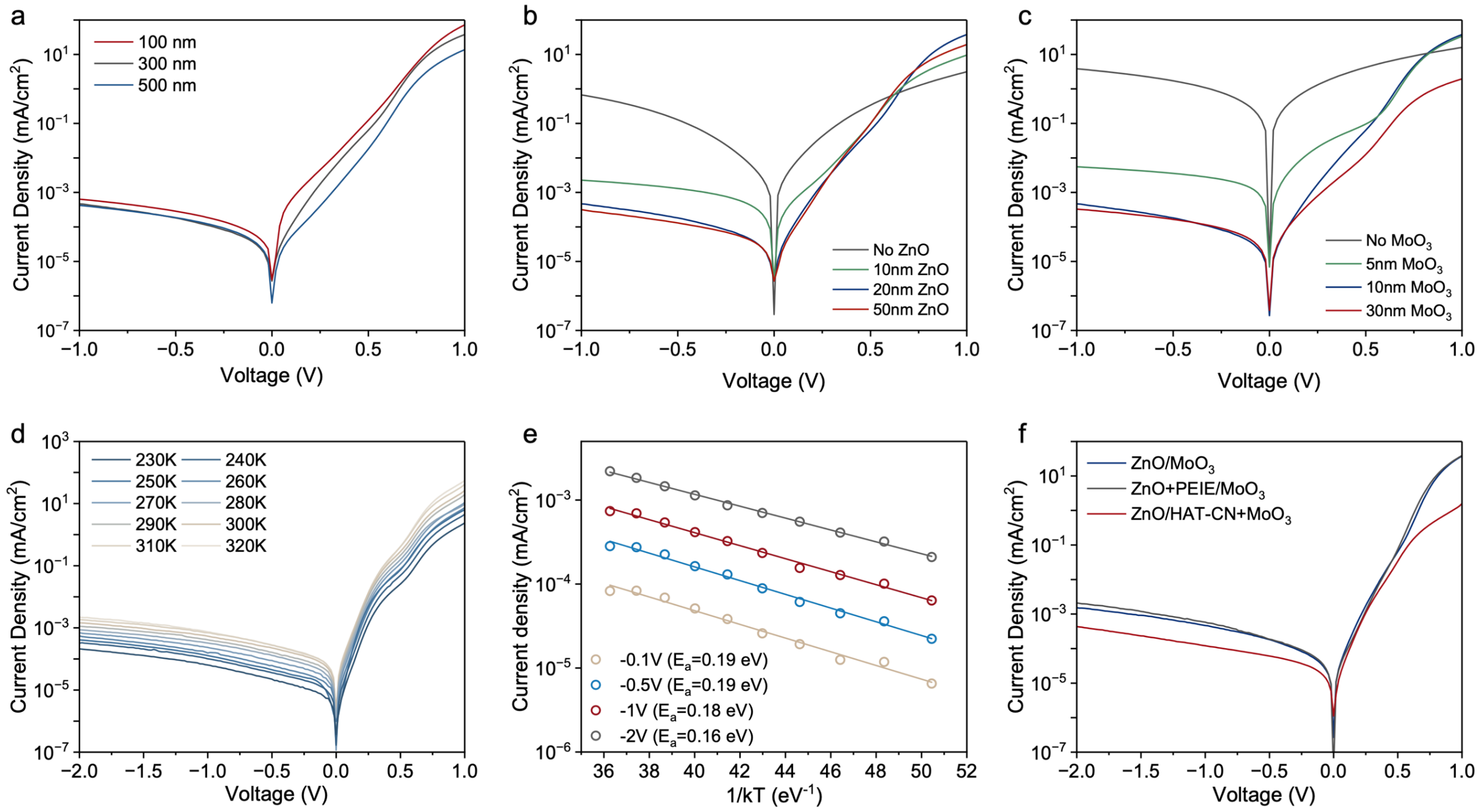

Fig. S15. Detailed device optimization. Current density-voltage characteristics of PM6:Y6 inverted OPD devices with varying (**a**) active layer thickness, (**b**) electron-transport layer ZnO thickness, and (**c**) hole-transport layer MoO_3 thickness. (**d**) Temperature-dependent current density-voltage characteristics of optimized PM6:Y6 inverted OPD device (300 nm active layer/20 nm ZnO/10 nm MoO_3). During this measurement, OPD devices were placed in cryostat under 10^{-1} torr vacuum. Temperature was set to 230 K first, and measurement was done by increasing temperature to 320 K. Each temperature was stabilized in 10-20 mins before recording the curve. (**e**) Calculated activation energy ($J_0 \propto \exp(-E_a/kT)$) at different reverse bias from Arrhenius equation. (**f**) Current density-voltage characteristics of PM6:Y6 inverted OPDs with different electron-transport layer and hole-transport layer.

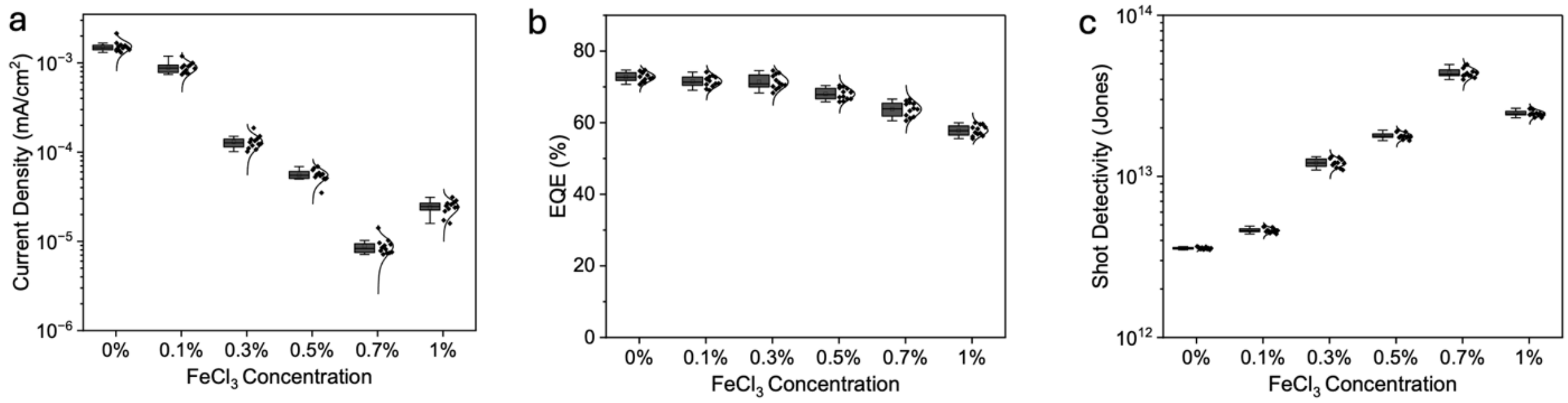

Fig. S16. Statistical distribution. Variation of (**a**) J_d at -2 V, (**b**) *EQE* at -2 V and 850 nm, and (**c**) shot detectivity at -2 V and 850 nm for PM6:Y6 inverted devices with different $FeCl_3$ concentrations.

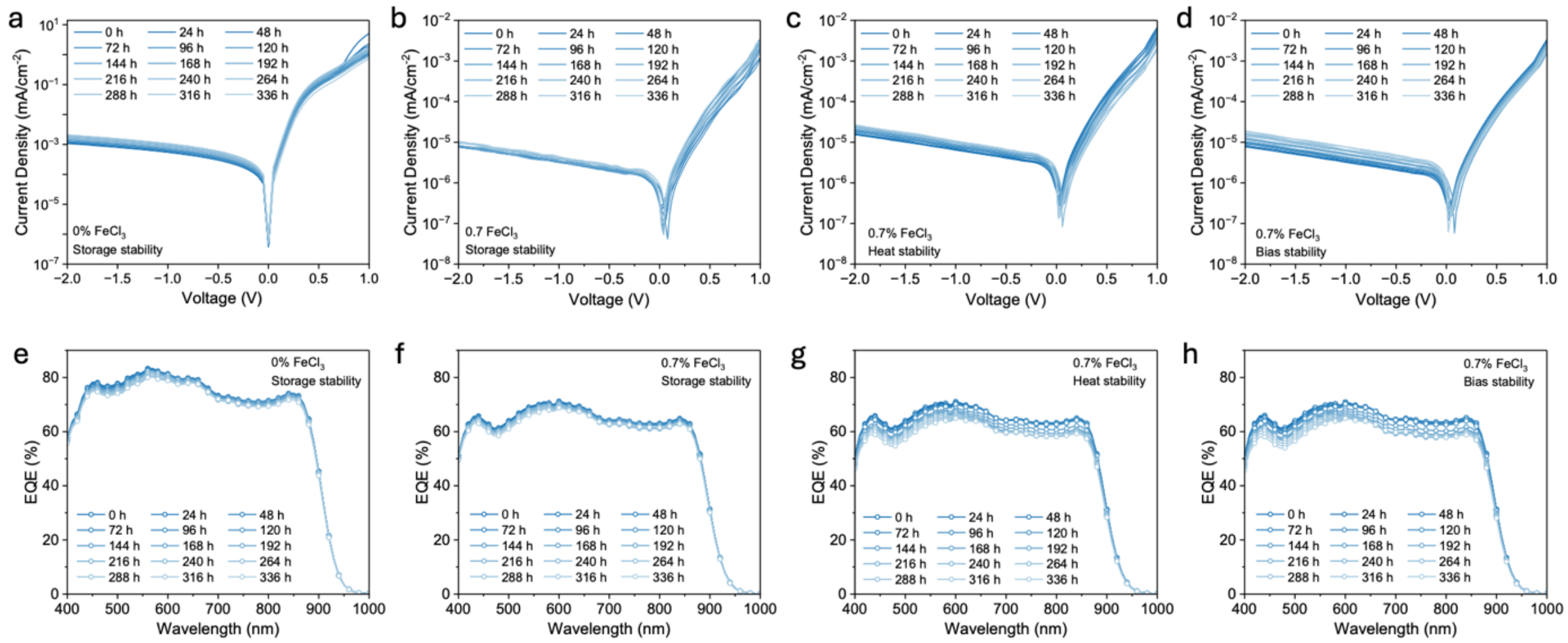

Fig. S17. Inverted devices stability test. (**a-d**) J_d spectra and (e-h) *EQE* spectra of 0 wt% $FeCl_3$ at storage and 0.7 wt% $FeCl_3$ at storage, 60 °C, or -2 V continued bias condition.

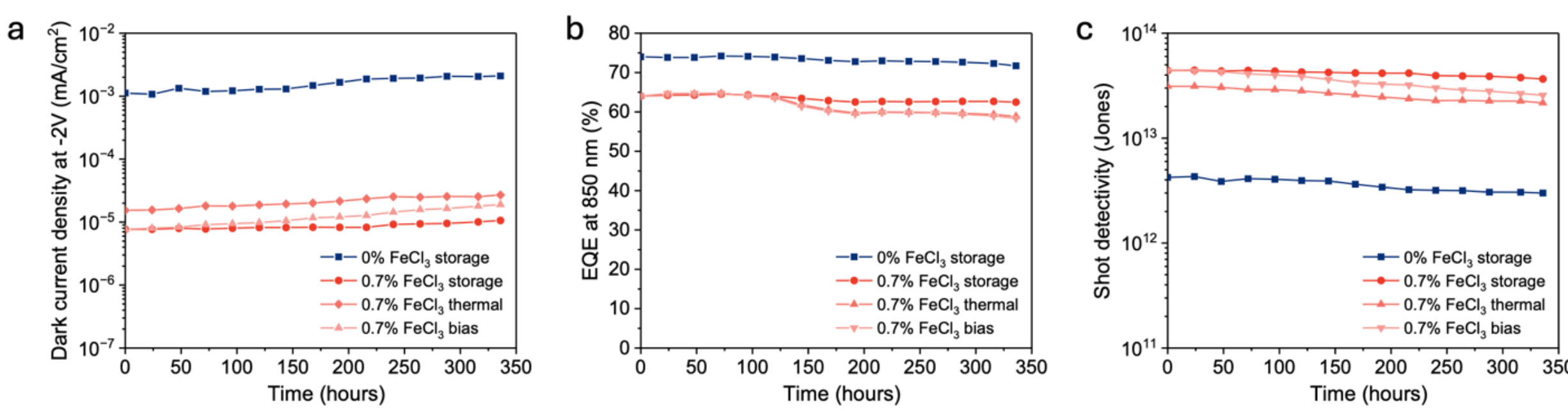

Fig. S18. Summarized inverted device stability results. (**a**) J_d at -2 V, (**b**) *EQE* at -2 V and 850 nm, and (**c**) shot detectivity at -2 V and 850 nm for PM6:Y6 inverted devices with different $FeCl_3$ concentrations at different conditions.

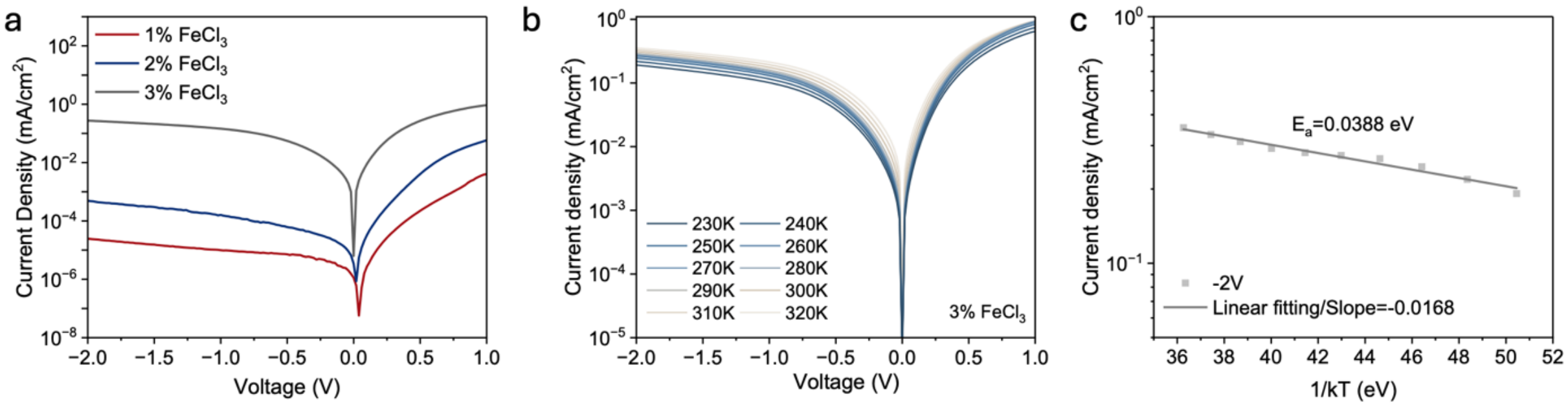

Fig. S19. Device performance at high $FeCl_3$ dopant concentrations. (**a**) Current density-voltage characteristics of PM6:Y6 inverted OPDs with 1-3 wt% $FeCl_3$ concentration. (**b**) Temperature-dependent dark-current characteristics of PM6:Y6+3 wt% $FeCl_3$ inverted devices. (**c**) Arrhenius plots of the dark current at -2 V, from which the activation energy (E_a) was extracted. This low E_a at high $FeCl_3$ concentrations indicates that carrier injection becomes less thermally activated, suggesting carrier injection is enhanced at high doping concentrations.

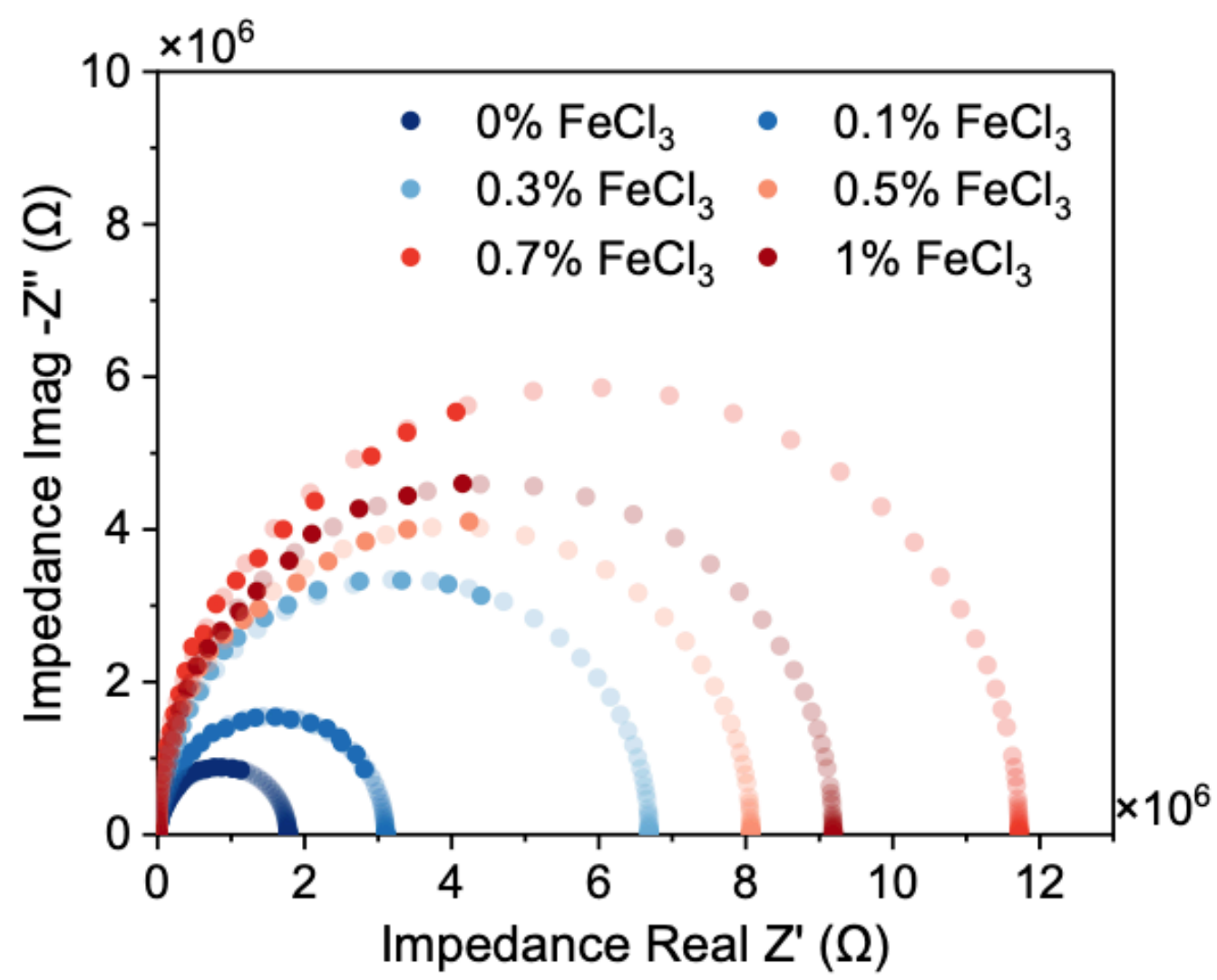

Fig. S20. Electrochemical impedance spectroscopy (EIS). Nyquist plots obtained from electrochemical impedance spectroscopy measurements under dark conditions at -2 V, showing the evolution of device impedance with doping concentration.

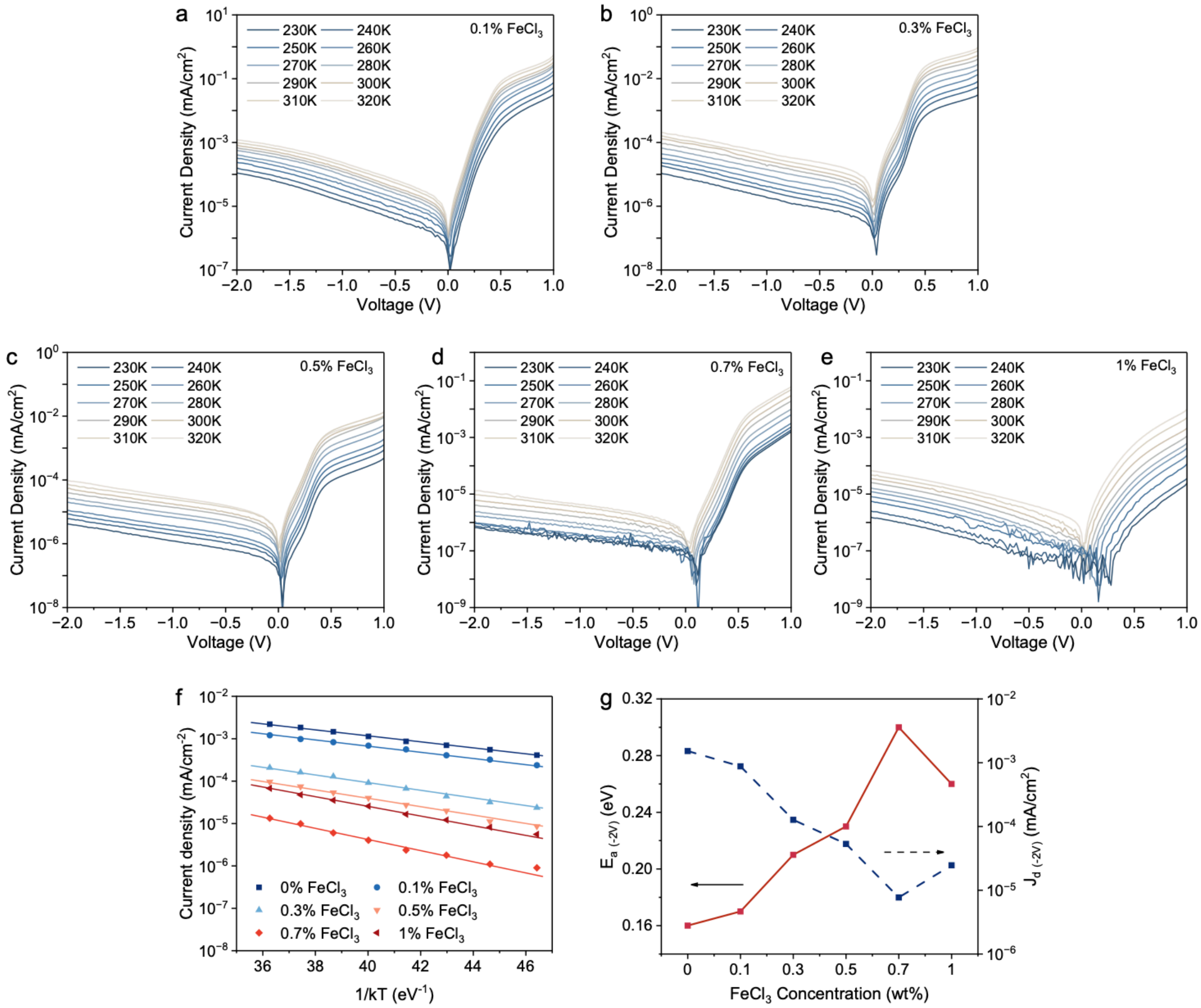

Fig. S21. Temperature dependent dark current density-voltage characteristics. PM6:Y6 inverted OPDs incorporated with (**a**) 0.1 wt%, (**b**) 0.3 wt%, (**c**) 0.5 wt%, (**d**) 0.7 wt%, and (**e**) 1 wt% $FeCl_3$. (**f**) Temperature-dependent dark current density measured at -2 V for devices with varying $FeCl_3$ concentrations, solid lines represent linear fitting for all data points. (**g**) Extracted activation energy and dark current density as a function of $FeCl_3$ concentration. At low temperatures, the dark current in some devices approaches the measurement noise floor, leading to increased fluctuation in the *J-V* curves. This is mainly due to the limited current resolution of the measurement setup at very low current levels. For activation energy extraction, however, J_d was taken at -2 V, where the measured current is well above the instrumental noise floor for all devices. Therefore, the extracted temperature dependence and corresponding activation energies are not affected by the low-current noise observed at lower bias or lower temperature regions.

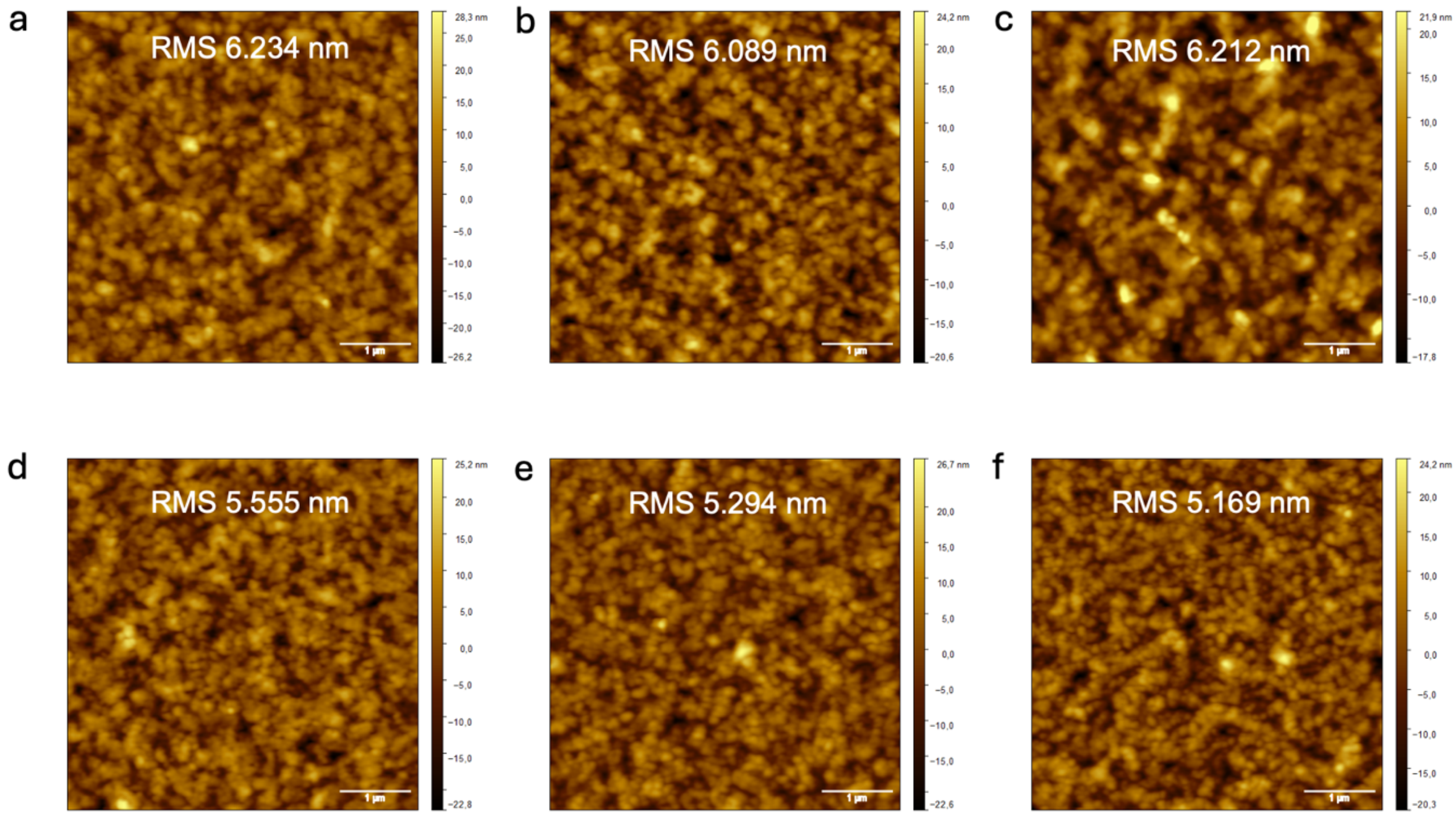

Fig. S22. Atomic force microscopy (AFM) images. Height images of PM6:Y6 film (300 nm) with (**a**) 0 wt%, (**b**) 0.1 wt%, (**c**) 0.3 wt%, (**d**) 0.5 wt%, (**e**) 0.7 wt%, (**f**) 1 wt% $FeCl_3$ concentration.

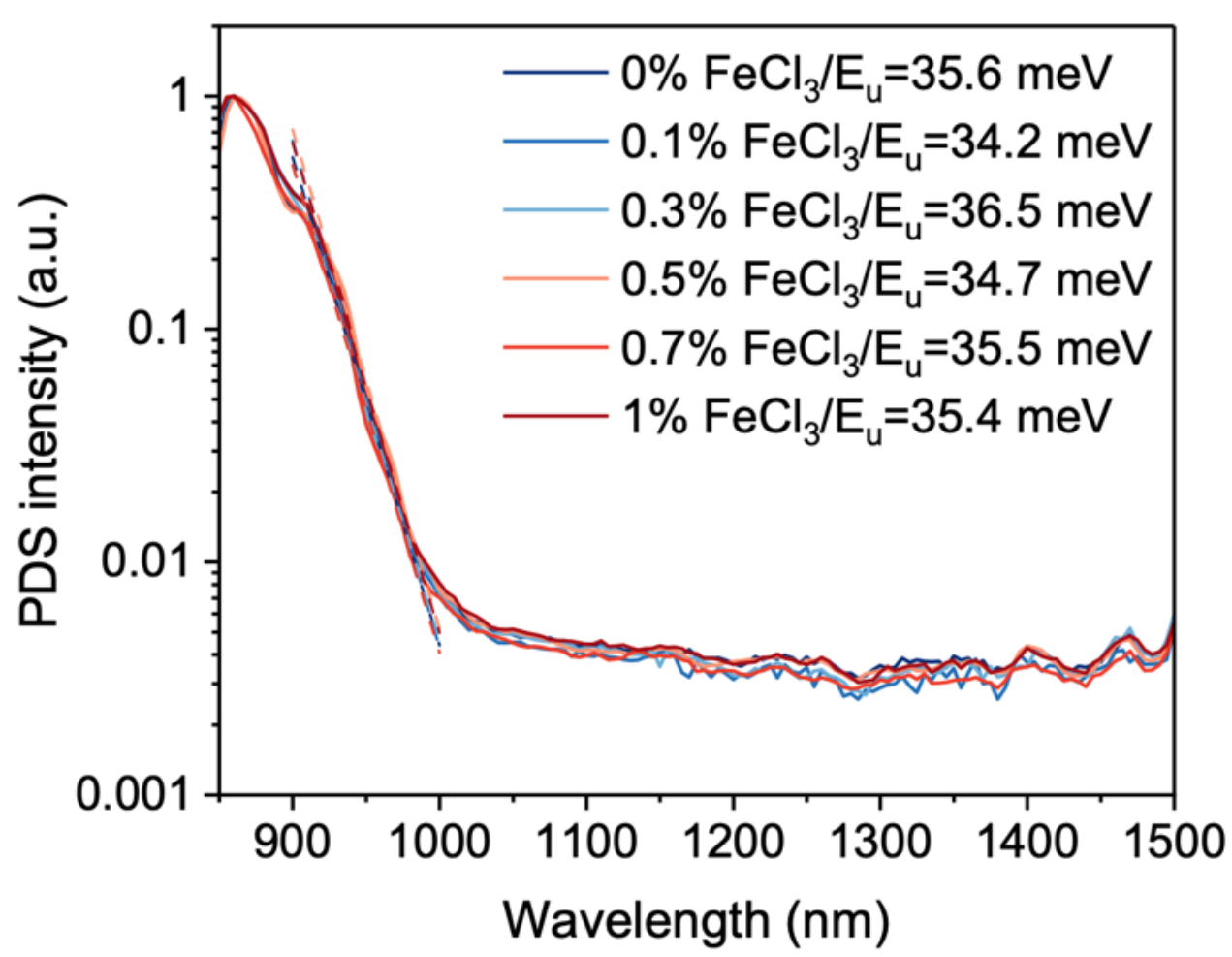

Fig. S23. Photothermal deflection spectroscopy (PDS) spectra. The solid lines represent test data and dashed lines represent linear fitting for Urbach energies (E_u).

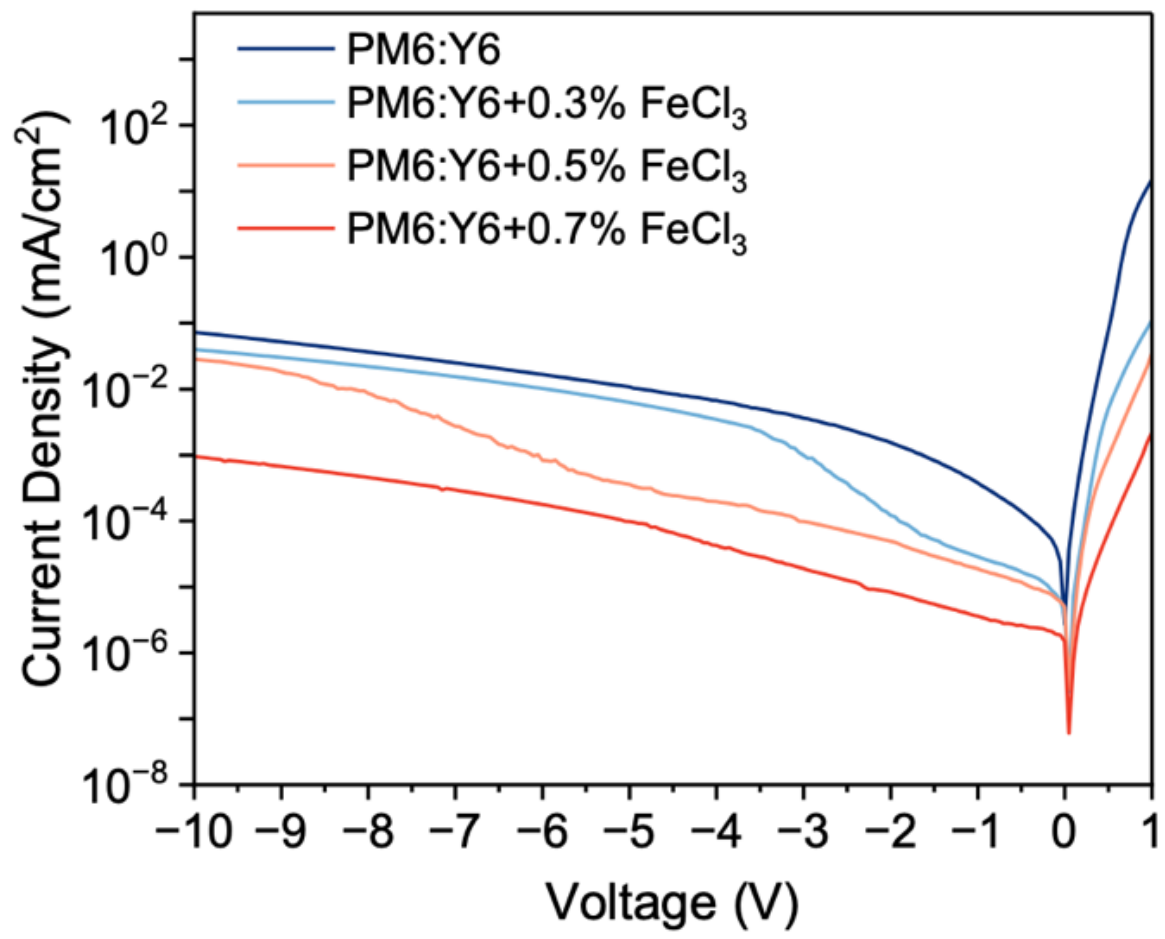

Fig. S24. Dark *J-V* characteristics of PM6 OPDs with different $FeCl_3$ doping concentrations measured over an extended reverse-bias range. The 0.3 wt% and 0.5 wt% $FeCl_3$-doped devices exhibit a distinct transition at higher reverse biases, where the initially suppressed dark current increases rapidly and gradually approaches that of the undoped device, consistent with the bias-induced expansion of the high-field region and the corresponding reduction of the low-field blocking region.

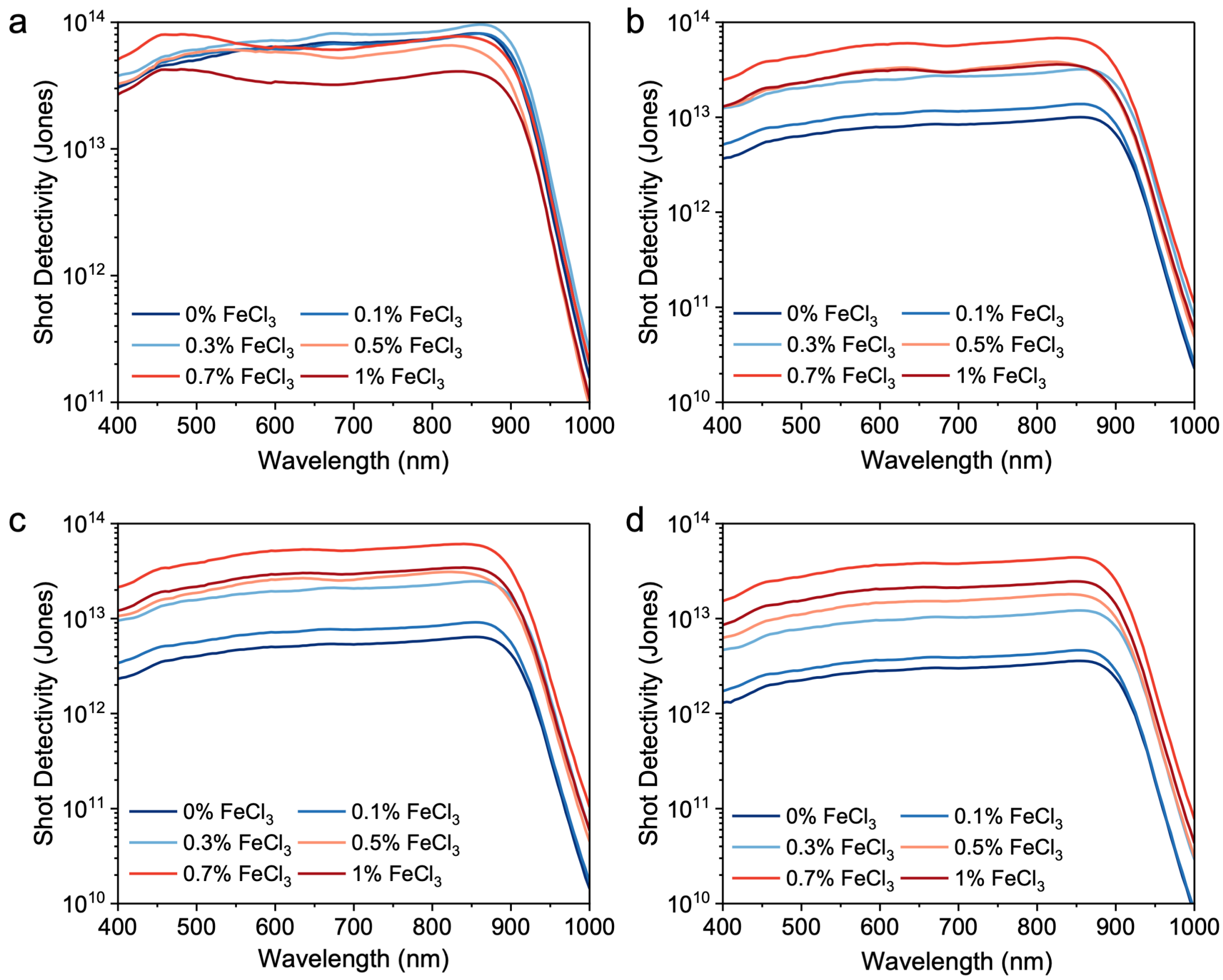

Fig. S25. Voltage-dependent shot-noise limited detectivity. Calculated shot detectivity of PM6:Y6 inverted OPDs with 0-1 wt% $FeCl_3$ at (**a**) 0 V, (**b**) -0.5 V, (**c**) -1 V, and (**d**) -2 V. Shot noise detectivity given in Jones by: $D^*_{\mathrm{sh}} = \frac{R\sqrt{A}}{\sqrt{2qI_{\mathrm{d}}}}$, where I_d is the dark current, A is the device area (0.04 cm^2), q is the elementary charge. Under -2 V reverse bias, the detectivity of the undoped PM6:Y6 device decreases by more than one order of magnitude, whereas the PM6:Y6 with 0.7 wt% $FeCl_3$ device retains a high detectivity exceeding 10^{13} Jones.

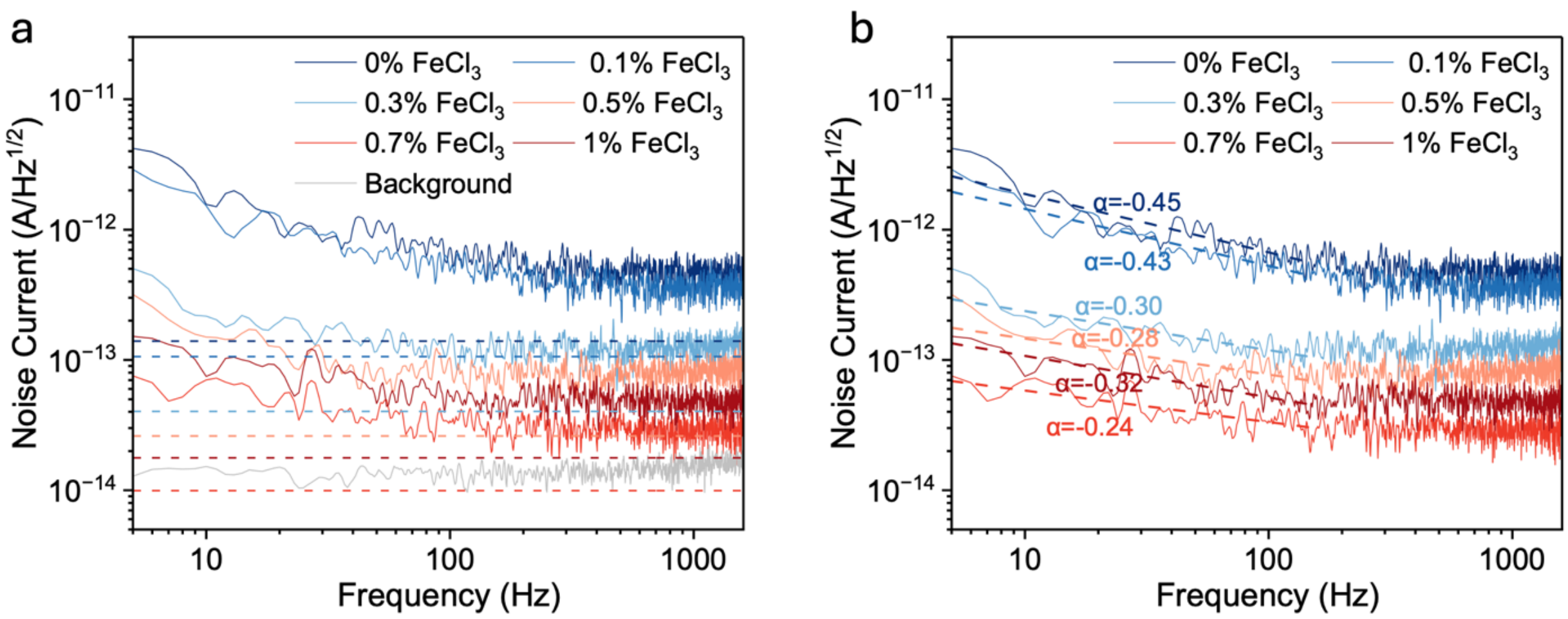

Fig. S26. Frequency-dependent noise current spectrum. Noise current spectrum plotted as a function of frequency for PM6:Y6 inverted OPD with 0-1 wt% $FeCl_3$ dopant obtained at -2 V. (**a**) The grey line marks the noise floor of the background. Note that this noise floor is close to the instrumental noise current density of the transimpedance amplifier provided in the manual (13 $fA/Hz^{1/2}$ under 10^8 low-noise mode gain setting). The dashed lines in the spectrum represent the shot-noise limit for each device. Shot noise is calculated from dark current density at -2 V by $I_n=\sqrt{2qI_d\Delta f}$. Here q is the elementary charge and Δf is the electrical bandwidth which is set to 1 Hz. (**b**) The dashed lines in the spectrum represent the fitting for $1/f$ noise in each device based on the equation: $I_f=A/f^{\alpha}$. The fitted low-frequency noise amplitude decreases after $FeCl_3$ incorporation, indicating a reduced contribution of $1/f$ noise to the total noise.

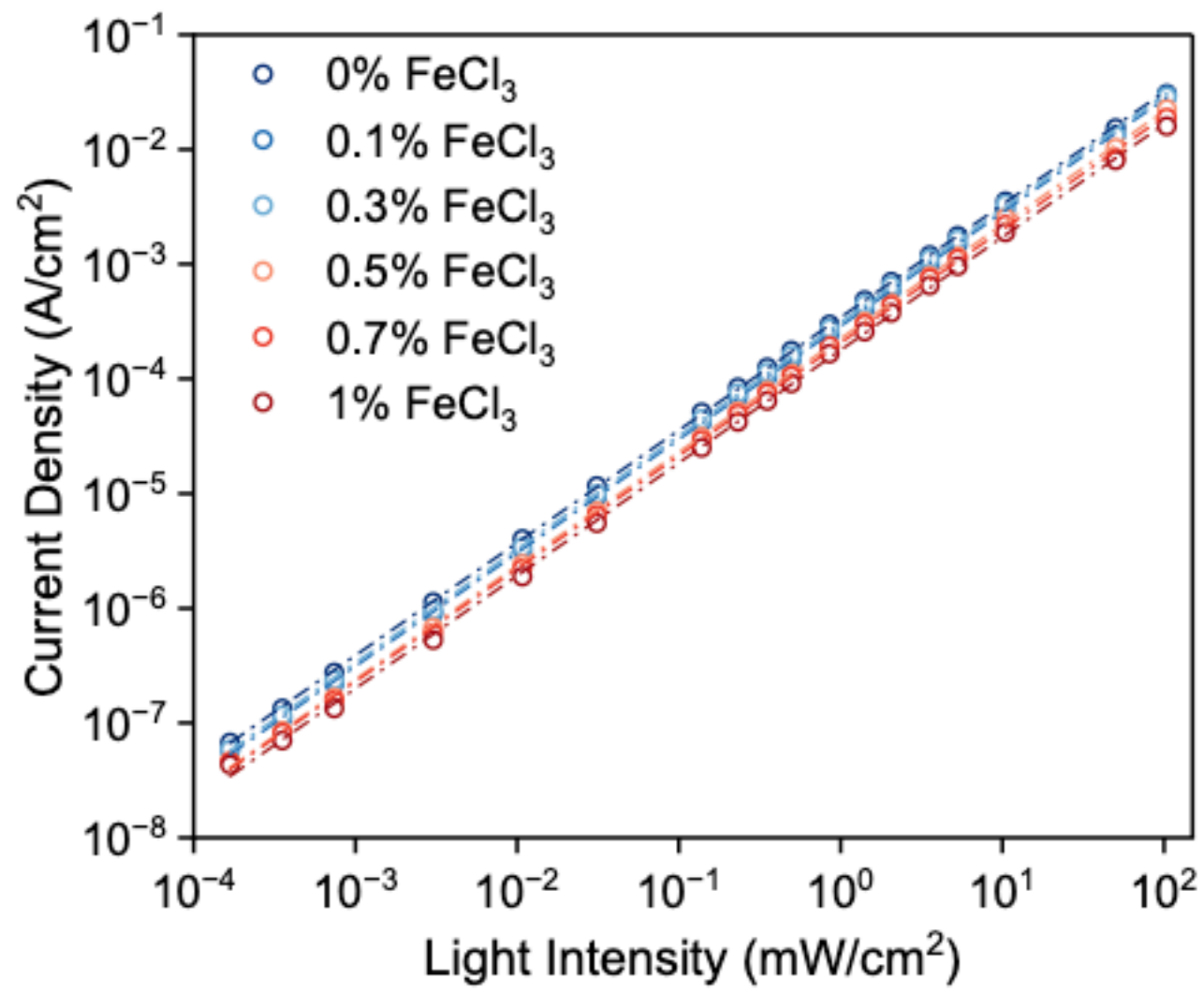

Fig. S27. Linear dynamic range. Photocurrent density as a function of incident light intensity measured at -2 V. All devices exhibit a linear photoresponse over a wide illumination range over six orders of magnitude, corresponding to a long linear dynamic range.

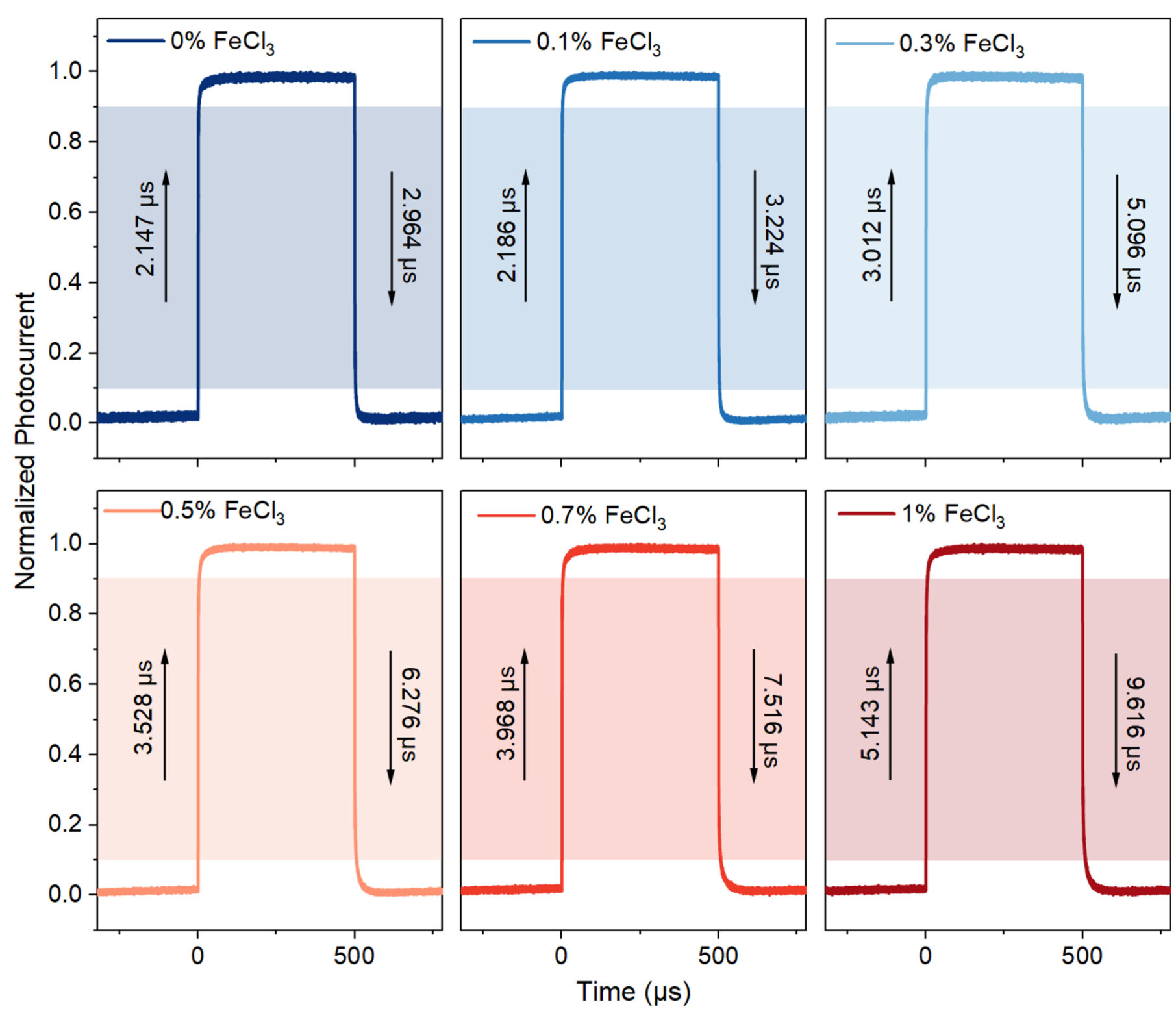

Fig. S28. Photoresponse time. Normalized photocurrent of PM6:Y6 inverted OPDs as a function of time under -2 V reverse bias. The shaded region indicates the 10-90% change in photocurrent, used to define the rise and fall times.

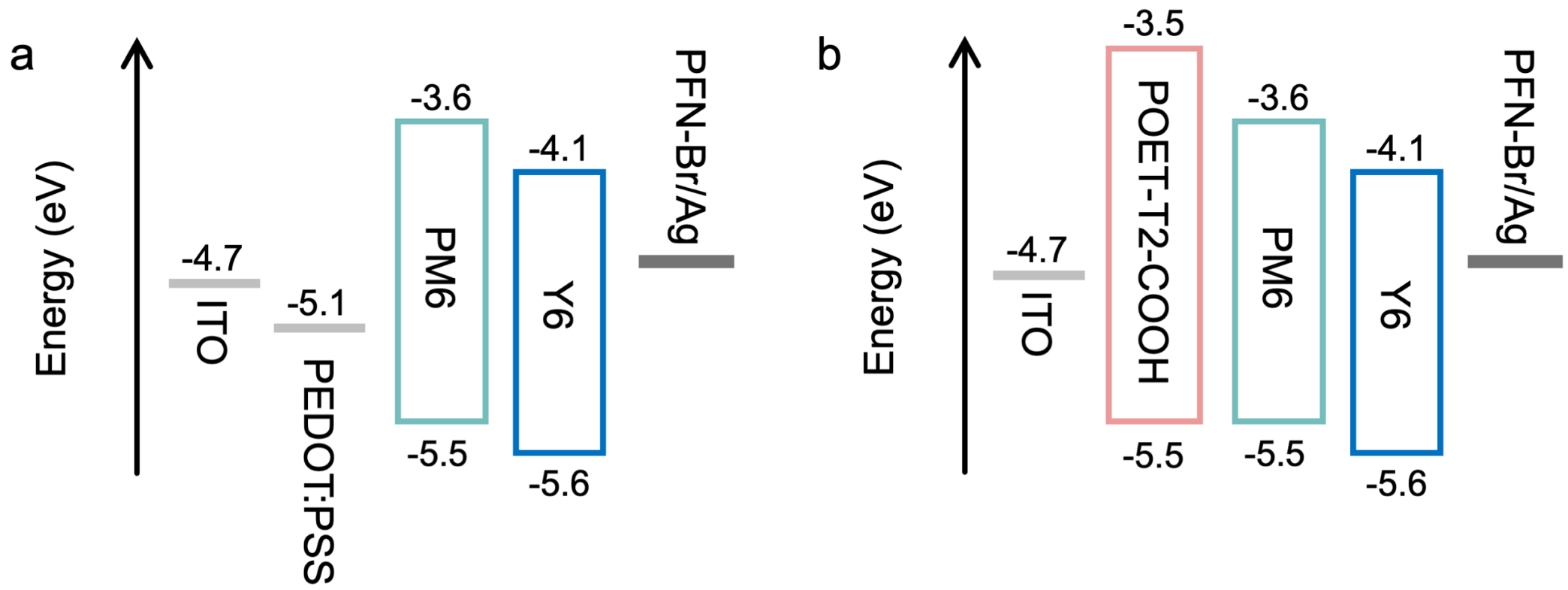

Fig. S29. Energy level diagrams. PM6:Y6 conventional OPDs with (**a**) PEDOT:PSS and (**b**) POET-T2-COOH as hole-transport layer. The energy level of POET-T2-COOH is obtained from the cyclic-voltammetry (CV) measurement.[8]

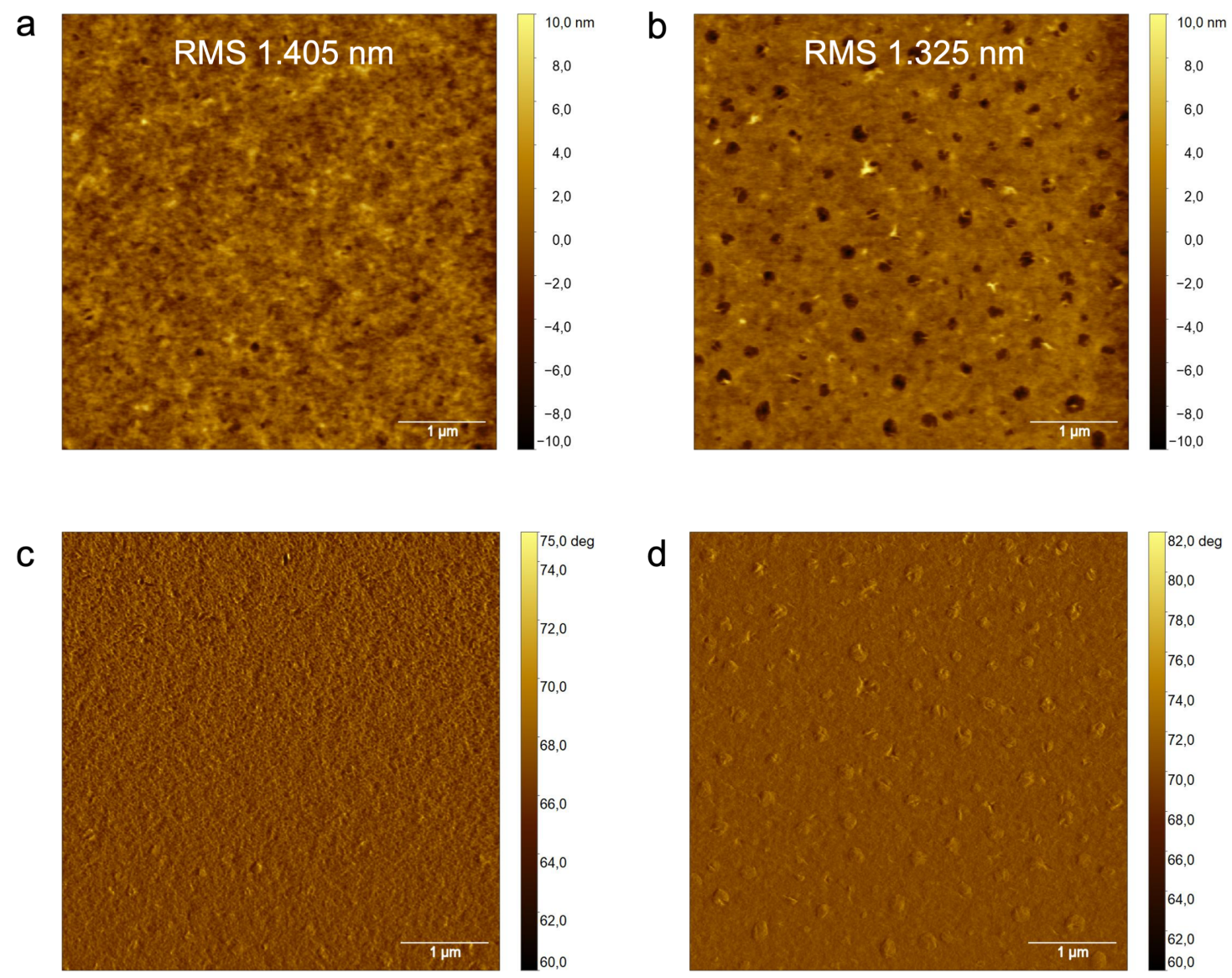

Fig. S30. Atomic force microscopy (AFM) images. Height images of (**a**) POET-T2 film (before side-chain cleavege) and (**b**) POET-T2-COOH film (after thermally side-chain cleavege). Phase images of (**c**) POET-T2 film (before side-chain cleavege) and (**d**) POET-T2-COOH film (after thermally side-chain cleavege).

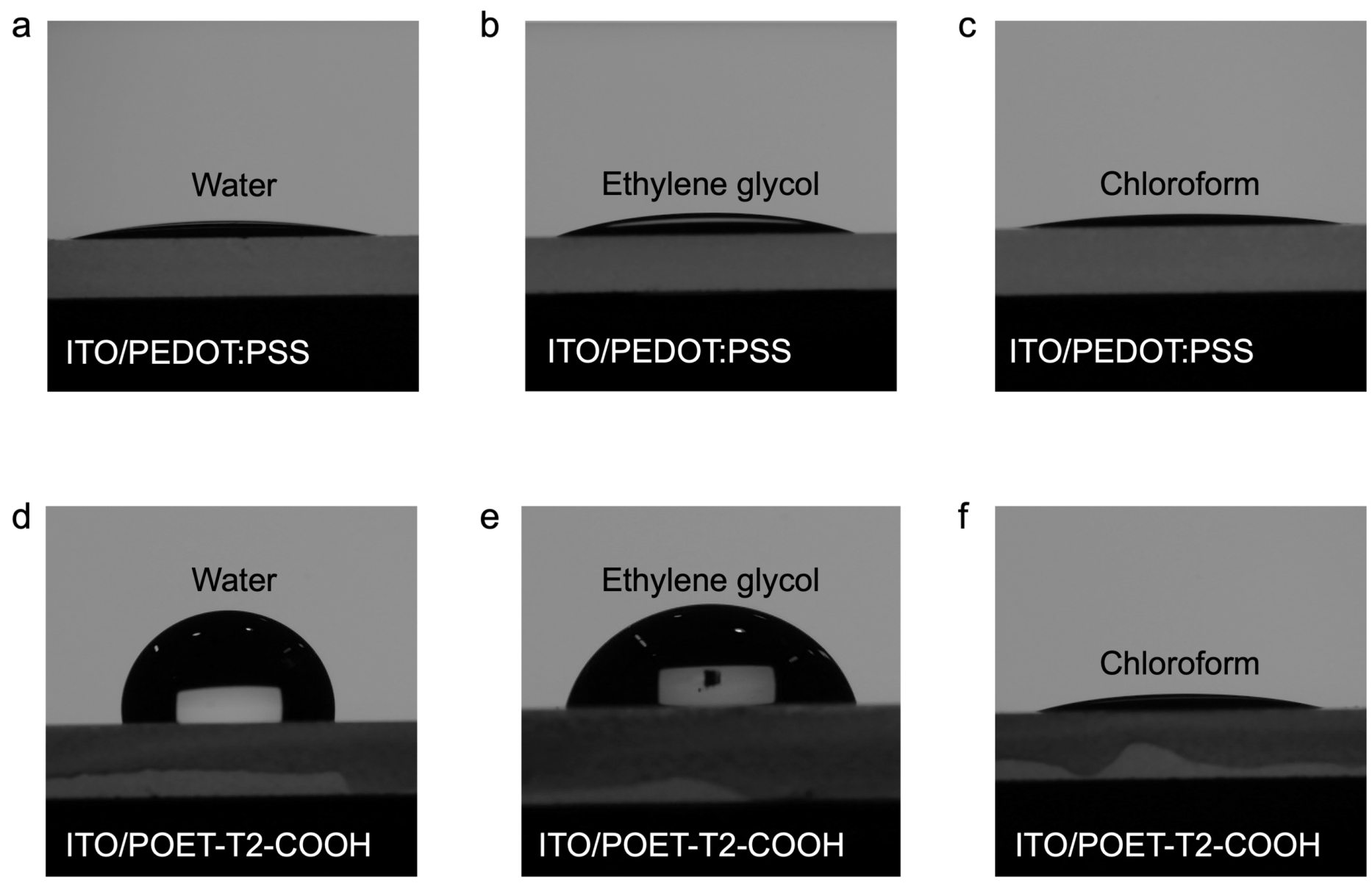

Fig. S31. Surface characterizations of POET-T2-COOH film. Static contact angle measurements with (**a, d**) water, (**b, e**) ethylene glycol, and (**c, f**) chloroform on top of PEDOT:PSS and POET-T2-COOH. Compared to PEDOT:PSS, the POET-T2-COOH film is more hydrophobic. For chloroform, both films show near-zero contact angles, indicating good wettability in terms of active layer solution.

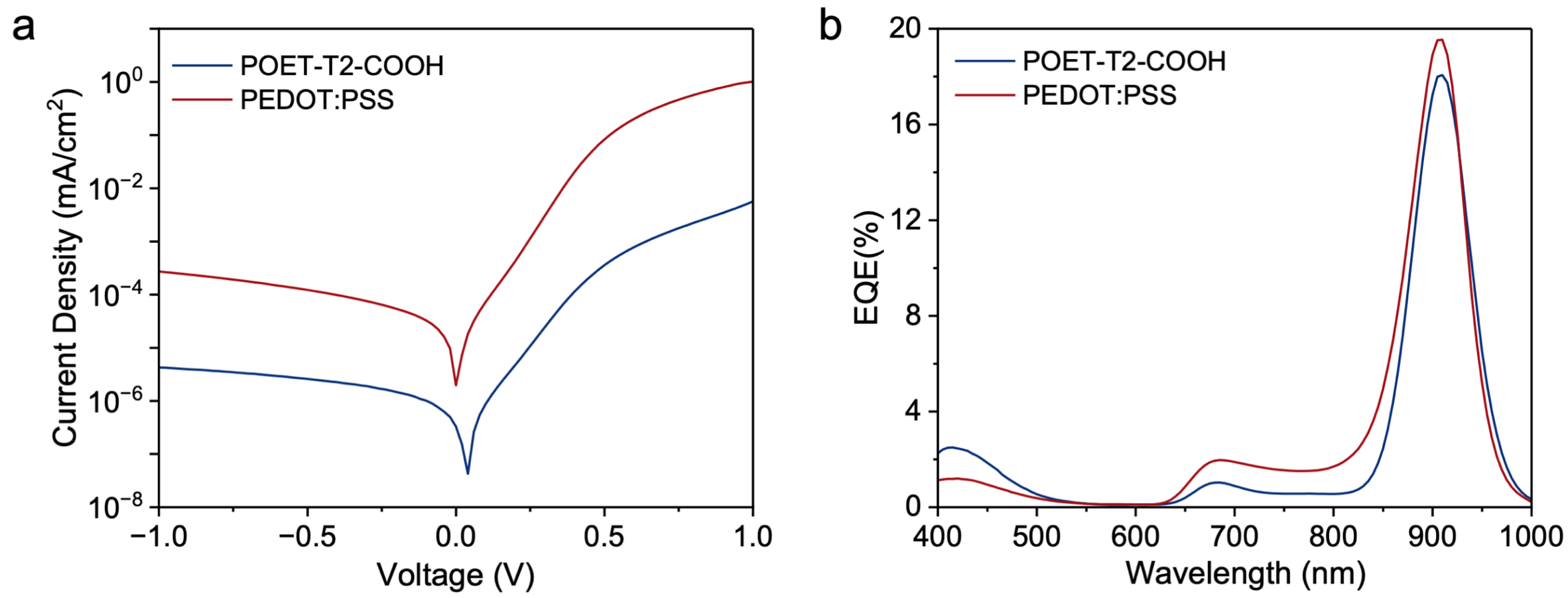

Fig. S32. Comparison of device performance between two interlayers. (**a**) Current density-voltage characteristics and (**b**) *EQE* response of PM6:Y6+0.2 wt% $FeCl_3$ conventional OPDs (800 nm) with PEDOT:PSS and POET-T2-COOH. Devices based on POET-T2-COOH exhibit a dark current that is more than one order of magnitude lower than those based on PEDOT:PSS. This reduction is primarily attributed to the deeper HOMO level of POET-T2-COOH, which increases the electron injection barrier and suppresses injection-driven dark current. In addition, POET-T2-COOH shows slightly reduced electrical conductivity compared to PEDOT:PSS, leading to an increased series resistance. As a result, a modest reduction in *EQE* (~2-3%) is observed due to less efficient carrier extraction.

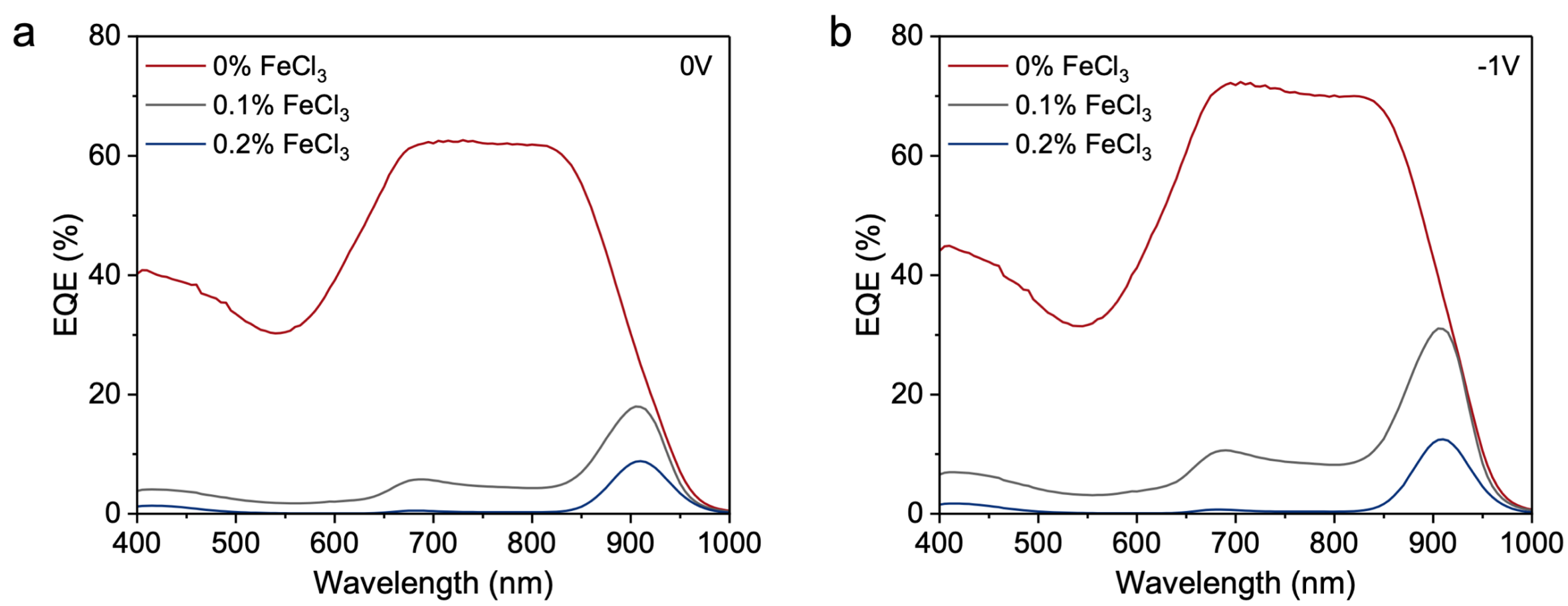

Fig. S33. Voltage-dependent narrowband photoresponse. *EQE* spectra of PM6:Y6 conventional OPDs (800 nm) with different $FeCl_3$ dopant concentration at (**a**) 0 V and (**b**) -1 V.

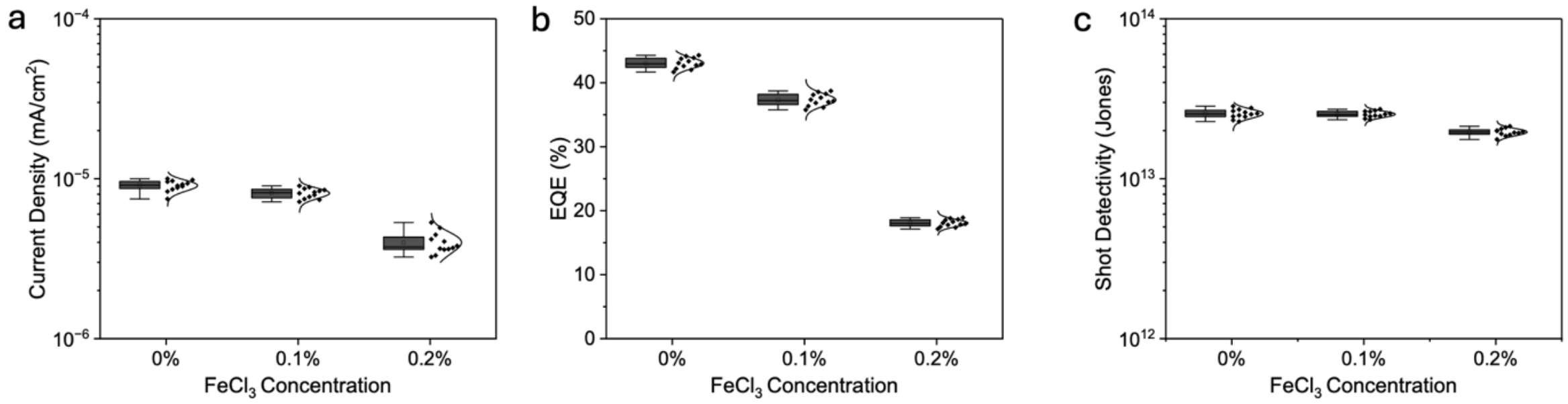

Fig. S34. Statistical distribution. Variation of (**a**) J_d at -2 V, (**b**) *EQE* at -2 V and 910 nm, and (**c**) shot detectivity at -2 V and 910 nm for PM6:Y6 conventional narrowband devices with different $FeCl_3$ concentrations.

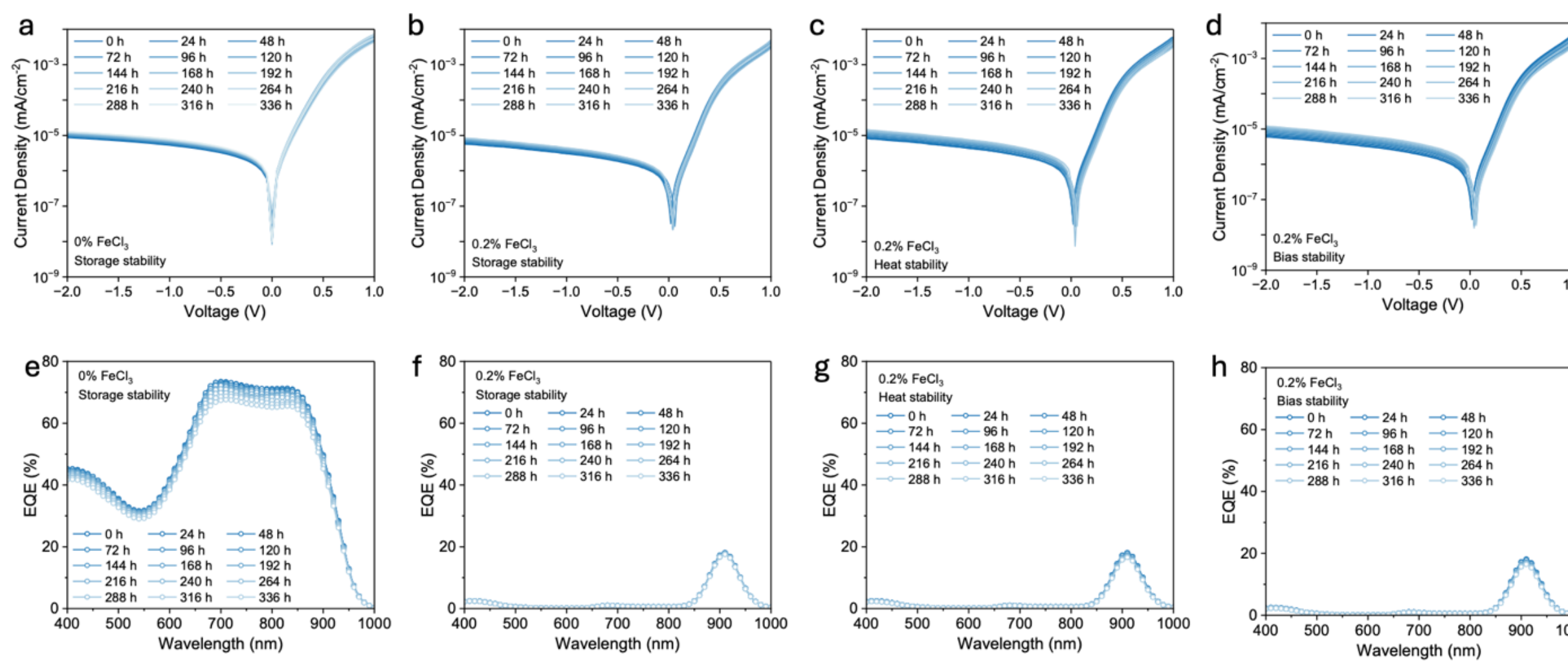

Fig. S35. Conventional devices stability test. (**a-d**) J_d spectra and (e-h) *EQE* spectra of 0 wt% $FeCl_3$ at storage and 0.2 wt% $FeCl_3$ at storage, 60 °C, or -2 V continued bias condition.

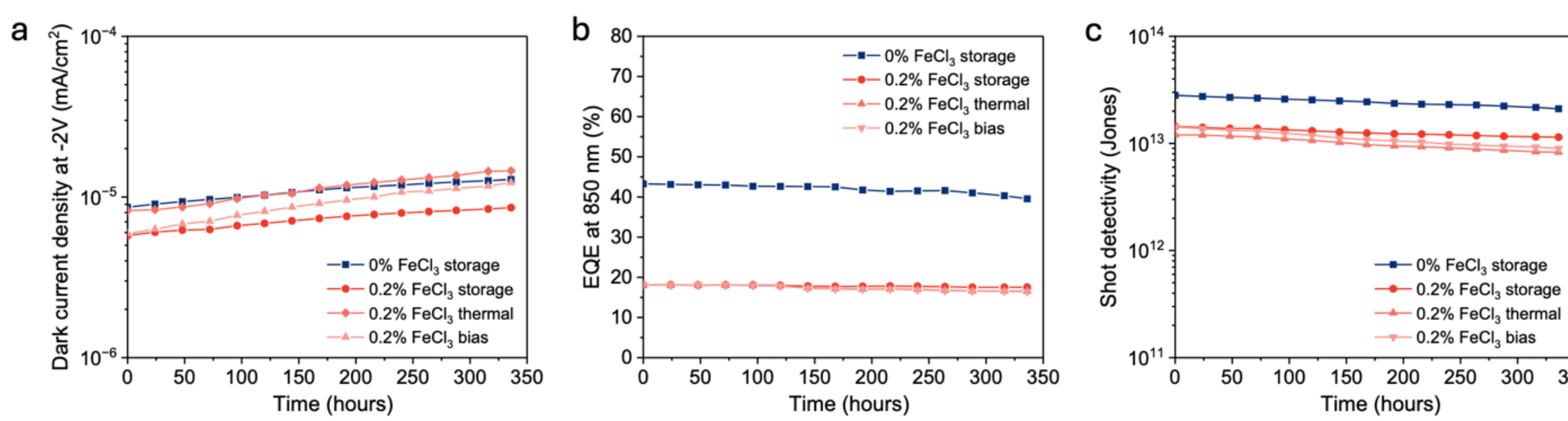

Fig. S36. Summarized conventional device stability results. (a) J_d at -2 V, (**b**) *EQE* at -2 V and 910 nm, and (**c**) shot detectivity at -2 V and 910 nm for PM6:Y6 conventional narrowband devices with different $FeCl_3$ concentrations at different conditions.

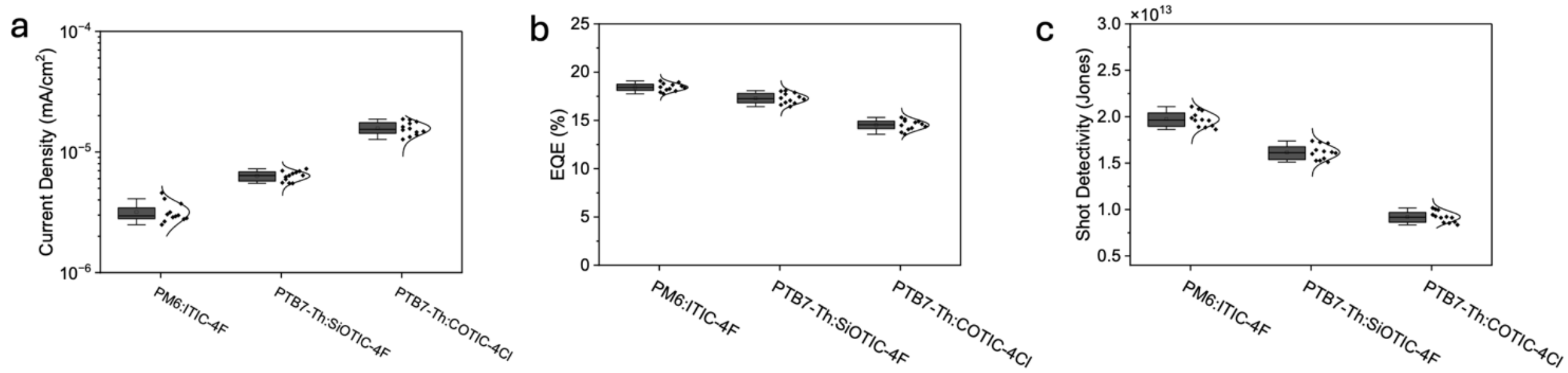

Fig. S37. Statistical distribution. Variation of (**a**) J_d at -2 V, (**b**) *EQE* at -2 V and peak position, and (**c**) shot detectivity at -2 V and peak position for conventional narrowband devices with different active layer materials.

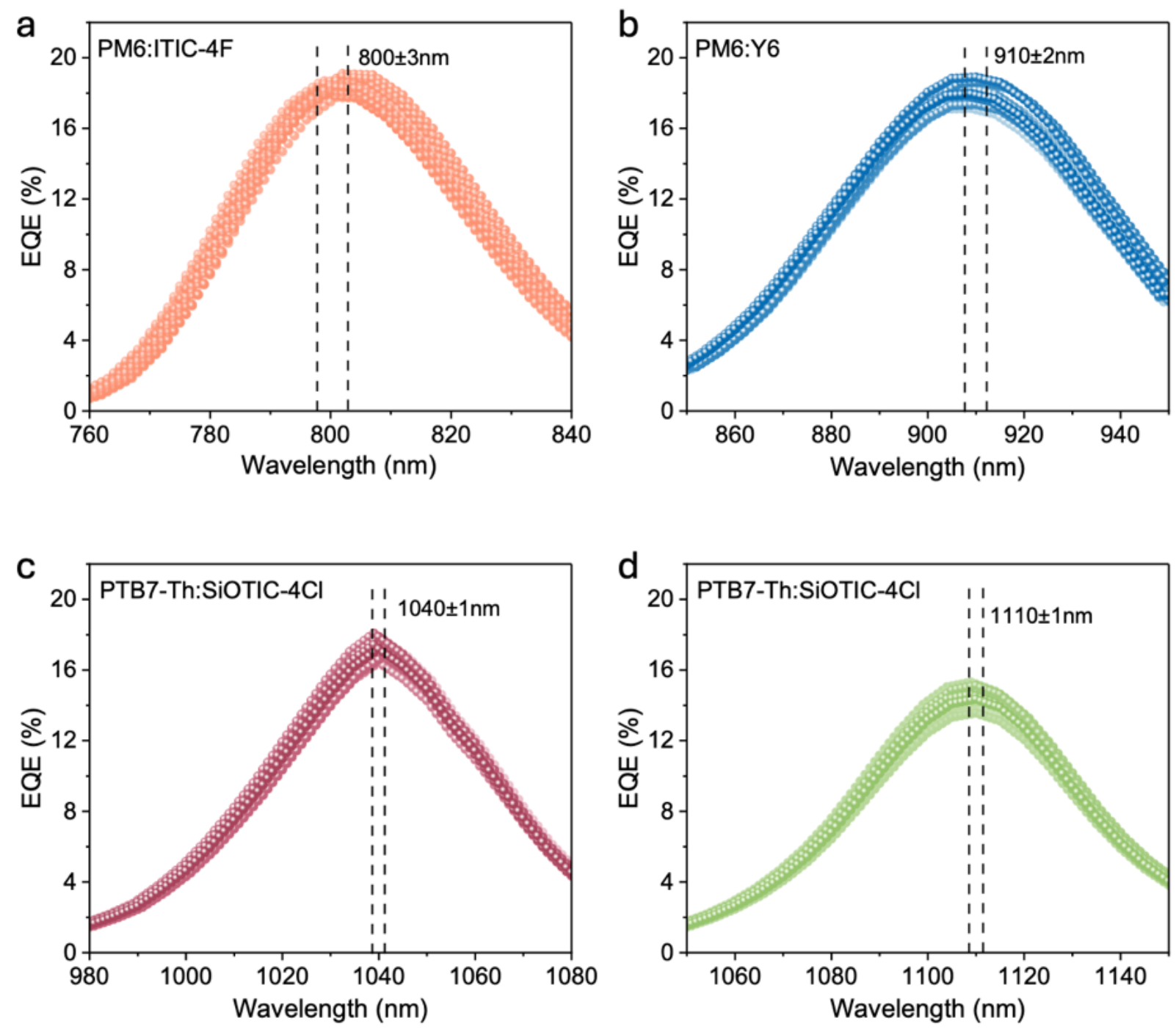

Fig. S38. Peak position variation. *EQE* spectra with 1 nm testing step of (**a**) PM6:ITIC-4F+0.05 wt% $FeCl_3$, (**b**) PM6:Y6+0.2 wt% $FeCl_3$, (**c**) PTB7-Th:SiOTIC-4Cl+0.1 wt% $FeCl_3$, and (**d**) PTB7-Th:COTIC-4Cl+0.1 wt% $FeCl_3$.

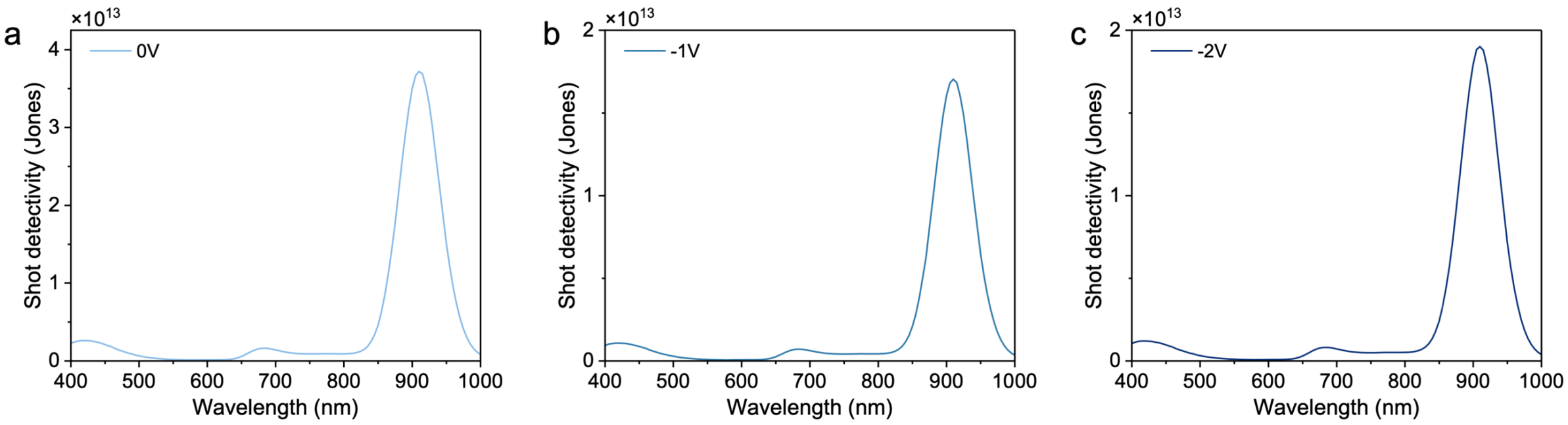

Fig. S39. Voltage-dependent shot-noise limited detectivity. Calculated shot detectivity of PM6:Y6+0.2 wt% $FeCl_3$ conventional OPDs (800 nm) at (**a**) 0 V, (**b**) -0.5 V, (**c**) -1 V, and (**d**) -2 V.

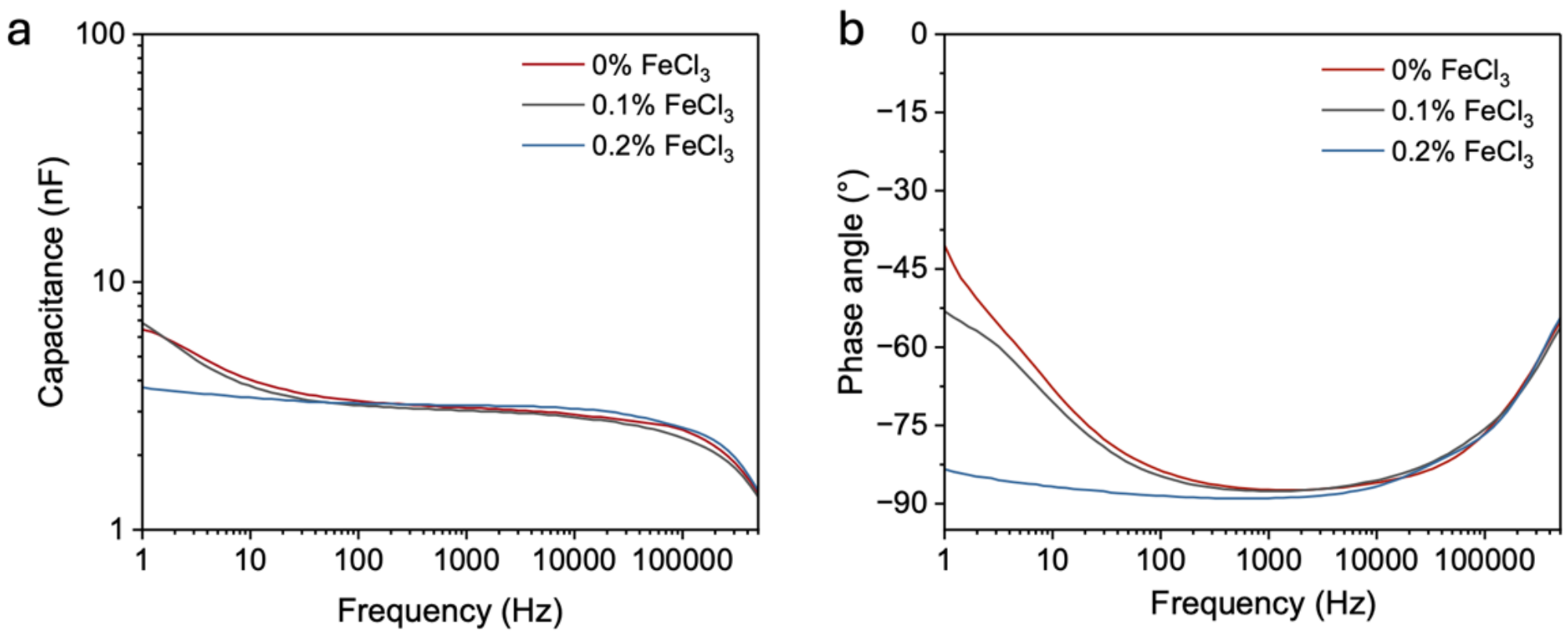

Fig. S40. Impedance analysis. (**a**) Capacitance-frequency and (**b**) phase angle-frequency spectra for conventional narrowband devices with different $FeCl_3$ concentrations.

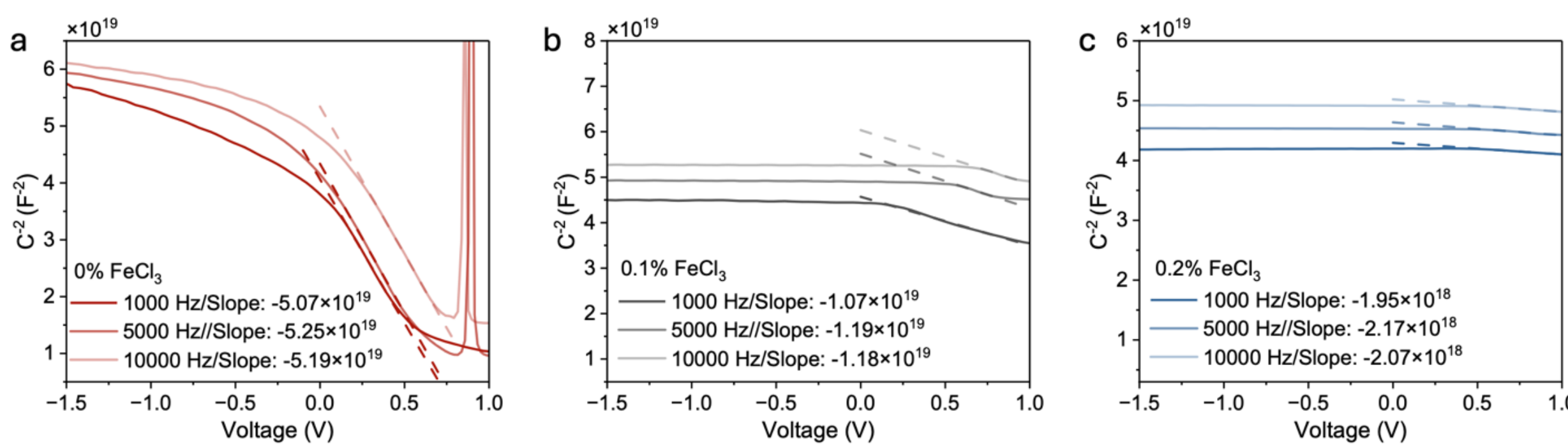

Fig. S41. Frequency-dependent Mott-Schottky analysis. Mott-Schottky plots of conventional narrowband devices with (**a-c**) 0-0.2 wt% $FeCl_3$ concentrations at 1, 5, 10 kHz. The solid lines represent measured data points and dashed lines represent linear fitting.

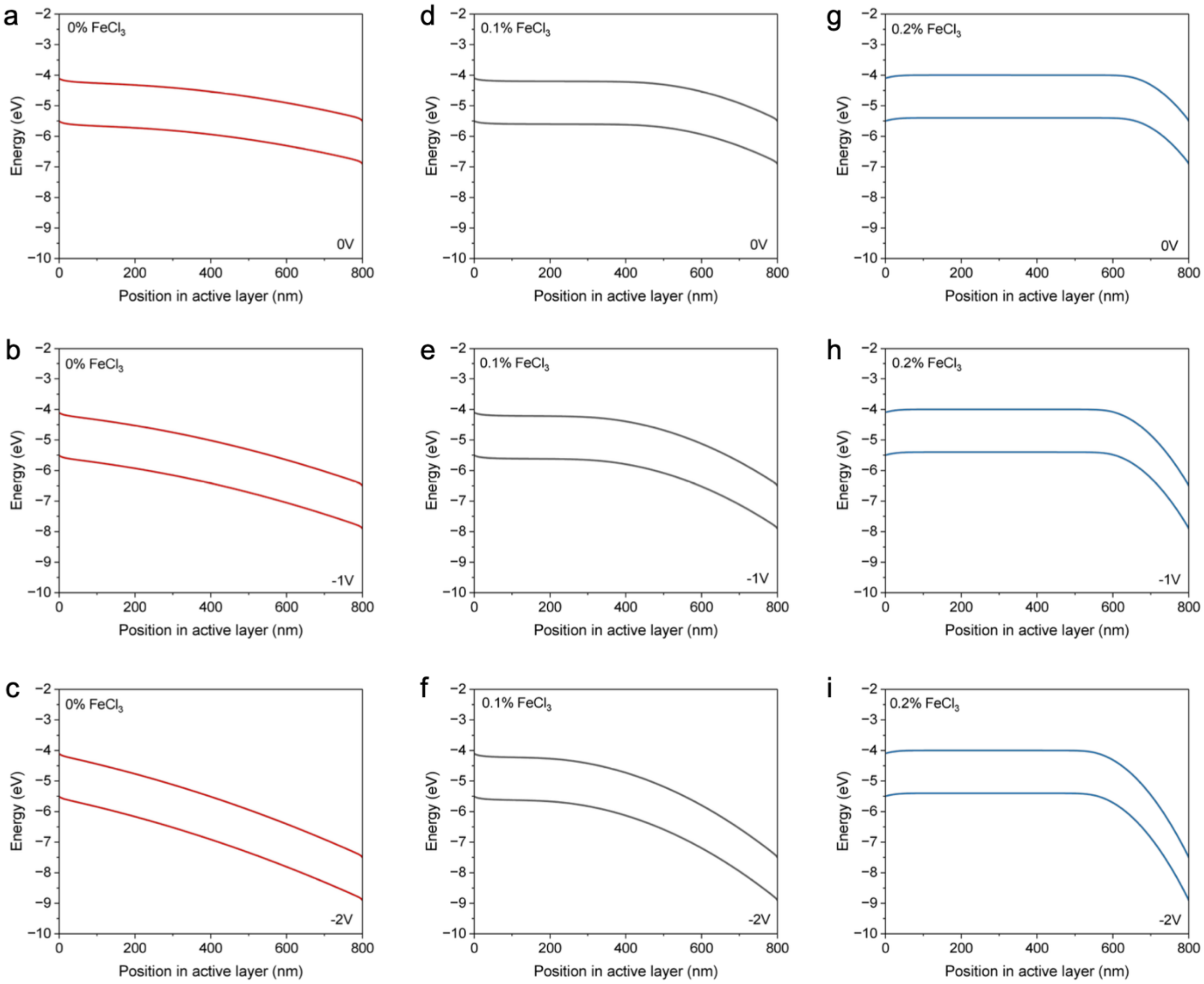

Fig. S42. Simulated band diagram under reverse bias. Computed energy band diagram from drift-diffusion simulation for (**a-d**) 0 wt% $FeCl_3$, (**e-h**) 0.1 wt% $FeCl_3$, (**i-l**) 0.2 wt% $FeCl_3$ at 0, -1, -2V. The device structure is ITO (100 nm)/POET-T2-COOH (100 nm)/PM6:Y6 (800 nm)/PFN-Br (10 nm)/Ag (100 nm).

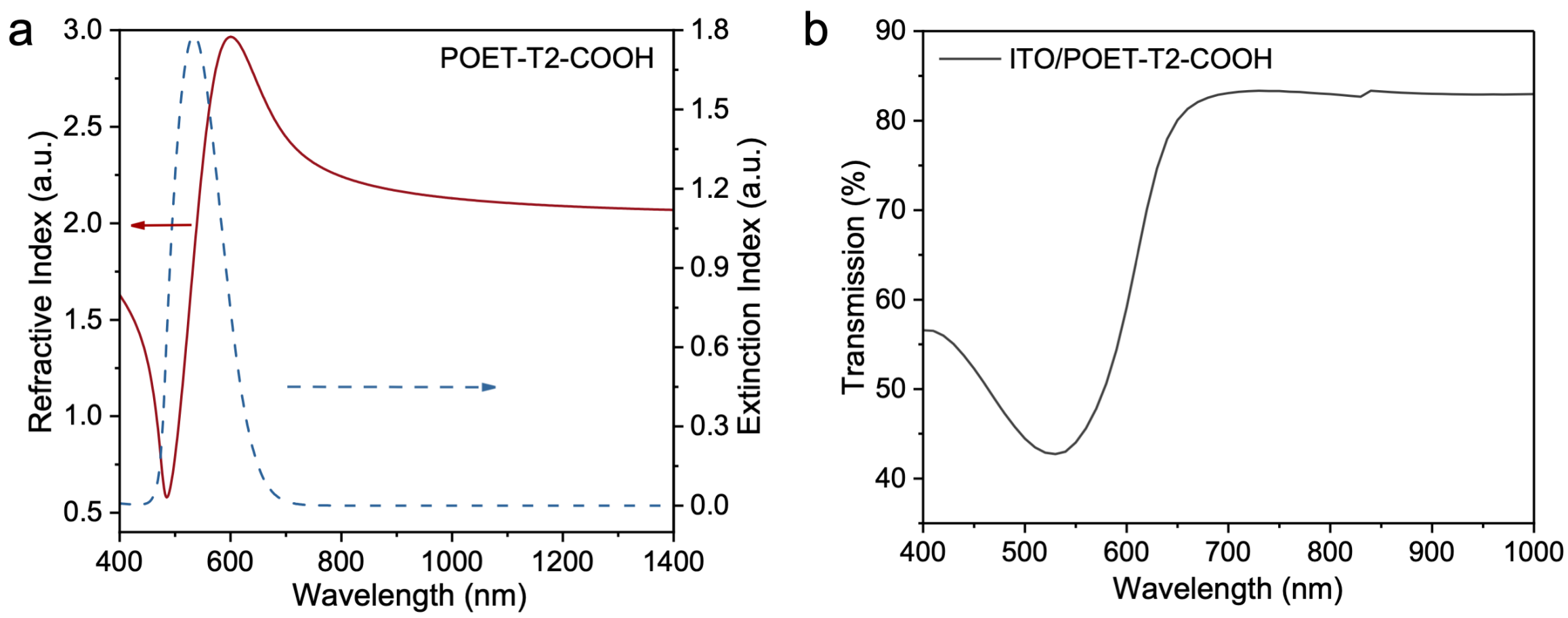

Fig. S43. Optical properties of POET-T2-COOH film. (**a**) Refractive index and extinction index, and (**b**) transmittance profile of POET-T2-COOH active layer. The POET-T2-COOH layer exhibits relatively strong absorption in the visible region, particularly around 400-600 nm. In contrast, in the near-infrared (NIR) region ($\lambda > 800$ nm), the extinction coefficient is negligible and the film remains highly transparent, indicating minimal optical loss for long-wavelength photons.

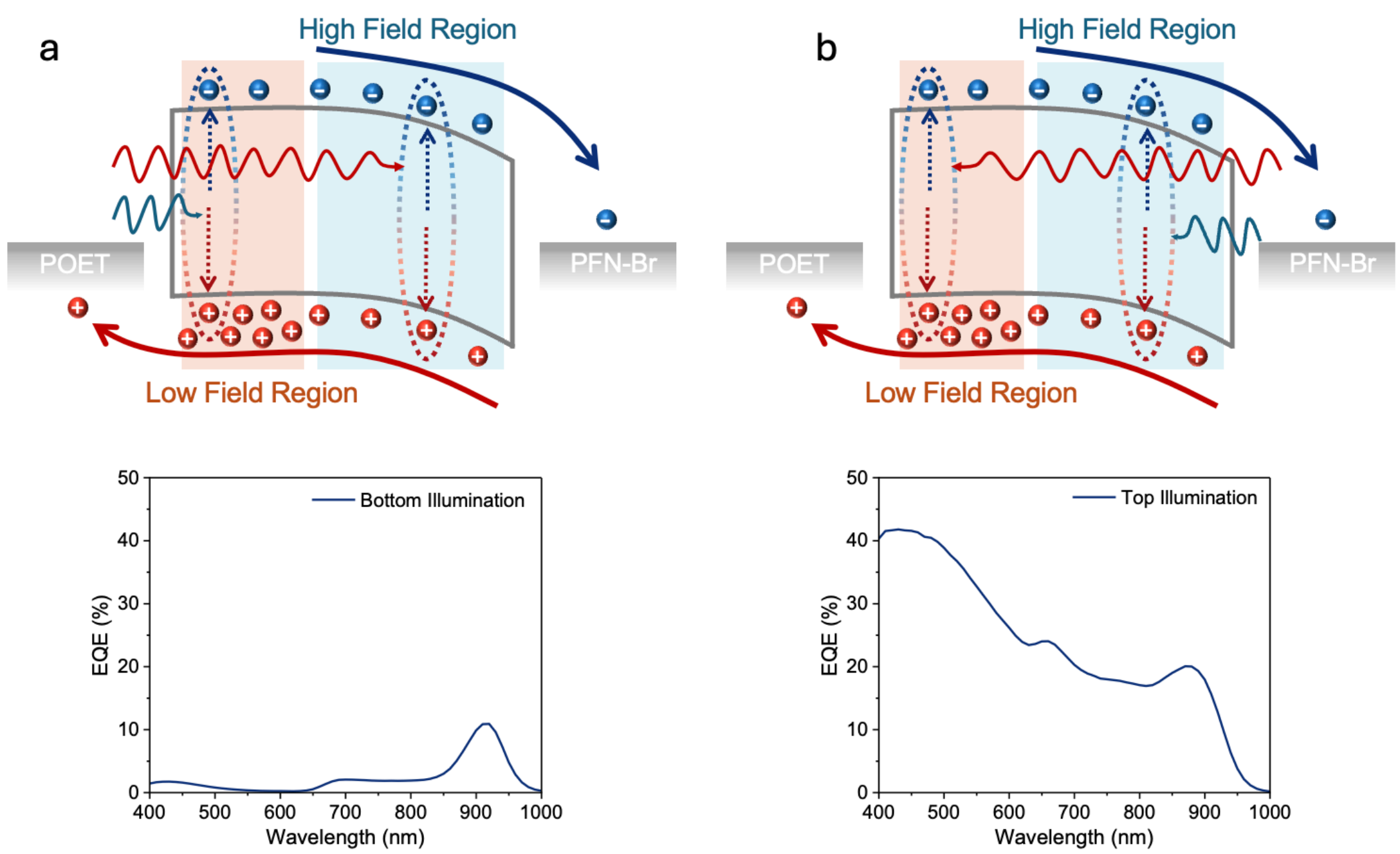

Fig. S44. Illumination-direction dependent photoresponse. *EQE* spectra of semitransparent devices (ITO/POET-T2-COOH/PM6:Y6+0.2 wt% $FeCl_3$ (800 nm)/PFN-Br/Ag (15 nm)) from (**a**) bottom illumination and (**b**) top illumination.

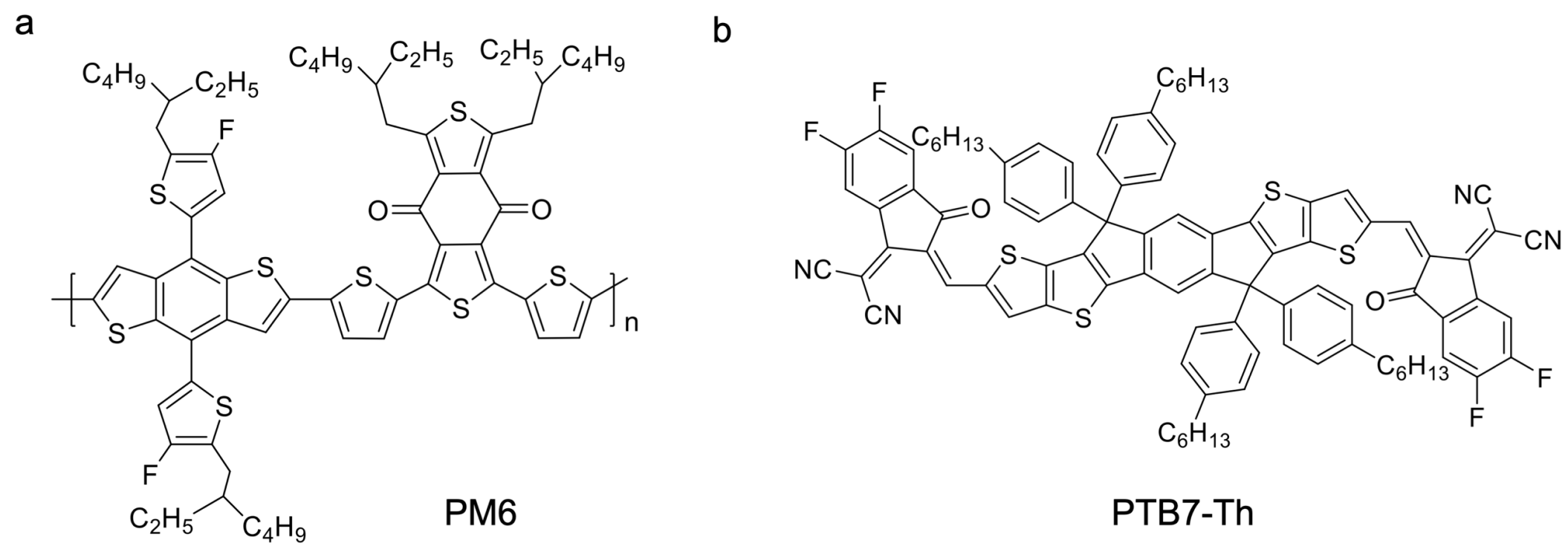

Fig. S45. Molecular structure of donor materials. (**a**) PM6 for ~800/~900 nm narrowband OPDs, and (**b**) PTB7-Th for ~1000/~1100 nm narrowband OPDs.

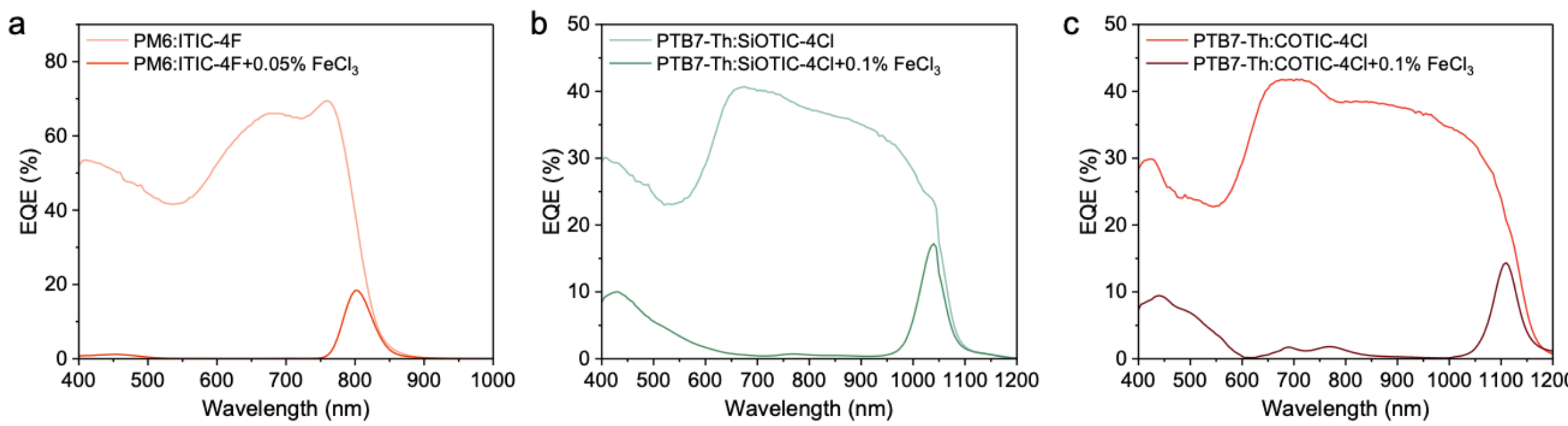

Fig. S46. Near-infrared narrowband OPDs performance. *EQE* response spectra at -2 V of (**a**) PM6:ITIC-4F/PM6:ITIC-4F+0.05 wt% $FeCl_3$, (**b**) PTB7-Th:SiOTIC-4Cl/PTB7-Th:SiOTIC-4Cl+0.1 wt% $FeCl_3$, and (**c**) PTB7-Th:COTIC-4Cl/PTB7-Th:COTIC-4Cl+0.1 wt% $FeCl_3$, corresponding to narrowband responses centered at ~800, ~1000, and ~1100 nm, respectively. The film thicknesses are 800 nm.

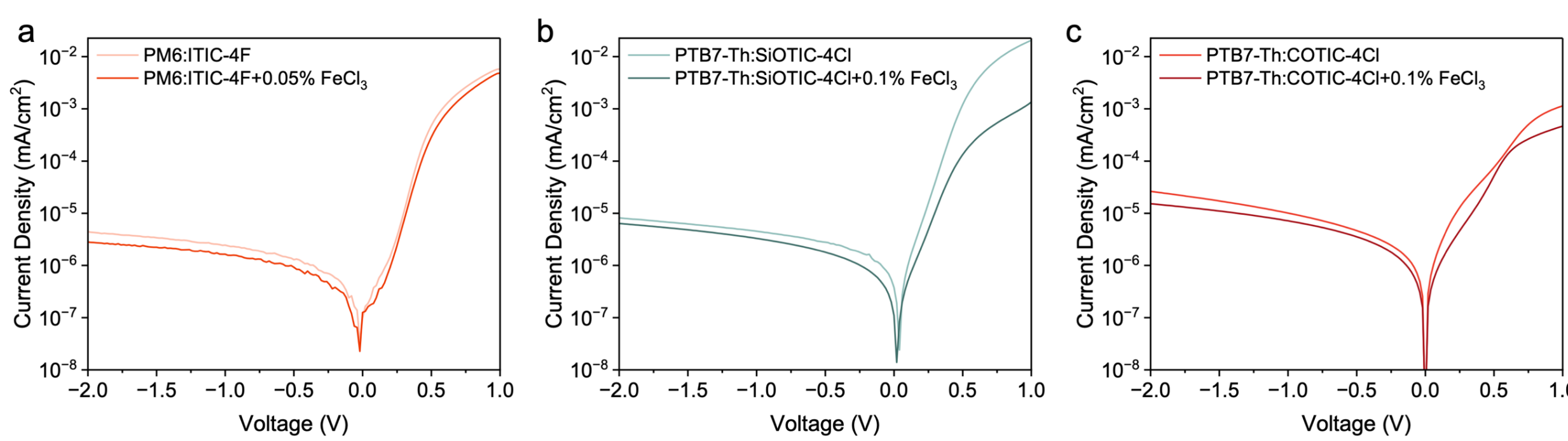

Fig. S47. Near-infrared narrowband OPDs performance. Current density-voltage characteristics of (**a**) PM6:ITIC-4F/PM6:ITIC-4F+0.05 wt% $FeCl_3$, (**b**) PTB7-Th:SiOTIC-4Cl/PTB7-Th:SiOTIC-4Cl+0.1 wt% $FeCl_3$, and (**c**) PTB7-Th:COTIC-4Cl/PTB7-Th:COTIC-4Cl+0.1 wt% $FeCl_3$.

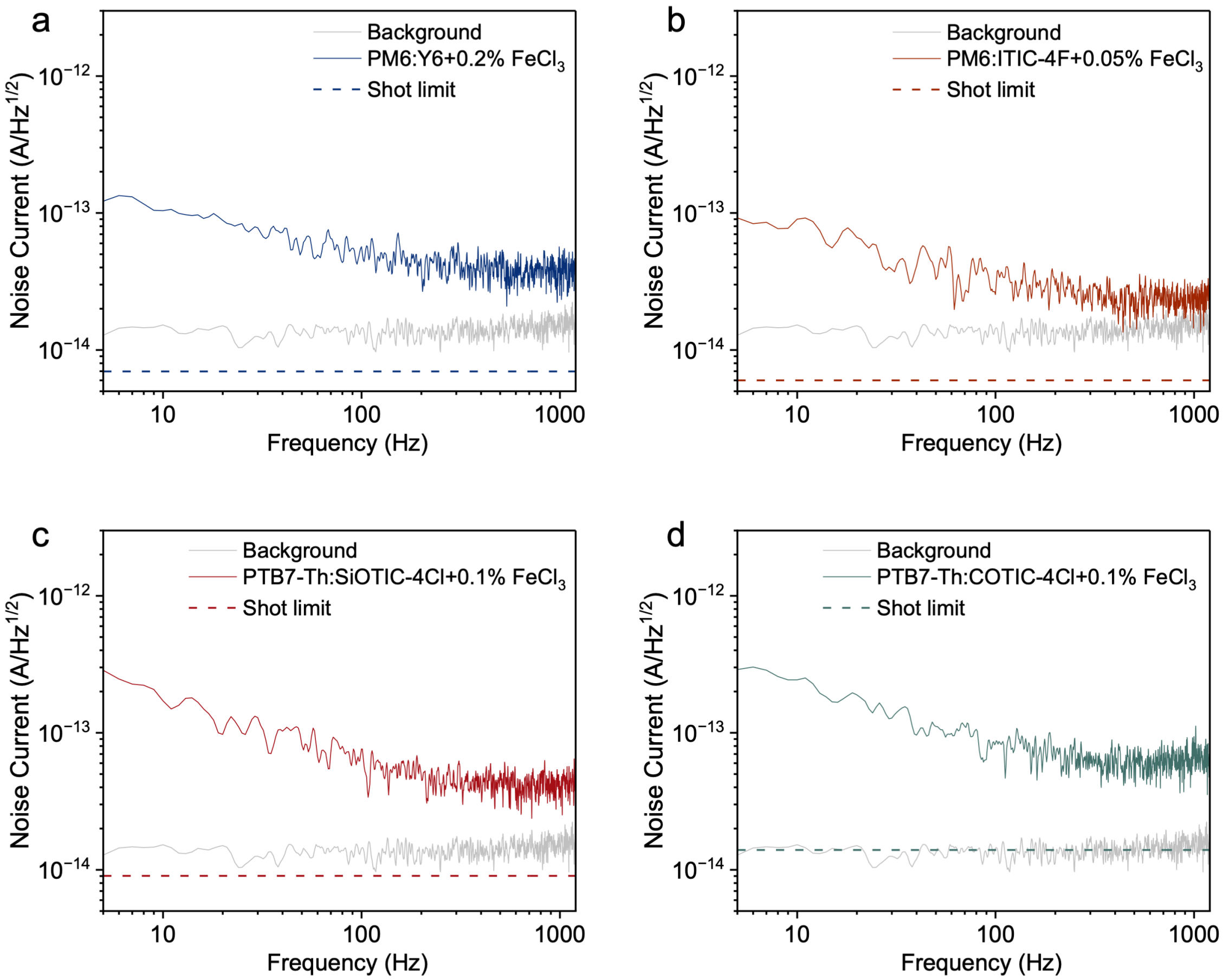

Fig. S48. Near-infrared narrowband OPDs performance. Noise current spectra of (**a**) PM6:Y6+0.2 wt% $FeCl_3$, (**b**) PM6:ITIC-4F+0.05 wt% $FeCl_3$, (**c**) PTB7-Th:SiOTIC-4Cl+0.1 wt% $FeCl_3$, and (**d**) PTB7-Th:COTIC-4Cl+0.1 wt% $FeCl_3$ under -2 V reverse bias. The grey line marks the noise floor of the background, and the dashed lines in the spectrum represent the shot-noise limit for each device.

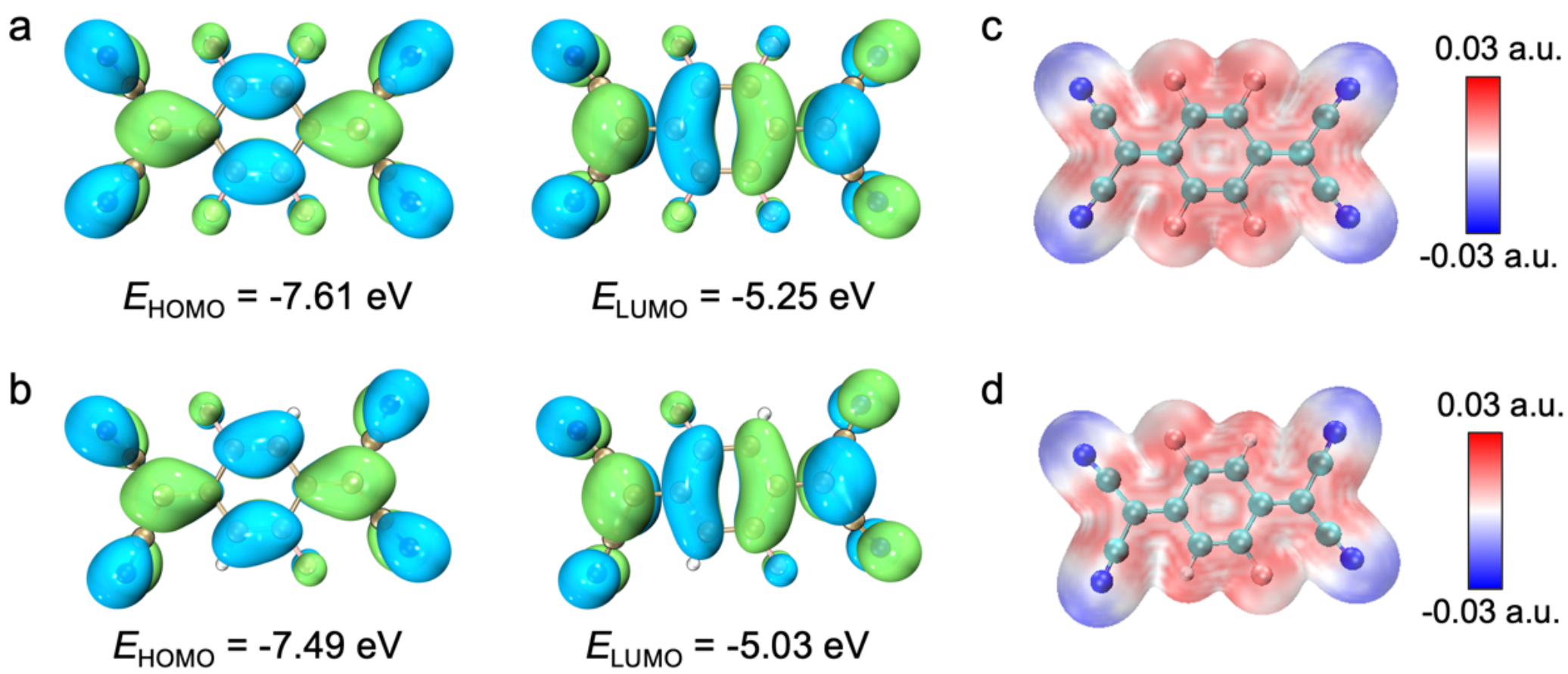

Fig. S49. Density functional theory simulation for *p*-dopants. HOMO/LUMO energy level for (**a**) F_4TCNQ and (**b**) F_2TCNQ. Electrostatic potentials distribution for (**c**) F_4TCNQ and (**d**) F_2TCNQ.

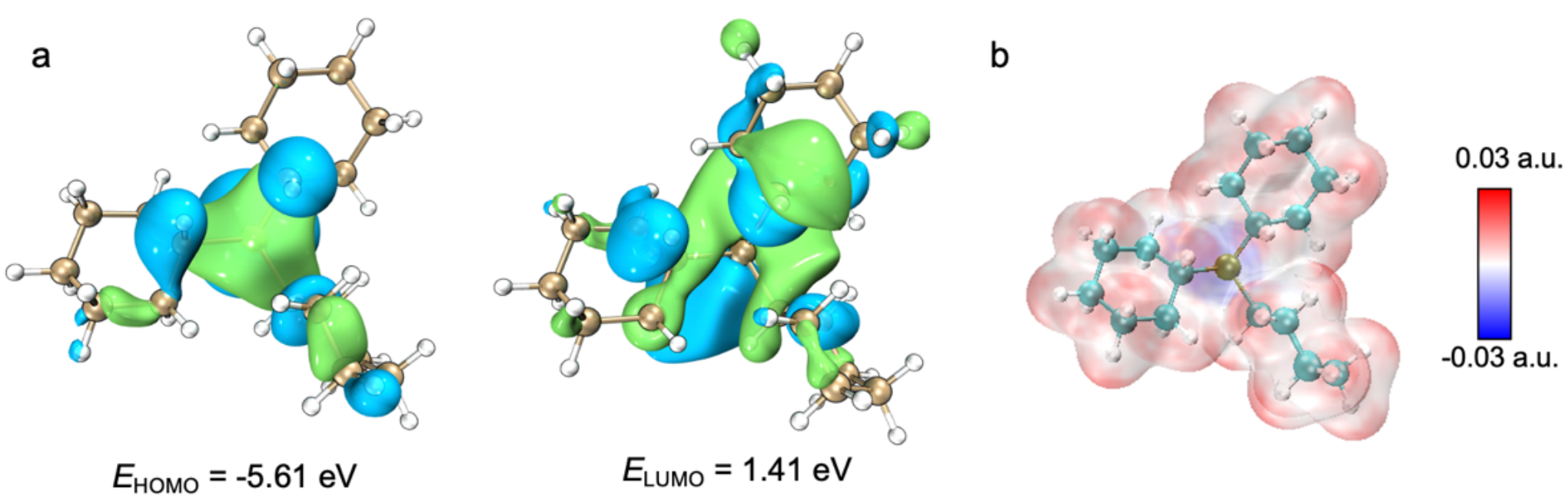

Fig. S50. Density functional theory simulation for *n*-dopants. (**a**) HOMO/LUMO energy level, and (**b**) electrostatic potentials distribution for $P(Cy)_3$.

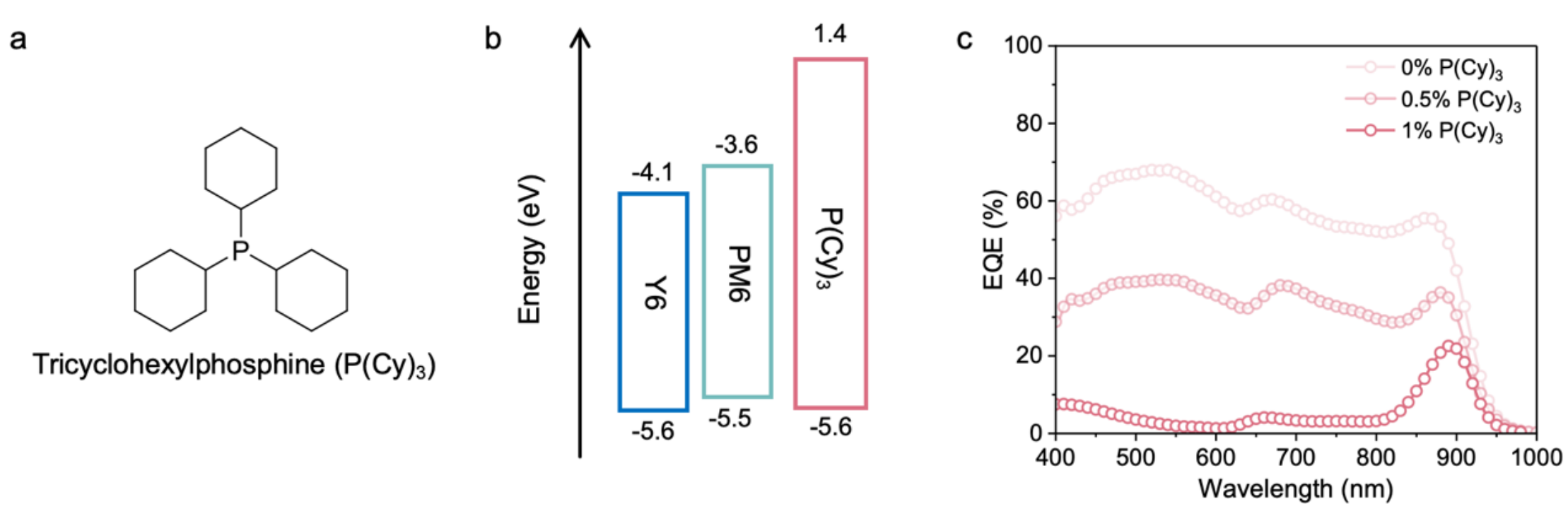

Fig. S51. Narrowband inverted OPDs performance. (**a**) Molecular structure and (**b**) energy level of *n*-dopant $P(Cy)_3$. (**c**) *EQE* response spectra of PM6:Y6 inverted OPDs (800 nm) with 0-1 wt% $P(Cy)_3$.

Table S1. Summarized calculated parameters in GIWAXS.

Film	Peak	Location [Å^{-1}]	*d*-spacing [Å]	L_c [Å]	*g* [%]
PM6	π-π peak	1.716	3.662	15.87	18.18
	Lamellar peak (PM6)	0.297	21.16	71.98	20.52
Y6	π-π peak	1.749	3.591	24.82	14.40
	Lamellar peak (Y6)	0.291	21.61	60.01	22.71
PM6:Y6	π-π peak	1.768	3.553	25.32	14.18
	Lamellar peak (PM6)	0.309	20.33	86.25	18.38
	Lamellar peak (Y6)	0.293	21.44	91.12	18.36
PM6:Y6 +0.1% $FeCl_3$	π-π peak	1.769	3.551	26.04	13.98
	Lamellar peak (PM6)	0.309	20.33	88.76	18.11
	Lamellar peak (Y6)	0.293	21.44	104.12	17.17
PM6:Y6 +0.3% $FeCl_3$	π-π peak	1.796	3.498	27.25	13.56
	Lamellar peak (PM6)	0.309	20.33	88.36	18.16
	Lamellar peak (Y6)	0.293	21.44	109.19	16.77
PM6:Y6 +0.5% $FeCl_3$	π-π peak	1.811	3.468	28.01	13.32
	Lamellar peak (PM6)	0.309	20.33	89.15	18.07
	Lamellar peak (Y6)	0.293	21.44	118.03	16.13
PM6:Y6 +0.7% $FeCl_3$	π-π peak	1.814	3.465	28.05	13.30
	Lamellar peak (PM6)	0.309	20.33	88.48	18.14
	Lamellar peak (Y6)	0.293	21.44	117.83	16.14
PM6:Y6 +1% $FeCl_3$	π-π peak	1.814	3.464	27.83	13.35
	Lamellar peak (PM6)	0.309	20.33	89.63	18.03
	Lamellar peak (Y6)	0.293	21.44	118.57	16.09

Table S2. Calculated carrier concentration and high-field region width from Mott-Schottky analysis.

Device	$FeCl_3$ concentration (%)	Slope [F^{-2}/V]	Carrier concentration[1] [cm^{-3}]	High-field region width[2] [nm] 0 V	-0.5 V	-1 V	-2 V
PM6:Y6 inverted OPDs (Broadband)	0%	-7.29×10^{18} ($\pm3.66\times10^{17}$)	$(3.45\pm0.14)\times10^{15}$	338 ±8.5	413 ±10.3	476 ±11.9	582 ±14.6
	0.1%	-5.53×10^{18} ($\pm3.16\times10^{17}$)	$(4.56\pm0.26)\times10^{15}$	294 ±8.4	362 ±10.2	417 ±11.7	511 ±14.4
	0.3%	-2.13×10^{18} ($\pm1.44\times10^{17}$)	$(1.18\pm0.08)\times10^{16}$	182 ±6.18	222 ±7.54	256 ±8.69	314 ±10.7
	0.5%	-5.46×10^{17} ($\pm4.32\times10^{16}$)	$(4.61\pm0.36)\times10^{16}$	93 ±3.67	113 ±4.46	130 ±5.13	159 ±6.28
	0.7%	-2.78×10^{17} ($\pm2.38\times10^{16}$)	$(9.05\pm0.77)\times10^{16}$	66 ±2.81	81 ±3.44	93 ±3.95	113 ±4.80
	1%	-1.10×10^{17} ($\pm9.68\times10^{15}$)	$(2.29\pm0.20)\times10^{17}$	42 ±1.85	51 ±2.24	58 ±2.55	71 ±3.12
PM6:Y6 conventional OPDs (Narrowband)	0%	-5.07×10^{19} ($\pm3.66\times10^{17}$)	$(4.97\pm0.26)\times10^{14}$	890 ±17.4	1097 ±21.3	1265 ±24.5	1547 ±30.0
	0.1%	-1.07×10^{19} ($\pm3.66\times10^{17}$)	$(2.35\pm0.17)\times10^{15}$	409 ±11.0	504 ±13.4	581 ±15.5	710 ±18.9
	0.2%	-1.95×10^{18} ($\pm3.66\times10^{17}$)	$(1.29\pm0.12)\times10^{16}$	175 ±5.39	215 ±6.60	249 ±7.59	304 ±9.26

[1]For estimation of carrier concentration, we used parameters $q = 1.602\times10^{-19}$ C, $\varepsilon_0 = 8.854\times10^{-12}$ F/m, $\varepsilon = 3.5$ for organic semiconductor materials, $A = 0.04$ cm^2.

[2]The built-in voltage ($V_{bi} = 1.02$ V) for high-field region width calculation was assumed to be the work function difference between cathode and anode.

Table S3. Overview of reported broadband OPDs under reverse bias.

Device	Bias	J_d [A/cm^2]	Wavelength [nm]	R [A/W]	Frequency [Hz]	Area [cm^2]	D^* [Jones]	Ref
PBDB-T:PNDI-FT10	-3 V	1.1×10^{-8}	650	0.34	N.A.	0.046	$\approx10^{11}$	[9]
PBDB-T:N2200	-2 V	1.4×10^{-8}	620	0.42	1000	0.023	6.2×10^{12}	[10]
D18:Y6	-2 V	1×10^{-5}	805	0.51	1	N.A.	5.5×10^{10}	[11]
ClAlPc:C_{60}	-2 V	3.6×10^{-10}	730	0.17	N.A.	N.A.	4.1×10^{12}	[12]
PTB7-Th:CO1-4Cl	-2 V	7×10^{-9}	960	0.50	100	0.0451	$\approx1\times10^{12}$	[13]
PBTQ(OD):PC_{71}BM	-2 V	2×10^{-7}	960	0.05	N.A.	0.03	3×10^{11} (D_{sh}^*)	[14]
P3HT:BZIC-2Cl	-2 V	1×10^{-7}	1030	0.31	90	0.045	$\approx1\times10^{12}$	[15]
CDT-TQ:PC_{71}BM	-2 V	6.8×10^{-6}	1100	0.07	400	0.04	6.6×10^{10}	[16]
PTTBAl:PC_{71}BM	-2 V	6×10^{-8}	1100	0.09	1000	0.04	$\approx2\times10^{10}$	[17]
PM6:Y6	-1 V	8.9×10^{-9}	810	0.30	1000	0.08	9.71×10^{11}	[18]
PTB7-Th:TQ_{pp}2FIC	-1 V	1.19×10^{-7}	900	0.23	60	0.04	$\approx2\times10^{12}$	[19]
PSQ-3,6-Cz:PC_{61}BM	-1 V	2.05×10^{-8}	860	0.13	1	0.045	3.83×10^{7}	[20]
PTB7-Th:COTIC-4Cl	-1 V	3.12×10^{-8}	1050	0.29	192	0.04	3.39×10^{11}	[21]
P3HT:IEICO-4F:PC61BM	-1 V	2.62×10^{-9}	850	0.21	1000	0.04	7.2×10^{12}	[22]
PBDB-T:Y6	-0.5 V	8.67×10^{-8}	860	0.50	N.A.	N.A.	3.0×10^{12} (D_{sh}^*)	[23]
PTB7-Th:COTCN2	-0.5 V	1.08×10^{-7}	1000	0.23	1600	0.05	1.3×10^{11}	[24]
**PM6:Y6+0.7% $FeCl_3$**	**-2 V**	**7.69×10^{-9}**	**850**	**0.44**	**1000**	**0.04**	**6.9×10^{12}**	**This work**

Table S4. Overview of reported narrowband OPDs under reverse bias

Device	Bias	Wavelength [nm]	*EQE* [%]	FWHM [nm]	Thickness [nm]	Frequency [Hz]	Area [cm^2]	D^* [Jones]	Ref
PM6:Y6	0	892	27	50	600	N.A.	0.06	$\approx 1.25\times10^{12}$	[25]
PCE10:SiOTIC-4F	0	1026	20	46	600	N.A.	0.06	$\approx 1.25\times10^{12}$	[25]
PCE10:COTIC-4F	0	1096	18	50	500	N.A.	0.06	$\approx 1.25\times10^{12}$	[25]
SQ-H:$PC_{61}BM$	0	1050	12.3	85	≈85	30	0.071	4×10^{10}	[26]
P3HT:PTB7/$PC_{71}BM$	0	745	≈0.45	≈50	≈750	N.A.	0.064	1×10^{12}	[27]
ZnPC:C60 (cavity)	0	950	18	36	60	N.A.	0.0644	3.5×10^{11}	[28]
PCDTPTSe:PC71BM (cavity)	0	710	18	55	≈200	N.A.	0.064	$\approx 5\times10^{11}$	[29]
J980 Dye	-0.5	980	3.2	50	10	N.A.	0.031	1.7×10^{8}	[30]
PM6:Y6	-0.5	900	≈18	47.8	900	1	N.A.	1.1×10^{12}	[31]
PM6:ITIC-4F/PTB7-Th:COTIC-4Cl:Y6	+1	814	≈12	40	3500/670	100	0.09	6×10^{11}	[32]
PM6:ITIC-4F/PTB7-Th:COTIC-4Cl:Y6	-1	1108	≈4	63	3500/670	100	0.09	6×10^{9}	[32]
SQ-H:$PC_{61}BM$	-1	1050	20.5	91	≈100	30	0.071	$\approx 1\times10^{10}$	[26]
PCDTBT:PC71BM	-1	670	35	85	2000	120	0.2	1.8×10^{12}	[33]
DPP-DTT:$PC_{71}BM$	-1	930	≈8	≈70	2000	120	0.2	4.7×10^{12}	[33]
ranDPP-2TBT:IEICO-4F	-2	916	≈18	68	1000	N.A.	0.045	$\approx 1\times10^{11}$	[34]
PD004:PD-A2	-2	1100	≈15	66	6000	N.A.	N.A.	1.9×10^{11}	[35]
F8T2:hPDI2	-3	575	1.7	22	780	N.A.	0.09	$\approx 2.5\times10^{10}$ (D_{sh}^*)	[36]
P3HT:PCBM:CdTe	-6	660	200	100	3500	35	0.07	$\approx 7\times10^{11}$	[37]
PD004:PD-A2	-8	1080	53	56	8200	N.A.	0.054	$\approx 1\times10^{10}$	[38]

ZnPc:C_{60}	-10	880	20	19	400	N.A.	0.644	≈6×10^{11} (D_{sh}^*)	[39]
P3HT:PTB7-Th:BEH	-13	850	15300	27	3000	N.A.	0.038	8.8×10^{11}	[40]
P3HT:PC_{61}BM	-60	665	1640	23	4000	10	0.038	≈4×10^{10}	[41]
**PM6:ITIC-4F**	**-2**	**800**	**18.4**	**50**	**800**	**1000**	**0.04**	**2.4×10^{12}**	**This work**
**PM6:Y6**	**-2**	**910**	**18.1**	**65**	**800**	**1000**	**0.04**	**1.93×10^{12}**	**This work**
**PTB7-Th:SiOTIC-4Cl**	**-2**	**1040**	**17.2**	**45**	**800**	**1000**	**0.04**	**1.77×10^{12}**	**This work**
**PTB7-Th:COTIC-4Cl**	**-2**	**1110**	**14.3**	**50**	**800**	**1000**	**0.04**	**1.01×10^{12}**	**This work**

Supplementary References

1. V. A. Trukhanov, V. V. Bruevich and D. Y. Paraschuk, "Effect of doping on performance of organic solar cells," *Physical Review B* 84, no. 20 (2011): 205318. 10.1103/PhysRevB.84.205318
2. S. Dongaonkar, J. D. Servaites, G. M. Ford, et al., "Universality of non-Ohmic shunt leakage in thin-film solar cells," *Journal of Applied Physics* 108, no. 12 (2010): https://doi.org/10.1063/1.3518509
3. N. Tokmoldin, J. Vollbrecht, S. M. Hosseini, et al., "Explaining the Fill-Factor and Photocurrent Losses of Nonfullerene Acceptor-Based Solar Cells by Probing the Long-Range Charge Carrier Diffusion and Drift Lengths," *Advanced Energy Materials* 11, no. 22 (2021): 2100804. https://doi.org/10.1002/aenm.202100804
4. O. J. Sandberg, C. Kaiser, S. Zeiske, et al., "Mid-gap trap state-mediated dark current in organic photodiodes," *Nature Photonics* 17, no. 4 (2023): 368. https://doi.org/10.1038/s41566-023-01173-5
5. X. Ma, H. Bin, B. T. van Gorkom, et al., "Identification of the Origin of Ultralow Dark Currents in Organic Photodiodes," *Advanced Materials* 35, no. 8 (2023): 2209598. https://doi.org/10.1002/adma.202209598
6. J. Kublitski, A. Hofacker, B. K. Boroujeni, et al., "Reverse dark current in organic photodetectors and the major role of traps as source of noise," *Nature Communications* 12, no. 1 (2021): 551. https://doi.org/10.1038/s41467-020-20856-z
7. G. Simone, M. J. Dyson, C. H. L. Weijtens, et al., "On the Origin of Dark Current in Organic Photodiodes," *Advanced Optical Materials* 8, no. 1 (2020): 1901568. https://doi.org/10.1002/adom.201901568
8. J. Shanahan, L. Yan, Y. Olanrewaju, et al., "Acid-Triggered Side Chain Cleavage Leads to Doped Conjugated Polymers of High Conductivity," *Journal of the American Chemical Society* 146, no. 47 (2024): 32243. https://doi.org/10.1021/jacs.4c09843
9. X. Xu, X. Zhou, K. Zhou, et al., "Large-Area, Semitransparent, and Flexible All-Polymer Photodetectors," *Advanced Functional Materials* 28, no. 48 (2018): 1805570. https://doi.org/10.1002/adfm.201805570
10. H. Lin, B. Xu, J. Wang, et al., "Novel Dark Current Reduction Strategy via Deep Bulk Traps for High-Performance Solution-Processed Organic Photodetectors," *ACS Applied Materials & Interfaces* 14, no. 30 (2022): 34891. https://doi.org/10.1021/acsami.2c04981
11. Y. Wei, H. Chen, T. Liu, et al., "Self-Powered Organic Photodetectors with High Detectivity for Near Infrared Light Detection Enabled by Dark Current Reduction," *Advanced Functional Materials* 31, no. 52 (2021): 2106326. https://doi.org/10.1002/adfm.202106326
12. M. Y. Hadiyanto, R. Estrada, C.-C. Lee, et al., "Transparent photodetectors with ultra-low dark current and high photoresponse for near-infrared detection," *Organic Electronics* 99, no. (2021): 106356. https://doi.org/10.1016/j.orgel.2021.106356
13. J. Huang, J. Lee, J. Vollbrecht, et al., "A High-Performance Solution-Processed Organic Photodetector for Near-Infrared Sensing," *Advanced Materials* 32, no. 1 (2020): 1906027. https://doi.org/10.1002/adma.201906027
14. F. Verstraeten, S. Gielen, P. Verstappen, et al., "Efficient and readily tuneable near-infrared photodetection up to 1500 nm enabled by thiadiazoloquinoxaline-based push–pull type conjugated polymers," *Journal of Materials Chemistry C* 8, no. 29 (2020): 10098. http://dx.doi.org/10.1039/D0TC01435D

15. Z. Qiao, S. Sharma, Y. He, et al., “Short-Wavelength Infrared Imaging with Organic Photodetectors Based on Non-Fullerene Acceptors with Detection above 1200 nm,” *Advanced Materials* (2026): e22600. https://doi.org/10.1002/adma.202522600
16. T. Bills, C.-T. Liu, J. Lim, et al., “A General Strategy for Enhancing Sensitivity and Suppressing Noise in Infrared Organic Photodetectors Using Non-Conjugated Polymer Additives,” *Advanced Functional Materials* 34, no. 24 (2024): 2314210. https://doi.org/10.1002/adfm.202314210
17. S. Gielen, C. Kaiser, F. Verstraeten, et al., “Intrinsic Detectivity Limits of Organic Near-Infrared Photodetectors,” *Advanced Materials* 32, no. 47 (2020): 2003818. https://doi.org/10.1002/adma.202003818
18. L. Lei, H. Zhen, Z. Peng, et al., “Advancing Intrinsically Stretchable Organic Photodetectors With a Thermoplastic Polyester Elastomer,” *Advanced Functional Materials* (2026): e27213. https://doi.org/10.1002/adfm.202527213
19. J. Xiao, Y. Wang, L. Yuan, et al., “Engineering Carrier Barriers To Suppress Dark Current in Near-Infrared Organic Photodiodes,” *ACS Applied Materials & Interfaces* 18, no. 6 (2026): 10119. https://doi.org/10.1021/acsami.5c21038
20. J. He, Z. Wang, Y. Gao, et al., “Near-Infrared Organic Photodetectors with Ultralow Dark Currents Based on Carbazole-bridged Pyrrolic Polysquaraines,” *Advanced Optical Materials* 12, no. 2 (2024): 2301361. https://doi.org/10.1002/adom.202301361
21. J. Yi, Y. Yang, Y. Pei, et al., “Trap-Modified Inverted Organic Photodetectors via Layer-by-Layer Processing with Poly(N-vinylcarbazole) Additives,” *Advanced Functional Materials* 36, no. 28 (2026): e19588. https://doi.org/10.1002/adfm.202519588
22. B.-H. Jiang, F.-C. Hsiao, Y.-R. Lin, et al., “Highly Efficient Ternary Near-Infrared Organic Photodetectors for Biometric Monitoring,” *ACS Applied Materials & Interfaces* 15, no. 8 (2023): 10907. https://doi.org/10.1021/acsami.2c20527
23. Q. Liu, Y. Wang, L. Yuan, et al., “Thermally Induced Anti-Aggregation Evolution of Thick Bulk-Heterojunction for vis–NIR Organic Photodetectors,” *Advanced Optical Materials* 10, no. 12 (2022): 2200340. https://doi.org/10.1002/adom.202200340
24. J.-W. Ha, H. J. Eun, B. Park, et al., “Effect of Cyano Substitution on Non-Fullerene Acceptor for Near-Infrared Organic Photodetectors above 1000 nm,” *Advanced Functional Materials* 33, no. 8 (2023): 2211486. https://doi.org/10.1002/adfm.202211486
25. Q. Liu, S. Zeiske, X. Jiang, et al., “Electron-donating amine-interlayer induced n-type doping of polymer:nonfullerene blends for efficient narrowband near-infrared photo-detection,” *Nature Communications* 13, no. 1 (2022): 5194. https://doi.org/10.1038/s41467-022-32845-5
26. J. H. Kim, A. Liess, M. Stolte, et al., “An Efficient Narrowband Near-Infrared at 1040 nm Organic Photodetector Realized by Intermolecular Charge Transfer Mediated Coupling Based on a Squaraine Dye,” *Advanced Materials* 33, no. 26 (2021): 2100582. https://doi.org/10.1002/adma.202100582
27. S. Xing, X. Wang, E. Guo, H. Kleemann and K. Leo, “Organic Thin-Film Red-Light Photodiodes with Tunable Spectral Response Via Selective Exciton Activation,” *ACS Applied Materials & Interfaces* 12, no. 11 (2020): 13061. https://doi.org/10.1021/acsami.9b22058
28. B. Siegmund, A. Mischok, J. Benduhn, et al., “Organic narrowband near-infrared photodetectors based on intermolecular charge-transfer absorption,” *Nature Communications* 8, no. 1 (2017): 15421. https://doi.org/10.1038/ncomms15421

29. J. Wang, S. Ullbrich, J.-L. Hou, et al., "Organic Cavity Photodetectors Based on Nanometer-Thick Active Layers for Tunable Monochromatic Spectral Response," *ACS Photonics* 6, no. 6 (2019): 1393. https://doi.org/10.1021/acsphotonics.9b00471
30. S. B. Anantharaman, K. Strassel, M. Diethelm, et al., "Exploiting supramolecular assemblies for filterless ultra-narrowband organic photodetectors with inkjet fabrication capability," *Journal of Materials Chemistry C* 7, no. 46 (2019): 14639. https://doi.org/10.1039/C9TC04773E
31. G. Bai, P. Liu, M. Guo, et al., "Bias-Switchable Broadband–Narrowband Organic Photodetectors Enabled by Vertical Phase Separation in Thick PM6: Y6 Bulk Heterojunctions," *ACS Applied Materials & Interfaces* 18, no. 8 (2026): 12877. https://doi.org/10.1021/acsami.5c23045
32. Y. Tang, Z. Wang, M. Gao, et al., "Dual-mode narrowband organic photodetectors for self-aligned imaging in NIR-I and NIR-II," *Nature Communications* 16, no. 1 (2025): 7144. https://doi.org/10.1038/s41467-025-62394-6
33. A. Armin, R. D. Jansen-van Vuuren, N. Kopidakis, P. L. Burn and P. Meredith, "Narrowband light detection via internal quantum efficiency manipulation of organic photodiodes," *Nature Communications* 6, no. 1 (2015): 6343. https://doi.org/10.1038/ncomms7343
34. M. Brunetta, Z. Qiao, A. Zamhuri, et al., "Narrowband Detection via Charge Collection Narrowing Organic Photodetector Enabled by Low Bandgap Random Terpolymer for Biometric Sensing," *Advanced Functional Materials* 35, no. 24 (2025): 2422637. https://doi.org/10.1002/adfm.202422637
35. L.-H. Lai, W.-H. Lin, C.-C. Hsieh and M. A. Loi, "Bias switchable narrowband/broadband NIR organic photodetector fabricated with a scalable technique," *RSC Applied Interfaces* 1, no. 1 (2024): https://doi.org/10.1039/D3LF00089C
36. Y. Zhong, T. J. Sisto, B. Zhang, et al., "Helical Nanoribbons for Ultra-Narrowband Photodetectors," *Journal of the American Chemical Society* 139, no. 16 (2017): 5644. https://doi.org/10.1021/jacs.6b13089
37. L. Shen, Y. Fang, H. Wei, Y. Yuan and J. Huang, "A Highly Sensitive Narrowband Nanocomposite Photodetector with Gain," *Advanced Materials* 28, no. 10 (2016): 2043. https://doi.org/10.1002/adma.201503774
38. K.-W. Tsai, G. Madhaiyan, L.-H. Lai, et al., "Bulk-Heterojunction Adjustment Enables a Self-filtering Organic Photodetector with a Narrowband Response," *ACS Applied Materials & Interfaces* 14, no. 33 (2022): 38004. https://doi.org/10.1021/acsami.2c08336
39. J. Kublitski, A. Fischer, S. Xing, et al., "Enhancing sub-bandgap external quantum efficiency by photomultiplication for narrowband organic near-infrared photodetectors," *Nature Communications* 12, no. 1 (2021): 4259. https://doi.org/10.1038/s41467-021-24500-2
40. M. Liu, J. Wang, Z. Zhao, et al., "Ultra-Narrow-Band NIR Photomultiplication Organic Photodetectors Based on Charge Injection Narrowing," *The Journal of Physical Chemistry Letters* 12, no. 11 (2021): 2937. https://doi.org/10.1021/acs.jpclett.1c00330
41. J. Miao, F. Zhang, M. Du, W. Wang and Y. Fang, "Photomultiplication type narrowband organic photodetectors working at forward and reverse bias," *Physical Chemistry Chemical Physics* 19, no. 22 (2017): 14424. https://doi.org/10.1039/C7CP01969F
